# Supplementary material for: Sustainable production of benzene from lignin
Source: Nat Commun. 2021 Jul 26;12:4534. doi: 10.1038/s41467-021-24780-8 (PMC8313573; doi:10.1038/s41467-021-24780-8)
Supplement: Supplementary file 1 — Supplementary Information [file 41467_2021_24780_MOESM1_ESM.pdf]

Supplementary Information for

Sustainable production of benzene from lignin

Qinglei Meng[1]*, Jiang Yan[1, 2], Ruizhi Wu[1, 2], Huizhen Liu[1, 2], Yang Sun[3], NingNing Wu[3], Junfeng Xiang[3], Lirong Zheng[4], Jing Zhang[4], Buxing Han[1, 2, 4, 5]*

[1]Beijing National Laboratory for Molecular Sciences, CAS Laboratory of Colloid and Interface and Thermodynamics, CAS Research/Education Center for Excellence in Molecular Sciences, Institute of Chemistry, Chinese Academy of Sciences, Beijing 100190, China.

[2]School of Chemical Science, University of Chinese Academy of Sciences, Beijing 100049, China.

[3]Center for Physicochemical Analysis and Measurement, Chinese Academy of Sciences, Beijing 100190, China.

[4]Institute of High Energy Physics, Chinese Academy of Sciences, Beijing 100049, China.

[5]Shanghai Key Laboratory of Green Chemistry and Chemical Processes, School of Chemistry and Molecular Engineering, East China Normal University, Shanghai 200062, China.

*Corresponding author. Email: mengqinglei@iccas.ac.cn (Q. M.); hanbx@iccas.ac.cn (B. H.)

Supplementary Methods

Materials

Anisole (99.0%), biphenyl (99.0%), phenol (99.0%), benzene (99.0%), 4-isopropylanisole (95.0%), n-propylbenzene (98.0%), ruthenium chloride hydrate (99.9%), ruthenium (IV) oxide (99.9%), ruthenium power (99.99%), tungsten (VI) oxide (99.9%), tungsten power (99.9%), amorphous silicon oxide, formaldehyde (37%), isopropyl alcohol (99.5%), 4'-Methoxypropiophenone (99%), 4'-methoxyacetophenone (99%), 3', 4'-dimethoxyacetophenone (98+%), 3', 4', 5'-trimethoxyacetophenone (99%), 4'-hydroxy-3'-methoxyacetophenone (98%), 3,4-dimethoxybenzaldehyde (99%), 4-hydroxy-3-methoxybenzaldehyde (99%), 3, 4, 5-trimethoxybenzaldehyde (98%), 4'-hydroxy-3', 5'-dimethoxybenzaldehyde (97%), 2-methoxyphenol (98+%), 2, 6-Dimethoxyphenol (99%), bromoethane (98%), 2-bromopropane (99%), magnesium turnings (99+%), 1-phenylpropan-1-ol (99+%), paraformaldehyde (97%), 4-n-propylphenol (98%), nickel(II) nitrate hexahydrate (99.99%) and cobalt(II) nitrate hexahydrate (99.99%) were purchased from Alfa Aesar. tert-Butyl phenyl ether (98.0%) was obtained from Adamas Reagent Co., Ltd. 1-Methoxy-4-(prop-1-en-1-yl)benzene (98%) was purchased from Ark Pharm. Propylcyclohexane (98%) was provided by TCI (Shanghai) Development Co., Ltd. 4-Allyl-1, 2-dimethoxybenzene (99.0%), cyclohexane (99.8%) and 2-methoxy-4-propylphenol (99%) were purchased from Sigma-Aldrich, Inc.. Sodium borohydride (98%), bromine (99.5%), aluminum chloride (99%) and formaldehyde solution (37%) were provided by Sinopharm Chemical Reagent Co., Ltd. 2-(4'-methoxyphenyl)propane (98%), sodium chloride (99.5%), magnesium sulfate (99.5%), ammonium chloride (99.5%) and potassium carbonate (99%) were purchased from Aladdin Chemical Reagent Company. Iron(III) chloride hexahydrate (99+%), ammonium molybdate(VI) tetrahydrate (99+%), copper(II) nitrate hemipentahydrate (98+%), 4-hydroxybenzaldehyde (99%) and deuterium oxide (D_2O, 100.0 atom % D) were purchased from Acros Organics. 2, 6-Dimethoxy-4-propylphenol (95 %) was purchased from J & K Scientific Ltd. Ammonium metatungstate hydrate (99.9+% W) was provided by Strem Chemicals. Methyl Sulfoxide-d6[D; 99.9%, 0.03% (v/v) tetramethylsilane (TMS)] was purchased from Cambridge Isotope Laboratories, Inc. γ-Phasealuminum oxide (99.99%), methylmagnesium chloride solution (3.0 M in THF), dimethyl sulfate (99%) and 3', 5'-dimethoxy-4'-hydroxyacetophenone (98%) were obtained from Beijing InnoChem Science & Technology Co., Ltd. Methanol (99.9%), ethanol (99.5%), tetrahydrofuran (99.5%), ethyl acetate (99.5%), dichloromethane (99.5 %), N, N-dimethylformamide (99,5%), diethyl ether (99.5%) acetone (99.5%), pyridine (99.5%), ether (99.5%), *n*-hexane (95.0%) and petroleum ether (AR, boiling range 40-60°C) were obtained from Concord Technologies (Tianjin) Co. Ltd. Nitrogen (>99.99%), argon (>99.99%), hydrogen (>99.99%), hydrogen/argon (H_2 10%, 99.99%) and oxygen/argon (O_2 1%, 99.99%) were provided by Beijing Analytic Instrument Company. Deionized water was provided by the Institute of Chemistry, Chinese Academy of Sciences. HY_3 (Si/Al = 3), HY_{15}, HY_{30}, HY_{40} and $Mordenite_{10}$ (ammonium type) were obtained from Alfa Aesar. HY_5, $ZSM\text{-}5_{14}$, $ZSM\text{-}5_{18}$, $ZSM\text{-}5_{30}$, $ZSM\text{-}5_{35}$, $ZSM\text{-}5_{65}$, $ZSM\text{-}5_{235}$ (hydrogen type), $Beta_{25}$ (hydrogen type), $SAPO\text{-}34_{0.25}$ (hydrogen type) and MCM-41 (all-silicon) zeolites were purchased from Nankai University Catalyst Co. Ltd. Pine, poplar, willow, eucalyptus, cedar, peach, cedrela and applewood were obtained from Gansu Province, China. Phyllostachys pubescens was obtained from Jiangxi Province, China.

Synthesis of the model compounds

The syntheses of the model compounds were performed using the methods reported[1, 2]. ^{1}H NMR and ^{13}C NMR analyses were performed on a Bruker Avance III 400 HD using DMSO-d6 as the solvent. ^{1}H chemical shifts were referenced to TMS at 0 ppm, and ^{13}C chemical shifts were referenced to DMSO-d6 at 39.6 ppm. Multiplicities are described using the following abbreviations: s, singlet; d, doublet; t, triplet; q, quartet; and m, multiplet. The details of the methods and procedures for the syntheses of the model compounds are described as follows:

1-(4-Methoxyphenyl)-1-propanol (**1a**)

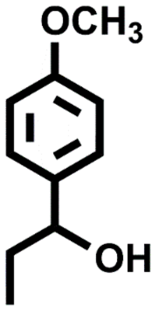

Sodium borohydride (3.8 g, 100 mmol) was added into the solution of 4'-methoxypropiophenone (16.4 g, 100 mmol) in THF/H_2O (100 mL/30 mL) at 0 °C. The reaction mixture was stirred at room temperature for 24 h. After the reaction, the mixture was sat for 10 min, and then the organic layer was separated. Ethyl acetate (50 mL) was added into the stirred organic layer at room temperature. The organic layer was then successively washed with deionized water (50 mL×3) and saturated brine (50 mL), dried by anhydrous $MgSO_4$. After the concentration in vacuum rotavap, 15.1 g of **1a** was obtained finally. ^{1}H NMR (400 MHz, DMSO-d6): δ 7.24~7.22 (d, 2H), 6.88~6.86 (d, 2H), 5.03~5.02 (d, 1H), 4.42~4.37 (q, 1H), 3.72 (s, 3H), 1.68~1.52 (m, 2H), 0.83~0.79 (t, 3H). ^{13}C NMR (101 MHz, DMSO-d6): δ 158.51, 138.62, 127.43, 113.70, 73.80, 55.38, 32.57, 10.58.

1-(3, 4-Dimethoxyphenyl)propan-1-ol (**2a**)

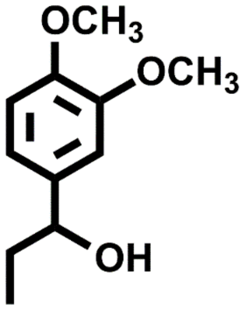

3, 4-Dimethoxybenzaldehyde (1.7 g, 10 mmol) was added into the solution of Grignard reagent, freshly prepared from bromoethane (1.6 g, 15 mmol) and magnesium turnings (0.4 g, 15 mmol) in anhydrous THF (20 mL) at 0 °C. The reaction mixture was stirred at room temperature for 1 h. After the reaction, the mixture was quenched with cold water (1 mL) and acidified with saturated NH_4Cl solution (20 mL). Ethyl acetate (50 mL) was added into the stirred organic layer at room temperature. The organic layer was then successively washed with deionized water (50 mL×3) and saturated brine (50 mL), dried by anhydrous $MgSO_4$. After the concentration in

vacuum rotavap, 1.7 g of **2a** was obtained finally. ^{1}H NMR (400 MHz, DMSO-d6): δ 6.91 (s, 1H), 6.88~6.86 (d, 1H), 6.81~6.79 (d, 1H), 4.99~4.98 (d, 1H), 4.39~4.34 (q, 1H), 3.74 (s, 3H), 3.72 (s, 3H), 1.65~1.53 (m, 2H), 0.83~0.79 (t, 3H). ^{13}C NMR (101 MHz, DMSO-d6): δ 148.99, 148.03, 139.31, 118.36, 111.96, 110.30, 73.96, 55.89, 32.53, 10.64.

4-(1-Hydroxypropyl)-2-methoxyphenol (**3a**)

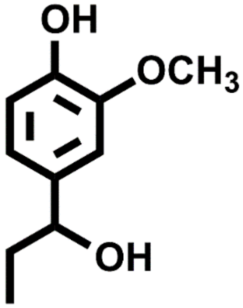

4-Hydroxy-3-methoxybenzaldehyde (1.5 g, 10 mmol) was added into the solution of Grignard reagent that was freshly prepared from bromoethane (3.3 g, 30 mmol) and magnesium turnings (0.7 g, 30 mmol) in anhydrous THF (20 mL) at 0 °C. The reaction mixture was stirred at room temperature for 1 h. After the reaction, the mixture was quenched with cold water (1 mL) and acidified with saturated NH_4Cl solution (20 mL). Ethyl acetate (50 mL) was added into the stirred organic layer at room temperature. The organic layer was then successively washed with deionized water (50 mL×3) and saturated brine (50 mL), dried by anhydrous $MgSO_4$. After the concentration in vacuum rotavap, 0.9 g of **3a** was obtained finally. ^{1}H NMR (400 MHz, DMSO-d6): δ 8.69 (s, 1H), 6.90 (s, 1H), 6.72 (s, 2H), 4.95 (d, 1H), 4.36~4.35 (q, 1H), 3.77 (s, 3H), 1.67~1.55 (m, 2H), 0.85~0.81 (t, 3H). ^{13}C NMR (101 MHz, DMSO-d6): δ 147.72, 145.65, 137.71, 118.77, 115.36, 110.63, 74.18, 56.04, 32.55, 10.70.

1-(4-isopropoxy-3-methoxyphenyl)propan-1-ol (**4a**)

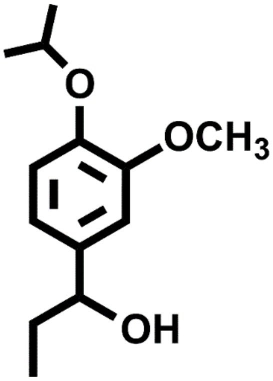

4-Hydroxy-3-methoxybenzaldehyde (3.0 g, 20 mmol) was dissolved in 20 mL DMF at room temperature. 2-Bromopropane (4.9 g, 40 mmol) and anhydrous K_2CO_3 (5.5 g, 40 mmol) were subsequently added and stirred at 100 °C for 6 h. After the reaction, the mixture was poured into water (200 mL) and extracted by ethyl acetate (50 mL×3). The organic layer was then successively washed with saturated brine (20 mL×6), dried by anhydrous $MgSO_4$ and concentrated in vacuum. Finally, 3.5 g of 4-isopropoxy-3-methoxybenzaldehyde was obtained by silica column chromatography.

4-Isopropoxy-3-methoxybenzaldehyde (3.5 g, 18 mmol) was added into the solution of Grignard reagent, freshly prepared from bromoethane (3.2 g, 30 mmol) and magnesium turnings (0.8 g, 30 mmol) in anhydrous THF (50 mL) at 0 °C. The reaction mixture was stirred at room temperature for 1 h. After the reaction, the mixture was quenched with cold water (1 mL) and acidified with saturated NH_4Cl solution (50 mL). Ethyl acetate (100 mL) was added into the stirred organic layer at room temperature. The organic layer was then successively washed with deionized water (50 mL×3) and saturated brine (50 mL), dried by anhydrous $MgSO_4$. After the concentration in vacuum rotavap, 3.9 g of **4a** was obtained finally. ^{1}H NMR (400 MHz, DMSO-d6): δ 6.92 (s, 1H), 6.87~6.85 (d, 1H), 6.79~6.77 (d, 1H), 5.00 (s, 1H), 4.48~4.42 (m, 1H), 4.38~4.35 (m, 1H), 3.74 (s, 3H), 1.60~1.57 (m, 2H), 1.23~1.22 (d, 6H), 0.88~0.80 (t, 3H). ^{13}C NMR (101 MHz, DMSO-d6): δ 148.99, 148.03, 139.31, 118.36, 111.96, 110.30, 73.96, 55.89, 32.53, 10.64. ^{13}C NMR (101 MHz, DMSO-d6): δ 149.85, 145.34, 139.41, 117.88, 115.73, 115.71, 110.27, 73.52, 70.48, 55.40, 32.03, 21.96, 10.20.

1-(3, 4, 5-Trimethoxyphenyl) propan-1-ol (**5a**)

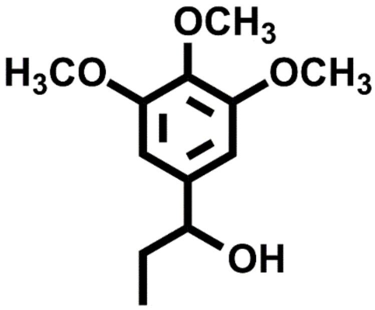

3, 4, 5-Trimethoxybenzaldehyde (2.0 g, 10 mmol) was added into the solution of Grignard reagent that was freshly prepared from bromoethane (1.6 g, 15 mmol) and magnesium turnings (0.4 g, 15 mmol) in anhydrous THF (20 mL) at 0 °C. The reaction mixture was stirred at room temperature for 1 h. After the reaction, the mixture was quenched with cold water (1 mL) and acidified with saturated NH_4Cl solution (20 mL). Ethyl acetate (50 mL) was added into the stirred organic layer at room temperature. The organic layer was then successively washed with deionized water (50 mL×3) and saturated brine (50 mL), dried by anhydrous $MgSO_4$. After the concentration in vacuum rotavap, 2.0 g of **5a** was obtained finally. ^{1}H NMR (400 MHz, DMSO-d6): δ 6.62 (s, 2H), 5.07 (d, 1H), 4.39~4.36 (q, 1H), 3.76 (s, 6H), 3.63 (s, 3H), 1.63~1.56 (m, 2H), 0.86~0.83 (t, 3H). ^{13}C NMR (101 MHz, DMSO-D6): δ 153.01, 142.52, 136.59, 103.52, 74.24, 60.40, 56.24, 32.52, 10.69.

4-(1-Hydroxypropyl)-2, 6-dimethoxyphenol (**6a**)

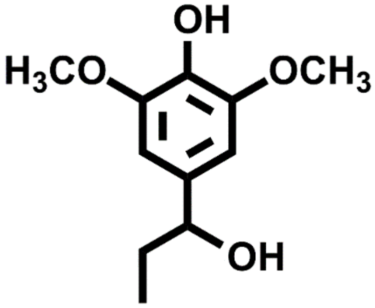

4'-Hydroxy-3', 5'-dimethoxybenzaldehyde (1.8 g, 10 mmol) was added into the solution of Grignard reagent that was freshly prepared from bromoethane (3.3 g, 30 mmol) and magnesium turnings (0.7 g, 30 mmol) in anhydrous THF (20 mL) at 0 °C. The reaction mixture was stirred at room temperature for 1 h. After the reaction, the mixture was quenched with cold water (1 mL) and acidified with saturated NH_4Cl solution (20 mL). Ethyl acetate (50 mL) was added into the stirred organic layer at room temperature. The organic layer was then successively washed with deionized water (50 mL×3) and saturated brine (50 mL), dried by anhydrous $MgSO_4$. After the concentration in vacuum rotavap, 1.3 g of **6a** was obtained finally. ^{1}H NMR (400 MHz, DMSO-d6) δ 8.06 (s, 1H), 6.57 (s, 2H), 4.96 (d, 1H), 4.36~4.32 (q, 1H), 3.76 (s, 6H), 1.63~1.56 (m, 2H), 0.85~0.81 (t, 3H). ^{13}C NMR (101 MHz, DMSO-d6): δ 148.14, 136.91, 134.67, 103.91, 74.38, 56.42, 32.60, 10.71.

1-(4-isopropoxy-3, 5-dimethoxyphenyl)propan-1-ol (**7a**)

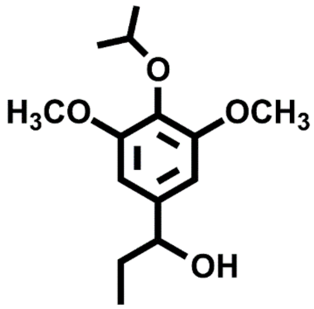

4-Hydroxy-3, 5-dimethoxybenzaldehyde (3.6 g, 20 mmol) was dissolved in 20 mL DMF at room temperature. 2-Bromopropane (4.9 g, 40 mmol) and anhydrous K_2CO_3 (5.5 g, 40 mmol) were subsequently added and stirred at 100 °C for 6 h. After the reaction, the mixture was poured into water (200 mL) and extracted by ethyl acetate (50 mL×3). The organic layer was then successively washed with saturated brine (20 mL×6), dried by anhydrous $MgSO_4$ and concentrated in vacuum. Finally, 4.0 g of 4-isopropoxy-3, 5-dimethoxybenzaldehyde was obtained by silica column chromatography.

4-Isopropoxy-3, 5-dimethoxybenzaldehyde (4.0 g, 18 mmol) was added into the solution of Grignard reagent, freshly prepared from bromoethane (3.2 g, 30 mmol) and magnesium turnings (0.8 g, 30 mmol) in anhydrous THF (50 mL) at 0 °C. The reaction mixture was stirred at room temperature for 1 h. After the reaction, the mixture was quenched with cold water (1 mL) and acidified with saturated NH_4Cl solution (50 mL). Ethyl acetate (100 mL) was added into the stirred organic layer at room temperature. The organic layer was then successively washed with deionized water (50 mL×3) and saturated brine (50 mL), dried by anhydrous $MgSO_4$. After the concentration in vacuum rotavap, 3.9 g of **7a** was obtained finally. ^{1}H NMR (400 MHz, DMSO-d6): δ 6.60 (s, 2H), 5.06~5.05 (d, 1H), 4.39~4.34 (m, 1H), 4.24~4.15 (m, 1H), 3.73 (s, 3H), 1.63~1.56 (m, 2H), 1.16~1.15 (d, 6H), 0.86~0.83 (t, 3H). ^{13}C NMR (101 MHz, DMSO-d6): δ 153.52, 142.11, 134.32, 103.44, 74.31, 74.28, 56.23, 32.51, 22.84, 10.76.

1-(3, 4-dimethoxyphenyl)-2-(2-methoxyphenoxy)propane-1, 3-diol (**8a**)

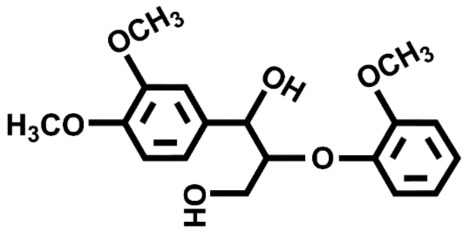

Bromine (33.6g, 210 mmol) was added dropwise into the solution of 3', 4'-dimethoxyacetophenone (36.4 g, 200 mmol) and $AlCl_3$ (667 mg, 5 mmol) in diethyl ether (200 mL) at 0 °C~5 °C. The reaction mixture was then stirred at room temperature for 1h. After the reaction, the mixture was poured into ice water (1000 mL) and extracted by ethyl acetate (200 mL×2). The organic layer was then successively washed with deionized water (200 mL×3) and saturated brine (100 mL), dried by anhydrous $MgSO_4$ and concentrated in vacuum. Finally, 33.7 g of 2-bromo-1-(3, 4-dimethoxyphenyl)ethanone was obtained by recrystallization process.

2-Methoxyphenol (16.4 g, 132 mmol) and K_2CO_3 (33.1 g, 240 mmol) were added into the solution of 2-bromo-1-(3, 4-dimethoxyphenyl)ethanone (31.1 g, 120 mmol) in acetone (200 mL) at room temperature. The reaction mixture was stirred at room temperature for 24 h. After the reaction, the mixture was poured into 500 mL ice water and extracted by ethyl acetate (100 mL×2). The organic layer was then successively washed with deionized water (100 mL×3) and saturated brine (100 mL), dried by anhydrous $MgSO_4$ and concentrated in vacuum. Finally, 32.1 g of 1-(3, 4-dimethoxyphenyl)-2-(2-methoxyphenoxy)ethan-1-one was obtained by recrystallization process.

Formaldehyde solution (37%, 16.2 g, 200 mmol) and anhydrous K_2CO_3 (27.6 g, 200 mmol) were added into a solution of 1-(3, 4-dimethoxyphenyl)-2-(2-methoxyphenoxy)ethan-1-one (30.2 g, 100 mmol) in acetone (200 mL) at room temperature. The reaction mixture was then stirred at room temperature for 1 h. After the reaction, mixture was poured into 500 mL ice water and extracted by ethyl acetate (50 mL×2). The organic layer was then successively washed with deionized water (100 mL×3) and saturated brine (100 mL), dried by anhydrous $MgSO_4$ and concentrated in vacuum. Finally, 27.9 g of 1-(3, 4-dimethoxyphenyl)-3-hydroxy-2-(2-methoxyphenoxy)propan-1-one was obtained by recrystallization process.

Sodium borohydride (3.0 g, 80 mmol) was added into the solution of 1-(3, 4-dimethoxyphenyl)-3-hydroxy-2-(2-methoxyphenoxy)propan-1-one (26.6 g, 80 mmol) in THF/H_2O (100 mL/30 mL) at 0°C. The reaction mixture was stirred at room temperature for 24 h. After the reaction, the mixture was sat for 10 min, and then the organic layer was separated. Ethyl acetate (50 mL) was added into the stirred organic layer at room temperature. The organic layer was then successively washed with deionized water (50 mL×3) and saturated brine (50 mL), dried by anhydrous $MgSO_4$. After the concentration in vacuum rotavap, 24.7 g of **8a** was obtained finally. ^{1}H NMR (400 MHz, DMSO-d6): δ 7.08~6.85 (m, 7H), 5.43~5.38 (q, 1H), 4.83~4.79 (m, 2H), 4.72~ 4.65 (m, 1H), 4.38~ 4.31 (m, 1H), 3.78~3.72 (m, 9H) 3.64~3.26 (m, 2H). ^{13}C NMR (101 MHz, DMSO-d6): δ 149.83, 149.79, 148.37, 148.22, 148.10, 147.88, 147.84, 134.90, 134.59, 121.17, 121.07, 120.75, 120.69, 119.30, 118.73, 115.97, 115.89, 112.68, 112.61, 111.21, 111.18, 111.10, 110.74, 84.34, 83.68, 71.63, 70.87, 60.12, 55.62, 55.52, 55.50, 55.31, 55.29.

2-(2-methoxyphenoxy)-1-(3, 4, 5-trimethoxyphenyl) propane-1, 3-diol (**9a**)

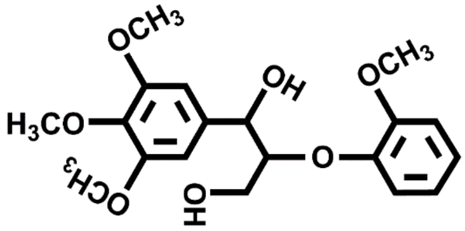

Bromine (33.6 g, 210 mmol) was added dropwise into the solution of 3', 4', 5'-trimethoxyacetophenone (42.0 g, 200 mmol) and $AlCl_3$ (667 mg, 5 mmol) in diethyl ether/tetrahydrofuran (50 mL/150 mL) at 0 °C~5 °C. The reaction mixture was then stirred at room temperature for 1h. After the reaction, the mixture was poured into ice water (1000 mL) and extracted by ethyl acetate (200 mL×2). The organic layer was then successively washed with deionized water (200 mL×3) and saturated brine (100 mL), dried by anhydrous $MgSO_4$ and concentrated in vacuum. Finally, 41.6 g of 2-bromo-1-(3, 4, 5-trimethoxyphenyl)ethanone was obtained by recrystallization process.

2-Methoxyphenol (16.4 g, 132 mmol) and K_2CO_3 (33.1g, 240 mmol) were added into the solution of 2-bromo-1-(3, 4, 5-trimethoxyphenyl)ethanone (34.7 g, 120 mmol) in acetone (200 mL) at room temperature. The reaction mixture was stirred at room temperature for 24 h. After the reaction, the mixture was poured into 500 mL ice water and extracted by ethyl acetate (100 mL×2). The organic layer was then successively washed with deionized water (100 mL×3) and saturated brine (100 mL), dried by anhydrous $MgSO_4$ and concentrated in vacuum. Finally, 32.6 g of 2-(2-methoxy-phenoxy)-1-(3, 4, 5-trimethoxyphenyl)ethanone was obtained by recrystallization process.

Formaldehyde solution (37%, 7.1 g, 88 mmol) and anhydrous K_2CO_3 (12.1 g, 88 mmol) were added into a solution of 2-(2-methoxyphenoxy)-1-(3, 4, 5-trimethoxy-phenyl)ethanone (29.0 g, 80 mmol) in acetone (200 mL) at room temperature. The reaction mixture was then stirred at room temperature for 1h. After the reaction, the mixture was poured into 500 mL ice water and extracted by ethyl acetate (100 mL×2). The organic layer was then successively washed with deionized water (100 mL×3) and saturated brine (100 mL), dried by anhydrous $MgSO_4$ and concentrated under vacuum. Finally, 24.1 g of 3-hydroxy-2-(2-methoxyphenoxy)-1-(3,4,5-trimethoxyphenyl)propan-1-one was obtained by recrystallization process.

Sodium borohydride (1.9 g, 50 mmol) was added into the solution of 3-hydroxy-2-(2-methoxyphenoxy)-1-(3, 4, 5-trimethoxyphenyl)propan-1-one (18.1 g, 50 mmol) in THF/H_2O (100/30 v/v) at 0 °C. The reaction mixture was stirred at room temperature for 24 h. After the reaction, the mixture was sat for 10 min, and then the organic layer was separated. Ethyl acetate (50 mL) was added into the stirred organic layer at room temperature. The organic layer was then successively washed with deionized water (50 mL×3) and saturated brine (50 mL), dried by anhydrous $MgSO_4$. After the concentration in vacuum rotavap, 17.3 g of **9a** was obtained finally. ^{1}H NMR (400 MHz, DMSO-d6): δ 7.05~6.79 (m, 4H), 6.74~6.72 (d, 2H), 5.46~5.42 (q, 1H), 4.81~4.76 (m, 1H), 4.75~4.63 (m, 1H), 4.37~4.33 (m, 1H), 3.76~3.27 (m, 14H). ^{13}C NMR (101 MHz, DMSO-d6): δ 152.78, 152.72, 150.16, 148.65, 148.48, 138.47, 138.14, 136.92, 136.84, 121.58, 121.44, 121.14, 121.06, 116.27, 116.14, 113.02, 112.98, 105.04, 104.42, 84.28, 83.80, 72.40, 71.38, 60.66, 60.53, 60.42, 60.39, 56.16, 56.13, 56.01, 55.98.

1-(4-isopropoxy-3-methoxyphenyl)-2-(2-methoxyphenoxy)propane-1, 3-diol (**10a**)

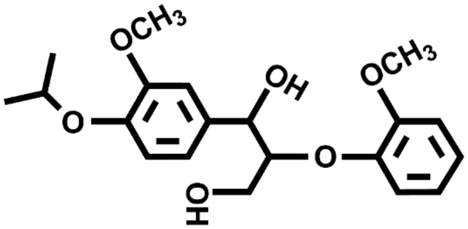

4'-Hydroxy-3'-methoxyacetophenone (33.2 g, 200 mmol) was dissolved in 200 mL DMF at room temperature. 2-Bromopropane (49.2 g, 400 mmol) and anhydrous K_2CO_3 (55.3 g, 400 mmol) were subsequently added and stirred at 100 °C for 6 h. After the reaction, the mixture was poured into water (2000 mL) and extracted by ethyl acetate (250 mL×4). The organic layer was then successively washed with saturated brine (50 mL×10), dried by anhydrous $MgSO_4$ and concentrated in vacuum. Finally, 40.6 g of 1-(4-isopropoxy-3-methoxyphenyl)ethan-1-one was obtained by recrystallization process.

Bromine (32.7 g, 205 mmol) was added dropwise into the solution of 1-(4-isopropoxy-3-methoxyphenyl)ethan-1-one (40.6 g, 195 mmol) and $AlCl_3$ (667 mg, 5 mmol) in diethyl ether (200 mL) at 0 °C~5 °C. The reaction mixture was then stirred at room temperature for 1 h. After the reaction, the mixture was poured into ice water (1000 mL) and extracted by ethyl acetate (200 mL×2). The organic layer was then successively washed with deionized water (200 mL×3) and saturated brine (100 mL), dried by anhydrous $MgSO_4$ and concentrated in vacuum. Finally, 38.1 g of 2-bromo-1-(4-isopropoxy-3-methoxyphenyl)ethan-1-one was obtained by recrystallization process.

2-Methoxyphenol (16.4 g, 132 mmol) and K_2CO_3 (33.1 g, 240 mmol) were added into the solution of 2-bromo-1-(4-isopropoxy-3-methoxyphenyl)ethan-1-one (34.5 g, 120 mmol) in acetone (200 mL) at room temperature. The reaction mixture was stirred at room temperature for 24 h. After the reaction, the mixture was poured into 500 mL ice water and extracted by ethyl acetate (100 mL×2). The organic layer was then successively washed with deionized water (100 mL×3) and saturated brine (100 mL), dried by anhydrous $MgSO_4$ and concentrated in vacuum. Finally, 33.4 g of 1-(4-isopropoxy-3-methoxyphenyl)-2-(2-methoxyphenoxy)ethan-1-one was obtained by recrystallization process.

Formaldehyde solution (37%, 13.0 g, 160 mmol) and anhydrous K_2CO_3 (22.1 g, 160 mmol) were added into a solution of 1-(4-isopropoxy-3-methoxyphenyl)-2-(2-methoxyphenoxy)ethan-1-one (26.4 g, 80 mmol) in acetone (200 mL) at room temperature. The reaction mixture was then stirred at room temperature for 1 h. After the reaction, the mixture was poured into 500 mL ice water and extracted by ethyl acetate (100 mL×2). The organic layer was then successively washed with deionized water (100 mL×3) and saturated brine (100 mL), dried by anhydrous $MgSO_4$ and concentrated under vacuum. Finally, 25.6 g of 3-hydroxy-1-(4-isopropoxy-3-methoxyphenyl)-2-(2-methoxyphenoxy)propan-1-one was obtained by recrystallization process.

Sodium borohydride (1.9 g, 50 mmol) was added into the solution of 3-hydroxy-1-(4-isopropoxy-3-methoxyphenyl)-2-(2-methoxyphenoxy)propan-1-one (18.0 g, 50 mmol) in THF/H_2O (100/30 v/v) at 0 °C. The reaction mixture was stirred at room temperature for 24 h. After the reaction, the mixture was sat for 10 min, and then the organic layer was separated. Ethyl acetate (50 mL) was added into the stirred organic layer at room temperature. The organic layer was then successively washed with deionized water (50 mL×3) and saturated brine (50 mL), dried by anhydrous $MgSO_4$. After the concentration in vacuum rotavap, 17.1 g of **10a** was obtained

finally. ^{1}H NMR (400 MHz, DMSO-d6): δ 7.05~6.78 (m, 7H), 5.38~5.32 (q, 1H), 4.80~4.74 (m, 1H), 4.67~4.60 (m, 1H), 4.49~4.42 (m, 1H), 4.35~4.28 (m, 1H), 3.76~3.69 (m, 6H), 3.64~3.25 (m, 2H), 1.22~1.20 (q, 2H). ^{13}C NMR (101 MHz, DMSO-d6): δ 150.28, 150.25, 150.01, 150.00, 148.85, 148.58, 146.15, 146.11, 135.89, 135.60, 121.61, 121.51, 121.16, 121.10, 119.76, 119.19, 116.52, 116.45, 115.86, 115.78, 113.16, 113.08, 112.20, 111.72, 84.83, 84.22, 72.13, 71.36, 70.89, 60.66, 60.59, 56.07, 55.80, 55.77, 22.42.

1-(4-isopropoxy-3, 5-dimethoxyphenyl)-2-(2-methoxyphenoxy)propane-1, 3-diol (**11a**)

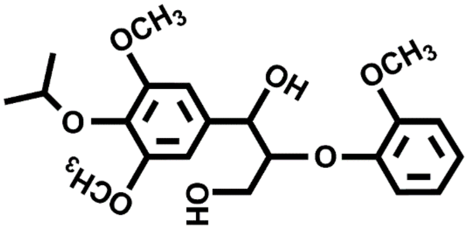

3', 5'-Dimethoxy-4'-hydroxyacetophenone (19.6 g, 100 mmol) was dissolved in 100 mL DMF at room temperature. 2-Bromopropane (24.6 g, 200 mmol) and anhydrous K_2CO_3 (27.7 g, 200 mmol) were subsequently added and stirred at 100 °C for 6 h. After the reaction, the mixture was poured into water (1000 mL) and extracted by ethyl acetate (150 mL×4). The organic layer was then successively washed with saturated brine (25 mL×10), dried by anhydrous $MgSO_4$ and concentrated in vacuum. Finally, 22.9 g of 1-(4-isopropoxy-3, 5-dimethoxyphenyl)ethan-1-one was obtained by recrystallization process.

Bromine (16.0 g, 100 mmol) was added dropwise into the solution of 1-(4-isopropoxy-3, 5-dimethoxyphenyl)ethan-1-one (22.9 g, 96 mmol) and $AlCl_3$ (667 mg, 5 mmol) in diethyl ether (200 mL) at 0 °C ~5 °C. The reaction mixture was then stirred at room temperature for 1 h. After the reaction, the mixture was poured into ice water (1000 mL) and extracted by ethyl acetate (100 mL×3). The organic layer was then successively washed with deionized water (100 mL×3) and saturated brine (100 mL), dried by anhydrous $MgSO_4$ and concentrated in vacuum. Finally, 22.7 g of 2-bromo-1-(4-isopropoxy-3, 5-dimethoxyphenyl)ethan-1-one was obtained by recrystallization process.

2-Methoxyphenol (8.2 g, 66 mmol) and K_2CO_3 (16.6 g, 120 mmol) were added into the solution of 2-bromo-1-(4-isopropoxy-3, 5-dimethoxyphenyl)ethan-1-one (19.0 g, 60 mmol) in acetone (100 mL) at room temperature. The reaction mixture was stirred at room temperature for 24 h. After the reaction, the mixture was poured into 500 mL ice water and extracted by ethyl acetate (50 mL×2). The organic layer was then successively washed with deionized water (50 mL×3) and saturated brine (50 mL), dried by anhydrous $MgSO_4$ and concentrated in vacuum. Finally, 19.8 g of 1-(4-isopropoxy-3, 5-dimethoxyphenyl)-2-(2-methoxyphenoxy)ethan-1-one was obtained by recrystallization process.

Formaldehyde solution (37%, 8.1 g, 100 mmol) and anhydrous K_2CO_3 (13.8 g, 100 mmol) were added into a solution of 1-(4-isopropoxy-3, 5-dimethoxyphenyl)-2-(2-methoxyphenoxy)ethan-1-one (18.0 g, 50 mmol) in acetone (100 mL) at room temperature. The reaction mixture was then stirred at room temperature for 1 h. After the reaction, the mixture was poured into 500 mL ice water and extracted by ethyl acetate (50 mL×2). The organic layer was then successively washed with deionized water (50 mL×3) and saturated brine (50 mL), dried by anhydrous $MgSO_4$ and concentrated under vacuum. Finally, 7.9 g of 3-hydroxy-1-(4-isopropoxy-

3, 5-dimethoxyphenyl)-2-(2-methoxyphenoxy)propan-1-one was obtained by silica column chromatography and recrystallization process.

Sodium borohydride (0.8 g, 20mmol) was added into the solution of 3-hydroxy-1-(4-isopropoxy-3, 5-dimethoxyphenyl)-2-(2-methoxyphenoxy)propan-1-one (7.8 g, 20mmol) in THF/H_2O (100/30 v/v) at 0 °C. The reaction mixture was stirred at room temperature for 24 h. After the reaction, the mixture was sat for 10 min, and then the organic layer was separated. Ethyl acetate (50 mL) was added into the stirred organic layer at room temperature. The organic layer was then successively washed with deionized water (50 mL×3) and saturated brine (50 mL), dried by anhydrous $MgSO_4$. After the concentration in vacuum rotavap, 7.4 g of **11a** was obtained finally. ^{1}H NMR (400 MHz, DMSO-d6): δ 7.00~6.69 (m, 6H), 5.47~5.44 (q, 1H), 4.78~4.65 (m, 2H), 4.39~4.32 (m, 1H), 4.21~4.15 (m, 1H), 3.76~3.68 (m, 9H), 3.68~3.24 (m, 2H), 1.15~1.12 (m, 6H). ^{13}C NMR (101 MHz, DMSO-d6): δ 152.84, 152.76, 149.66, 149.60, 148.21, 148.05, 137.62, 137.29, 134.02, 121.05, 120.87, 120.63, 120.54, 115.61, 112.46, 104.47, 103.81, 83.84, 73.86, 73.79, 70.88, 60.74, 60.30, 60.07, 55.65, 55.52, 55.49, 22.34.

2-(2, 6-dimethoxyphenoxy)-1-(4-isopropoxy-3-methoxyphenyl)propane-1, 3-diol (**12a**)

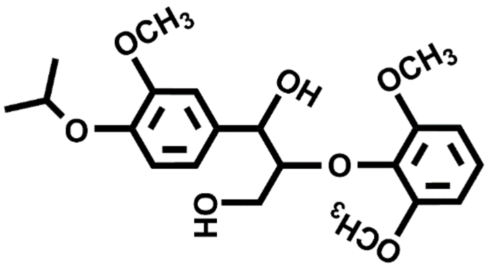

4'-Hydroxy-3'-methoxyacetophenone (33.2 g, 200 mmol) was dissolved in 200 mL DMF at room temperature. 2-Bromopropane (49.2 g, 400 mmol) and anhydrous K_2CO_3 (55.3 g, 400 mmol) were subsequently added and stirred at 100 °C for 6 h. After the reaction, the mixture was poured into water (2000 mL) and extracted by ethyl acetate (250 mL×4). The organic layer was then successively washed with saturated brine (50 mL×10), dried by anhydrous $MgSO_4$ and concentrated in vacuum. Finally, 41.1 g of 1-(4-isopropoxy-3-methoxyphenyl)ethan-1-one was obtained by recrystallization process.

Bromine (32.7 g, 205 mmol) was added dropwise into the solution of 1-(4-isopropoxy-3-methoxyphenyl)ethan-1-one (40.6 g, 195 mmol) and $AlCl_3$ (667 mg, 5 mmol) in diethyl ether (200 mL) at 0 °C~5 °C. The reaction mixture was then stirred at room temperature for 1h. After the reaction, the mixture was poured into ice water (1000 mL) and extracted by ethyl acetate (200 mL×2). The organic layer was then successively washed with deionized water (200 mL×3) and saturated brine (100 mL), dried by anhydrous $MgSO_4$ and concentrated in vacuum. Finally, 40.7 g of 2-bromo-1-(4-isopropoxy-3-methoxyphenyl)ethan-1-one was obtained by recrystallization process.

2, 6-Dimethoxypheno (15.4 g, 132 mmol) and K_2CO_3 (33.1 g, 240 mmol) were added into the solution of 2-bromo-1-(4-isopropoxy-3-methoxyphenyl)ethan-1-one (34.5 g, 120 mmol) in acetone (200 mL) at room temperature. The reaction mixture was stirred at room temperature for 24 h. After the reaction, the mixture was poured into 500 mL ice water and extracted by ethyl acetate (100 mL×2). The organic layer was then successively washed with deionized water (100 mL×3) and saturated brine (100 mL), dried by anhydrous $MgSO_4$ and concentrated in vacuum.

Finally, 40.2 g of 2-(2, 6-dimethoxyphenoxy)-1-(4-isopropoxy-3, 5-dimethoxyphenyl)ethan-1-one was obtained by recrystallization process.

Formaldehyde solution (37%, 13.0 g, 160 mmol) and anhydrous K_2CO_3 (22.1 g, 160 mmol) were added into a solution of 2-(2, 6-dimethoxyphenoxy)-1-(4-isopropoxy-3, 5-dimethoxyphenyl)ethan-1-one (31.2 g, 80 mmol) in acetone (200 mL) at room temperature. The reaction mixture was then stirred at room temperature for 1 h. After the reaction, the mixture was poured into 500 mL ice water and extracted by ethyl acetate (100 mL×2). The organic layer was then successively washed with deionized water (100 mL×3) and saturated brine (100 mL), dried by anhydrous $MgSO_4$ and concentrated under vacuum. Finally, 19.3 g of 2-(2, 6-dimethoxyphenoxy)-3-hydroxy-1-(4-isopropoxy-3, 5-dimethoxyphenyl)propan-1-one was obtained by silica column chromatography and recrystallization process.

Sodium borohydride (1.5 g, 40 mmol) was added into the solution of 2-(2, 6-dimethoxyphenoxy)-3-hydroxy-1-(4-isopropoxy-3, 5-dimethoxyphenyl)propan-1-one (16.8 g, 40 mmol) in THF/H_2O (50 mL/15 mL) at 0 °C. The reaction mixture was stirred at room temperature for 24 h. After the reaction, the mixture was stabilized for 10 min, and then the organic layer was separated. Ethyl acetate (25 mL) was added into the stirred organic layer at room temperature. The organic layer was then successively washed with deionized water (25 mL×3) and saturated brine (25 mL), dried by anhydrous $MgSO_4$. After the concentration in vacuum rotavap, 15.9 g of **12a** was obtained finally. ^{1}H NMR (400 MHz, DMSO-d6): δ 7.04~6.86 (m, 4H), 6.67~6.64 (m, 2H), 5.20~4.94 (m, 1H), 4.92~4.83 (m, 1H), 4.50~4.44 (m, 1H), 4.34~4.13 (m, 1H), 4.08~3.90 (m, 1H), 3.75~3.72 (m, 9H) , 3.69~3.21 (m, 2H). ^{13}C NMR (101 MHz, DMSO-d6): δ 152.80, 149.40, 145.44, 136.17, 135.28, 123.35, 118.81, 115.39, 111.12, 105.72, 105.67, 86.69, 71.24, 70.42, 60.10, 55.91, 55.89, 55.39, 21.97.

2-(2, 6-dimethoxyphenoxy)-1-(4-isopropoxy-3, 5-dimethoxyphenyl)propane-1, 3-diol (**13a**)

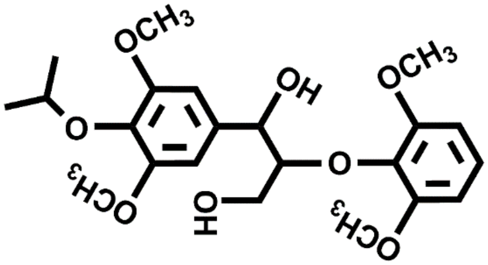

3', 5'-Dimethoxy-4'-hydroxyacetophenone (19.6 g, 100 mmol) was dissolved in 100 mL DMF at room temperature. 2-Bromopropane (24.6 g, 200 mmol) and anhydrous K_2CO_3 (27.7 g, 200 mmol) were subsequently added and stirred at 100 °C for 6 h. After the reaction, the mixture was poured into water (1000 mL) and extracted by ethyl acetate (150 mL×4). The organic layer was then successively washed with saturated brine (25 mL×10), dried by anhydrous $MgSO_4$ and concentrated in vacuum. Finally, 22.8 g of 1-(4-isopropoxy-3, 5-dimethoxyphenyl)ethan-1-one was obtained by recrystallization process.

Bromine (16.0 g, 100 mmol) was added dropwise into the solution of 1-(4-isopropoxy-3, 5-dimethoxyphenyl)ethan-1-one (22.8 g, 96 mmol) and $AlCl_3$ (667 mg, 5 mmol) in diethyl ether (200 mL) at 0 °C~5 °C. The reaction mixture was then stirred at room temperature for 1 h. After the reaction, the mixture was poured into ice water (1000 mL) and extracted by ethyl acetate (100 mL×3). The organic layer was then successively washed with deionized water (100 mL×3) and saturated brine (100 mL), dried by anhydrous $MgSO_4$ and concentrated in vacuum. Finally, 22.4

g of 2-bromo-1-(4-isopropoxy-3, 5-dimethoxyphenyl)ethan-1-one was obtained by recrystallization process.

2, 6-Dimethoxypheno (11.9 g, 77 mmol) and K_2CO_3 (19.3 g, 140 mmol) were added into the solution of 2-bromo-1-(4-isopropoxy-3-methoxyphenyl)ethan-1-one (22.2 g, 70 mmol) in acetone (100 mL) at room temperature. The reaction mixture was stirred at room temperature for 24 h. After the reaction, the mixture was poured into 500 mL ice water and extracted by ethyl acetate (500 mL×3). The organic layer was then successively washed with deionized water (50 mL×3) and saturated brine (50 mL), dried by anhydrous $MgSO_4$ and concentrated in vacuum. Finally, 26.1 g of 2-(2, 6-dimethoxyphenoxy)-1-(4-isopropoxy-3, 5-dimethoxyphenyl)ethan-1-one was obtained by recrystallization process.

Formaldehyde solution (37%, 9.7 g, 120 mmol) and anhydrous K_2CO_3 (16.6 g, 120 mmol) were added into a solution of 2-(2, 6-dimethoxyphenoxy)-1-(4-isopropoxy-3, 5-dimethoxyphenyl)ethan-1-one (23.4 g, 60 mol) in acetone (100 mL) at room temperature. The reaction mixture was then stirred at room temperature for 1 h. After the reaction, the mixture was poured into 500 mL ice water and extracted by ethyl acetate (50 mL×3). The organic layer was then successively washed with deionized water (5 mL×3) and saturated brine (50 mL), dried by anhydrous $MgSO_4$ and concentrated under vacuum. Finally, 19.3 g of 2-(2, 6-dimethoxyphenoxy)-3-hydroxy-1-(4-isopropoxy-3, 5-dimethoxyphenyl)propan-1-one was obtained by silica column chromatography and recrystallization process.

Sodium borohydride (1.5 g, 40 mmol) was added into the solution of 2-(2, 6-dimethoxyphenoxy)-3-hydroxy-1-(4-isopropoxy-3, 5-dimethoxyphenyl)propan-1-one (16.8 g, 40 mmol) in THF/H_2O (50 mL/15 mL) at 0 °C. The reaction mixture was stirred at room temperature for 24 h. After the reaction, the mixture was sat for 10 min, and then the organic layer was separated. Ethyl acetate (25 mL) was added into the stirred organic layer at room temperature. The organic layer was then successively washed with deionized water (25 mL×3) and saturated brine (25 mL), dried by anhydrous $MgSO_4$. After the concentration in vacuum rotavap, 15.9 g of **13a** was obtained finally. ^{1}H NMR (400 MHz, DMSO-d6): δ 6.99~6.93 (m, 1H), 6.72~6.62 (m, 4H), 5.28~4.96 (m, 1H), 4.90~4.86 (m, 1H), 4.43~4.40 (m, 1H), 4.24~4.14 (m, 2H), 3.77~3.72 (m, 12H), 3.71~3.28 (m, 2H). ^{13}C NMR (101 MHz, DMSO-d6): δ 152.81, 152.78, 152.71, 137.62, 136.03, 133.92, 123.34, 105.63, 103.71, 86.17, 73.77, 71.26, 60.14, 55.90, 55.87, 55.69, 22.34.

2-(4-Methoxyphenyl)propan-2-ol

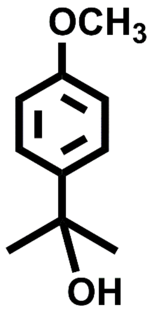

Methylmagnesium chloride solution (3.0 M in THF, 10 mL) was added into the solution of 4'-methoxyacetophenone (1.4 g, 10 mmol) in THF (20 mL) at 0 °C. The reaction mixture was stirred at room temperature for 1 h. After the reaction, the mixture was quenched with cold water (1 mL) and acidified with saturated NH_4Cl solution (20 mL). Ethyl acetate (50 mL) was added into the stirred organic layer at room temperature. The organic layer was then successively washed

with deionized water (50 mL×3) and saturated brine (50 mL), dried by anhydrous $MgSO_4$. After the concentration in vacuum rotavap, 1.5 g of 2-(4-Methoxyphenyl)propan-2-ol was obtained finally. ^1H NMR (400 MHz, DMSO-d6): δ 7.39~7.36 (d, 2H), 6.86~6.84 (d, 2H), 4.93 (s, 1H), 3.72 (s, 6H), 1.41 (s, 3H).^{13}C NMR (101 MHz, DMSO-d6): δ 157.92, 143.12, 126.07, 113.45, 70.75, 55.41, 32.53.

1-methoxy-4-propylbenzene

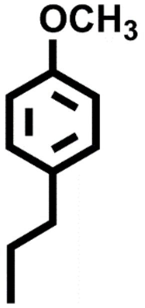

Dimethylsulfate (3.15 g, 25 mmol) and K_2CO_3 (4.1 g, 30 mmol) were added into the solution of 4-propylphenol (1.4 g, 10 mmol) in acetone (50 mL) at room temperature. The reaction mixture was stirred at room temperature for 24 h. After the reaction, the mixture was poured into 200 mL ice water and extracted by ethyl acetate (30 mL×3). The organic layer was then successively washed with saturated Na_2CO_3 solution (30 ml×3) and saturated brine (30 mL), dried by anhydrous $MgSO_4$ and concentrated in vacuum. Finally, 3.1 g of 1-methoxy-4-propylbenzene was obtained. ^1H NMR (400 MHz, DMSO-d6): δ 7.10~7.08 (d, 2H), 6.85~6.83 (d, 2H), 3.72 (s, 3H), 2.50~2.46 (q, 2H), 1.59~1.50 (m, 2H), 0.89~0.86 (t, 3H). ^{13}C NMR (101 MHz, DMSO-d6): δ 157.32, 133.90, 129.11, 113.54, 54.84, 36.36, 24.30, 13.47.

4-(1-Hydroxypropyl)phenol

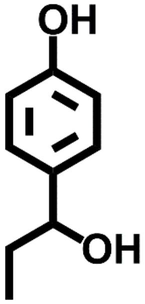

4-Hydroxybenzaldehyde (1.2 g, 10 mmol) was added into the solution of Grignard reagent that was freshly prepared from bromoethane (3.3 g, 30 mmol) and magnesium turnings (0.7 g, 30 mmol) in anhydrous THF (20 mL) at 0 °C. The reaction mixture was stirred at room temperature for 1 h. After the reaction, the mixture was quenched with cold water (1 mL) and acidified with saturated NH_4Cl solution (20 mL). Ethyl acetate (50 mL) was added into the stirred organic layer at room temperature. The organic layer was then successively washed with deionized water (50 mL×3) and saturated brine (50 mL), dried by anhydrous $MgSO_4$. After the concentration in vacuum rotavap, 0.5 g of 4-(1-Hydroxypropyl)phenol was obtained finally. ^1H NMR (400 MHz, DMSO-d6): δ 9.18 (s, 1H), 7.09~7.07 (d, 2H), 6.69~6.67 (d, 2H), 4.90~4.89 (d, 1H), 4.34~4.28 (q, 1H), 1.64~1.47 (m, 2H), 0.80~0.76 (t, 3H). ^{13}C NMR (101 MHz, DMSO-d6): δ 156.46, 136.87, 127.41, 115.06, 73.91, 32.51, 10.69.

Computational methodology

The Vienna Ab Initio Simulation Package (VASP) code based on frozen-coreall-electron projector augmented wave method[3] and plane-wave basis was used to perform the spin-polarized DFT computations. The DFT functional PBE-D3 (Perdew, Burke and Ernzerhof functional with the latest dispersion correction) was used. It corresponds to the PBE functional and the correction for dispersion effects proposed by Grimme *et al*. (DFT-D3)[4]. A converged kinetic energy cutoff of 400 eV was chosen. The structure optimization was carried out by minimizing forces with the conjugate-gradient algorithm until the force on each ion is below 0.03 eV/A and the convergence criteria for electronic self-consistent interactions are 10^{-5}. The zero vibrational energy correction was not taken into account. The minimum energy pathway for elementary reaction steps was performed using climbing-image NEB (Nudged Elastic Band) calculation[5]. A low-symmetry rhombohedral unit cell was used to model faujasite zeolite (a = b = c = 17.21 Å, α = β = γ = 60°). The general formula of a faujasite zeolite with a pure silicon component is $Si_{48}O_{96}$. The faujasite zeolite framework contains only one crystallographically tetrahedral T site. In view of our experimental results, HY zeolite with a Si/Al ratio of 30 showed the best catalytic performance, indicating that there are approximately two Al atoms in a unit cell. Therefore, two Al atoms were included in our HY unit cell model. Moreover, it is reasonable to assume that these two Al atoms in the unit cell are spatially adjacent, i.e., one is the active site, and the other, due to space constraints, can adsorb or desorb water molecules to promote the reaction. The reaction (ΔE) and activation energies (Ea) were calculated using the following two formulas: ΔE = E(FS) − E(IS) and Ea = E(TS) − E(IS), respectively. Here, E(TS), E(IS), and E(FS) are the calculated energies of the transition, initial and final states of each elementary step, respectively. The contribution of entropy to free energy in the adsorption/desorption processes was considered in our computational work. Besora *et al*.[6] reported that the free-energy corrections from the direct application of popular computational packages with its usual simplified models based on IGRRHO (harmonic oscillator, rigid rotor, and particle in-a-box) approaches can give a good estimate of the free-energy changes in the liquid-phase reaction. Greeley and co-workers' work[7] indicated that the most important contributions arise from the translational entropy in the adsorption/desorption process.

Supplementary Notes

Supplementary Note 1

Traditional Route for benzene production from fossil resources

Currently, industrial production of benzene is highly dependent on petroleum and coal resources[8, 9].

Worldwide, around 30% of commercial benzene is produced via catalytic reforming process, in which aromatic molecules are produced from the dehydrogenation of cycloparaffins, dehydroisomerization of alkyl cyclopentanes, and cyclization/dehydrogenation of paraffins. The feed to the catalytic reformer may be a straight-run, hydrocracked, or thermally cracked naphtha fraction in the range of C6 to 200 °C. Generally, a narrow naphtha cut of 71-104 °C is fed to the reformer for the production of desired benzene product. Supported platinum-rhenium on a high surface area alumina support is the most frequently reforming catalyst. In the reforming process, the operating conditions and type of feedstock determine, to a large extent, the amount of produced benzene. In addition, the benzene product is usually recovered from reformate by solvent extraction techniques.

Benzene can be produced from the hydrodemethylation of toluene under catalytic (Hydeal and DETOL processes) or thermal (HDA and THD processes) conditions, which contribute 25-30% of the global benzene supply. During the catalytic hydrodemethylation, toluene is mixed with hydrogen and passed through a vessel loaded with catalysts, such as supported chromium or molybdenum oxides, platinum oxides, on silica or alumina. The operating temperatures range from 500 to 595 °C and pressures are usually 4-6 MPa. The reaction is highly exothermic and the temperature is controlled by the injection of quench hydrogen at several places along with the reaction.

Benzene can also be obtained from pyrolysis gasoline which is yielded as a liquid by-product during the steam cracking process of heavy naphthas or light hydrocarbons, such as propane or butane, to ethylene. Approximately, 30-35% of global benzene production is derived from pyrolysis gasoline. The typical pyrolysis gasoline contains up to 65% aromatics, and about 50% of which is benzene. The remainder of the product is composed of mono and diolefins, which can be removed by a mild hydrogenation step. Alternatively, pure benzene can also be recovered from the pyrolysis gasoline by solvent extraction and subsequent distillation.

Disproportionation of toluene is another route for industrial benzene production, in which two molecules of toluene can be converted into one molecule of benzene and one molecule of mixed-xylene isomers. In industry, economic feasibility of this process is highly dependent on the relative prices of benzene, toluene and xylene, as well as the excess of toluene and strong demand for benzene. In recent years, the prices of xylene and benzene have generally been higher than that of toluene, which presently makes the disproportionation process an attractive alternative to hydrodealkylation.

The industrial supply of benzene can be achieved by high-temperature carbonization of coal in coke ovens. Carbonization processes produce coke or char as the main product, and tar, light oil, gas and aqueous liquor as the by-products. The light oils contain benzene and its homologs in significant concentration. Besides, the light oils produced in the partial gasification processes can supply benzene and its homologs in considerable concentrations. Pyrolysis process of coal which falls under partial liquefaction, produces more oil than carbonization processes, and the oil can

subsequently be converted into benzene and its homologs. Complete liquefaction processes are further divided into hydrogenation and solvent extraction processes, and the benzene product can also be obtained from these two categories.

Supplementary Figures

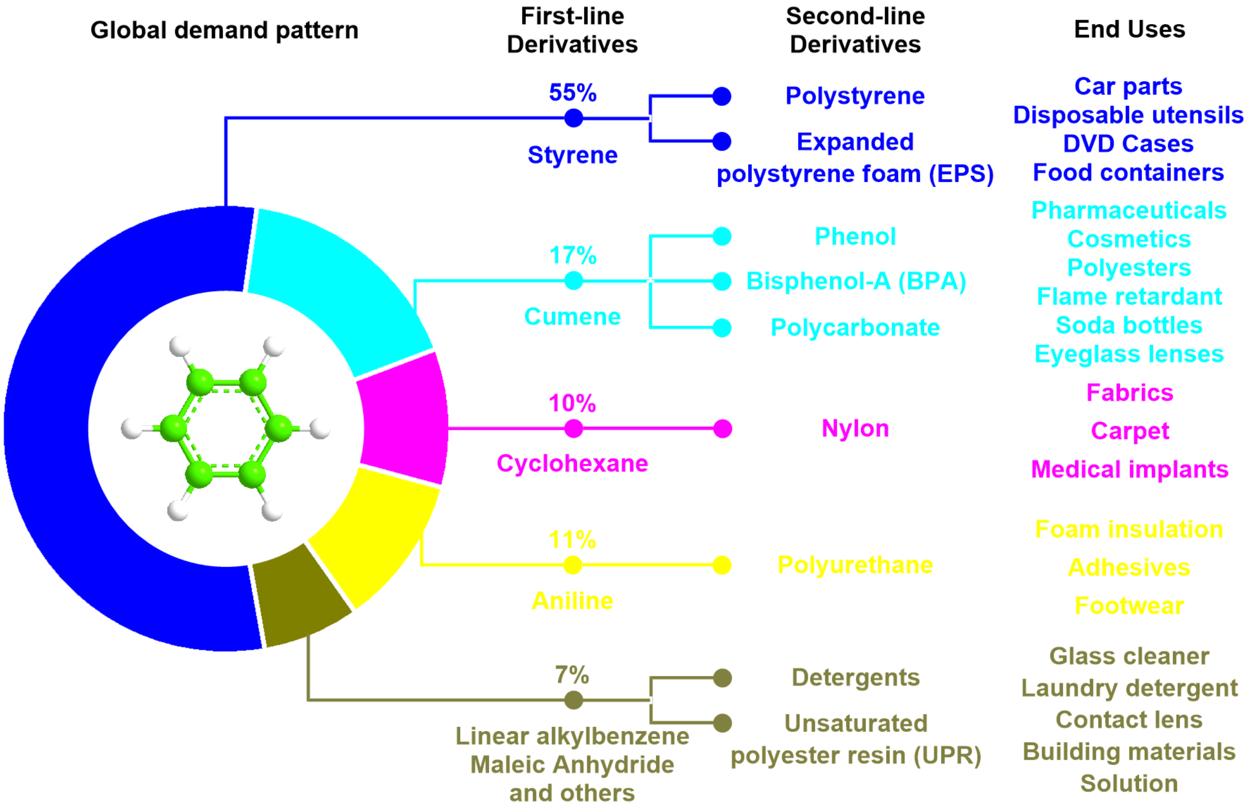

Supplementary Figure 1. Wide applications of benzene in the manufacturing industry.

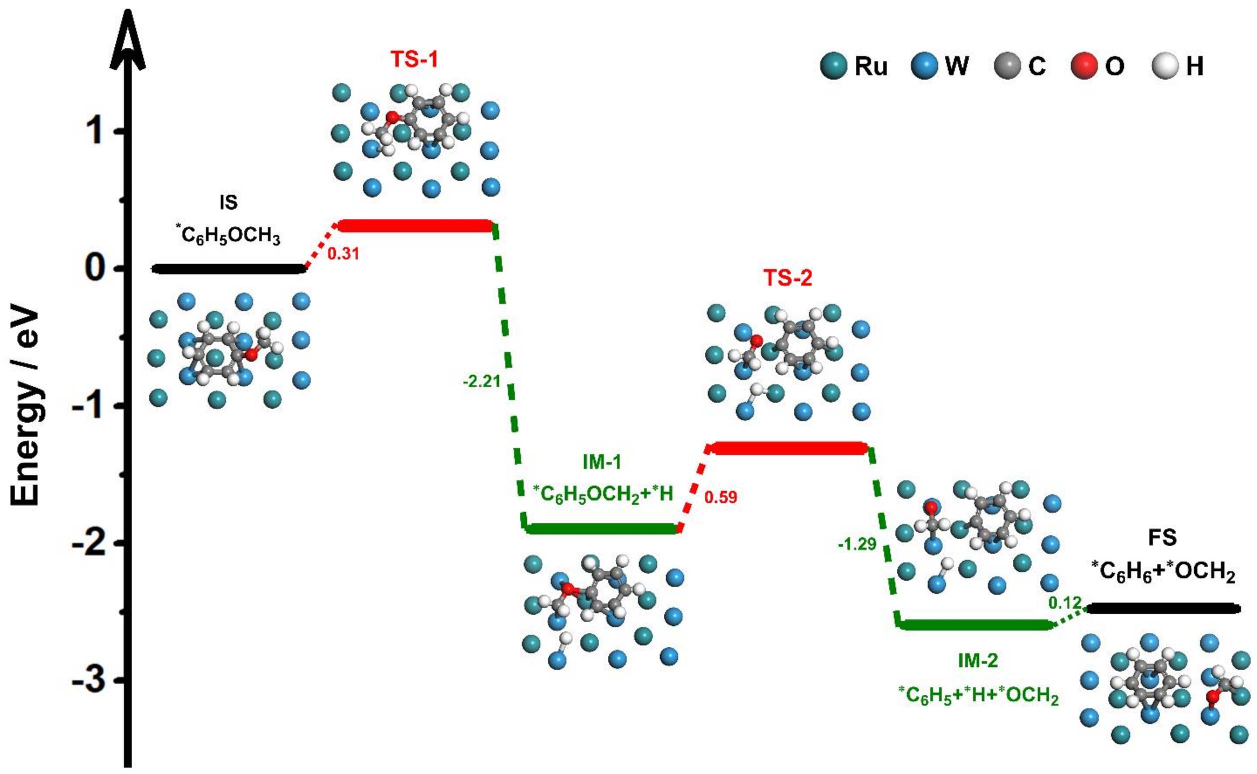

Supplementary Figure 2. Self-supported hydrogenolysis (SSH) of the C_{Ar}-O bond into C_{Ar}-H bond.

(Reference 45 in the main text).

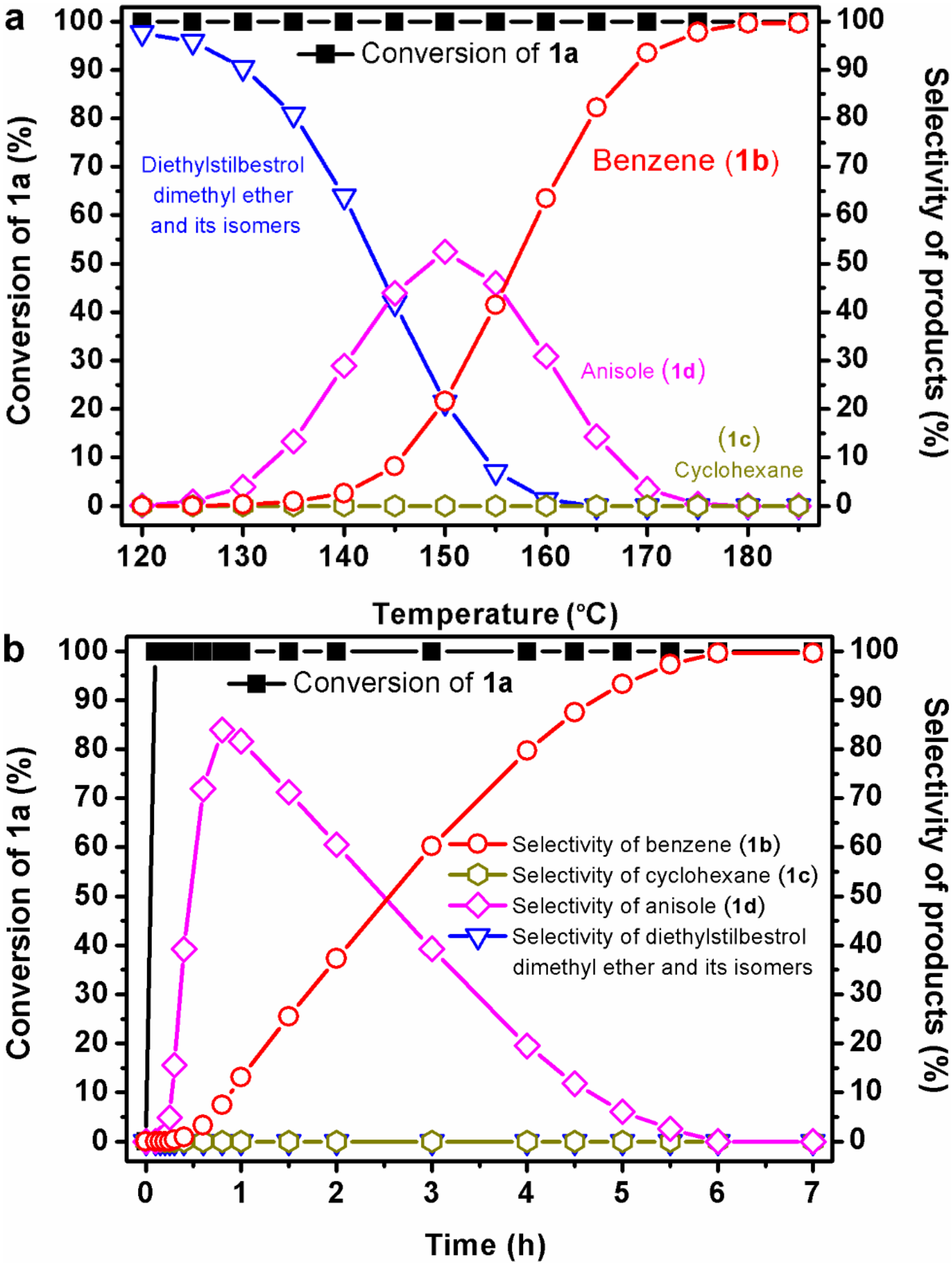
a
Conversion of 1a
Diethylstilbestrol dimethyl ether and its isomers
Benzene (1b)
Anisole (1d)
(1c) Cyclohexane
Conversion of 1a (%)
Selectivity of products (%)
Temperature (°C)
b
Conversion of 1a
Selectivity of benzene (1b)
Selectivity of cyclohexane (1c)
Selectivity of anisole (1d)
Selectivity of diethylstilbestrol dimethyl ether and its isomers
Conversion of 1a (%)
Selectivity of products (%)
Time (h)

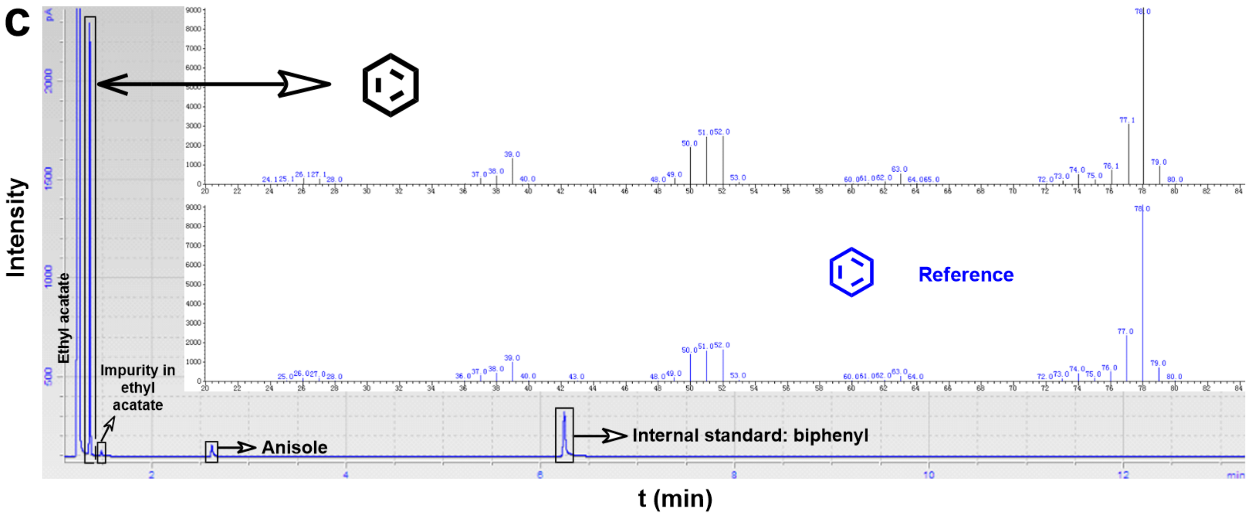
c
Intensity
Ethyl acatate
Impurity in ethyl acatate
Anisole
Internal standard: biphenyl
Reference
t (min)

d

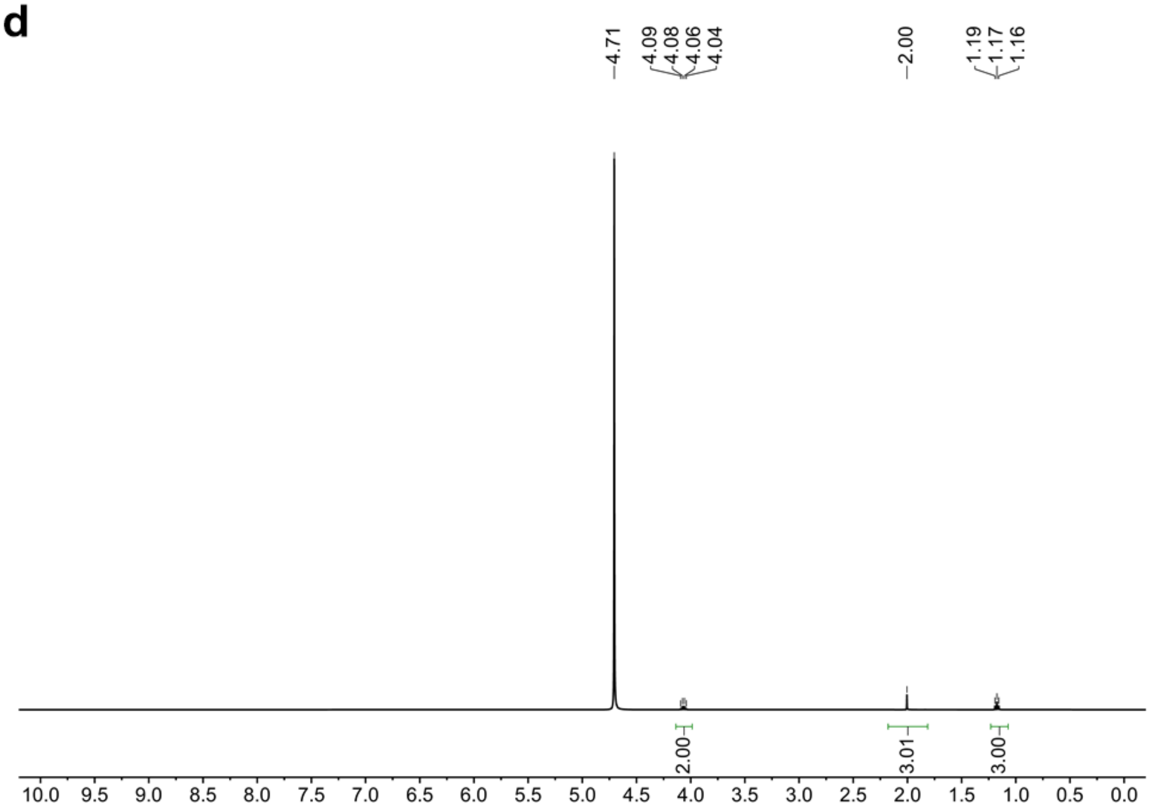

Supplementary Figure 3. The optimization of reaction conditions and analysis of the products obtained from the transformation of 1a over the RuW/HY_{30} catalyst. Effects of reaction temperature (**a**) and reaction time (**b**) on the conversion and product distributions of the reaction. **c** GC trace of the liquid products in ethyl acetate (Inset: MS analysis of benzene). **d** ^{1}H NMR analysis of the product in aqueous phase.

Reaction conditions: **1a** (1.0 mmol), RuW/HY_{30} (0.20 g, 2.5 wt% Ru, 14 wt% W), H_2O (5.0 mL), 0.1 MPa N_2, 800 rpm. **a** 6.0 h. **b** 180 ºC. **c**, **d** 180 ºC, 5.5 h (Main text, Table 1, entry 3).
^{1}H chemical shifts were referenced to D_2O at 4.71 ppm. Ethyl acetate ^{1}H NMR: δ 4.09~4.04 (q, 2H), 2.00 (s, 3H), 1.19~1.16 (t, 3H).

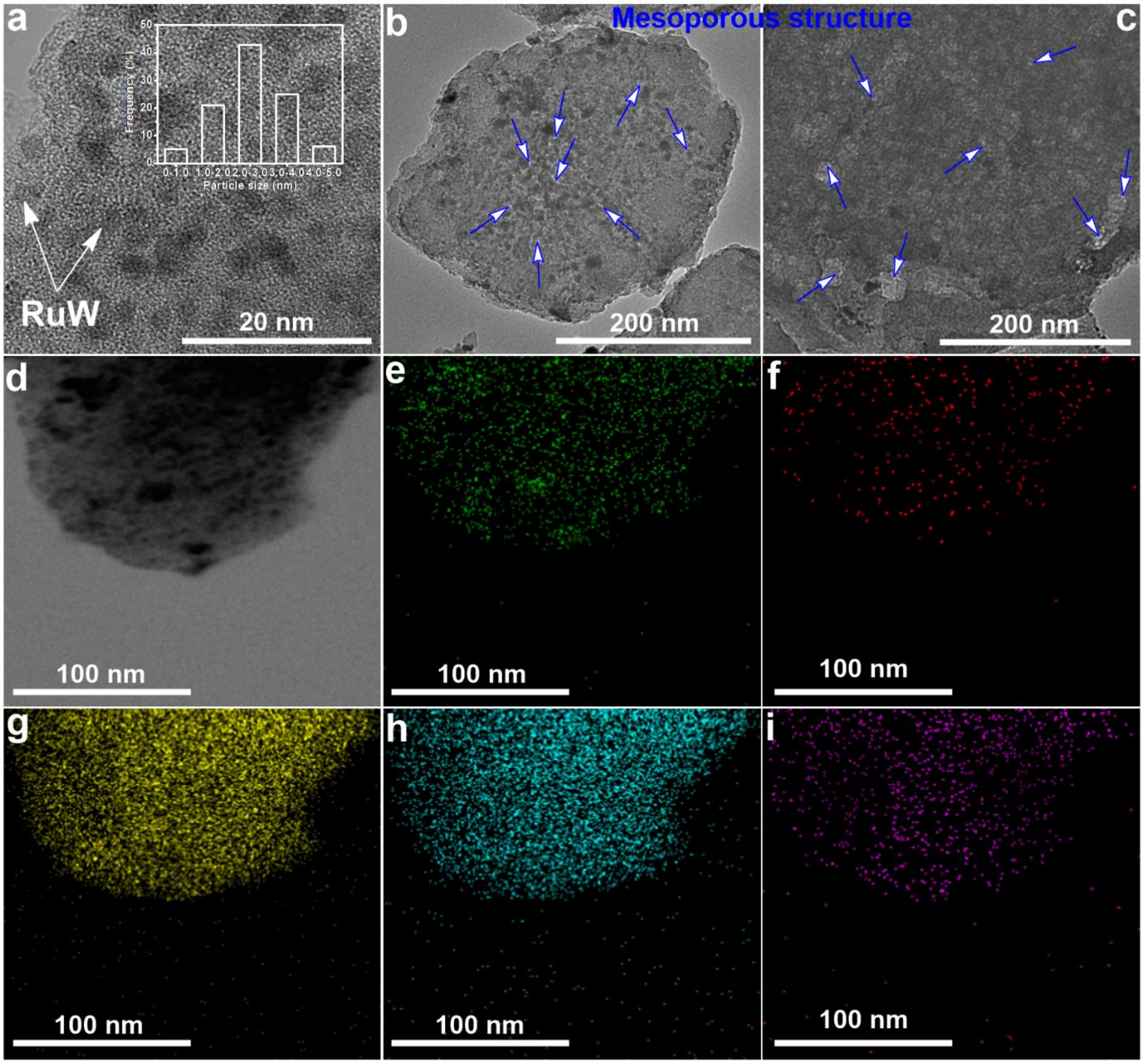

Supplementary Figure 4. TEM analysis of the RuW/HY30 catalyst. a TEM image of RuW/HY30 catalyst (2.5 wt% Ru, 14 wt% W, inset: RuW alloy particle size distribution of RuW/HY30 catalyst). **b**, **c** Mesoporous structures in the RuW/HY30 catalyst. **d** TEM image of RuW/HY30 catalyst for elemental mapping. **e** Elemental mapping of W (green) from the TEM image (**d**). **f** Elemental mapping of Ru (red) from the TEM image (**d**). **g** Elemental mapping of Si (yellow) from the TEM image (**d**). **h** Elemental mapping of O (cyan) from the TEM image (**d**). **i** Elemental mapping of Al (purple) from the TEM image (**d**).

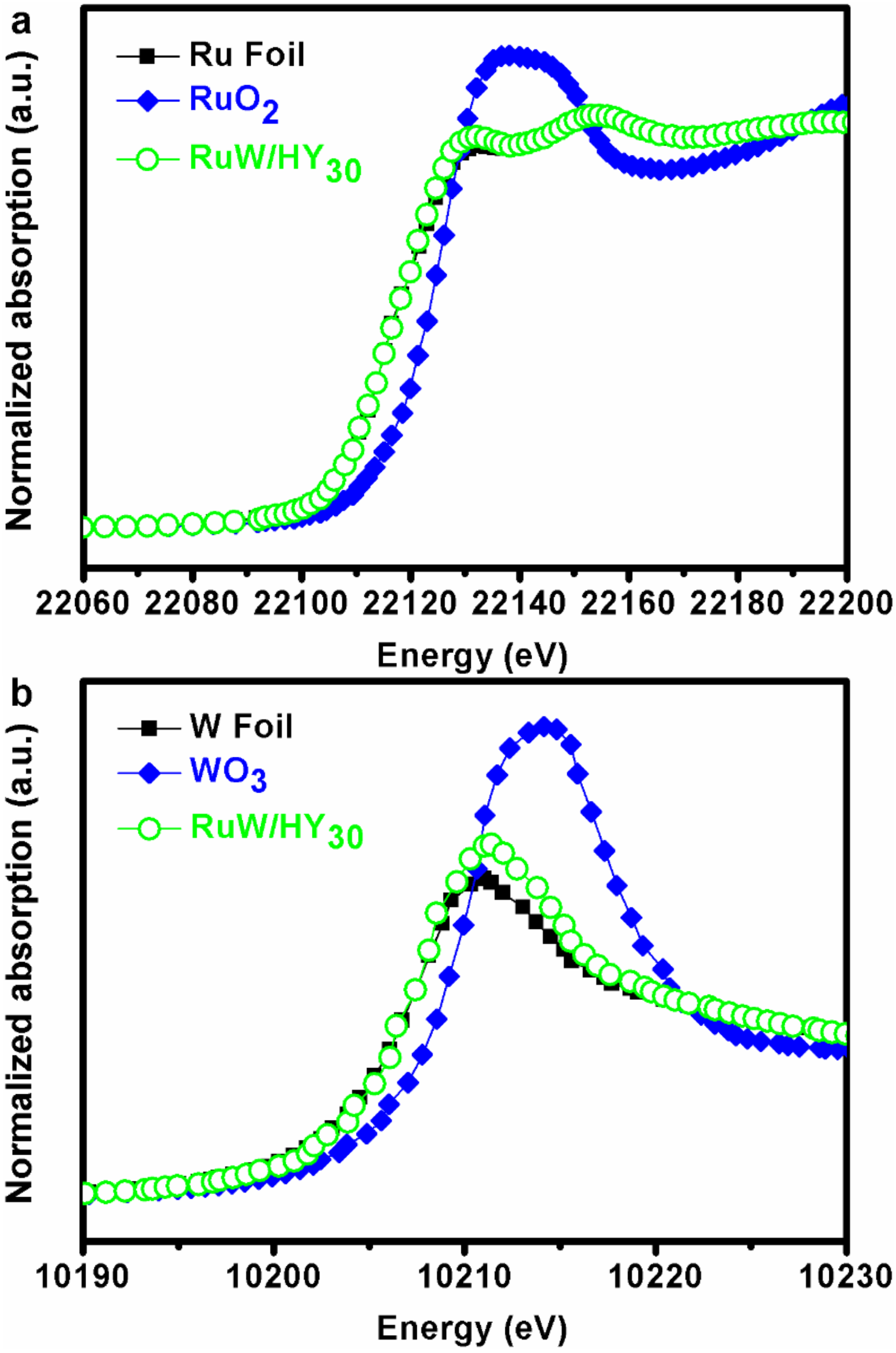

Supplementary Figure 5. X-ray absorption near-edge structure (XANES) spectra of the RuW/HY30 catalyst. a XANES spectra at the Ru *K*-edge of Ru foil, RuO_2 and RuW/HY_{30} (2.5 wt% Ru, 14 wt% W). **b** XANES spectra at the W L_3-edge of W Foil, WO_3 and RuW/HY_{30} (2.5 wt% Ru, 14 wt% W).

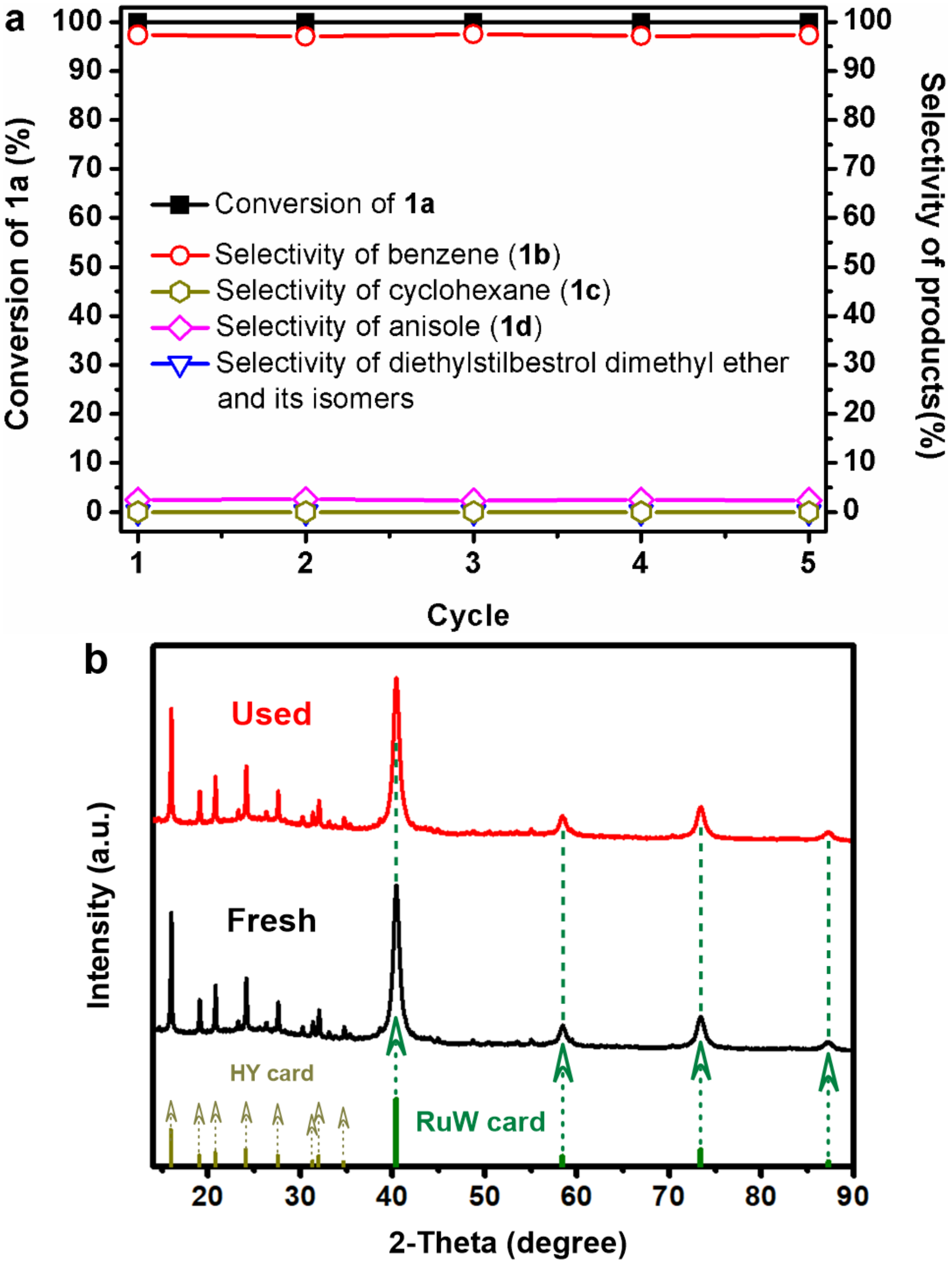
a
b
Conversion of 1a (%)
Selectivity of products(%)
Cycle
Conversion of 1a
Selectivity of benzene (1b)
Selectivity of cyclohexane (1c)
Selectivity of anisole (1d)
Selectivity of diethylstilbestrol dimethyl ether and its isomers
Used
Fresh
HY card
RuW card
Intensity (a.u.)
2-Theta (degree)

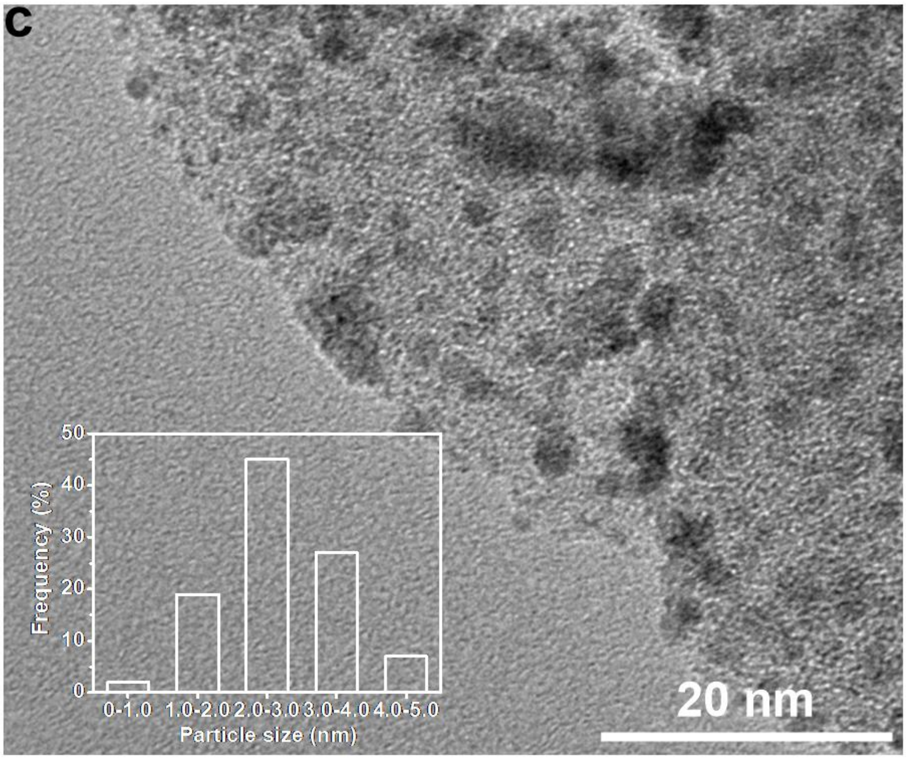

Supplementary Figure 6. Recycling of the RuW/HY$_{30}$ catalyst. a Recyclability of the RuW/HY$_{30}$ catalyst in the reaction of **1a**. **b** XRD patterns of the fresh and used (5 cycles) RuW/HY$_{30}$ catalyst. **c** TEM image and RuW particle size distribution of the used (5 cycles) RuW/HY$_{30}$ catalysts.

Reaction conditions: **1a** (1.0 mmol), RuW/HY$_{30}$ (0.20 g, 2.5 wt% Ru, 14 wt% W, initial dosage), H_2O (5.0 mL), 180 ºC, 5.5 h, 0.1 MPa N_2, 800 rpm.

a

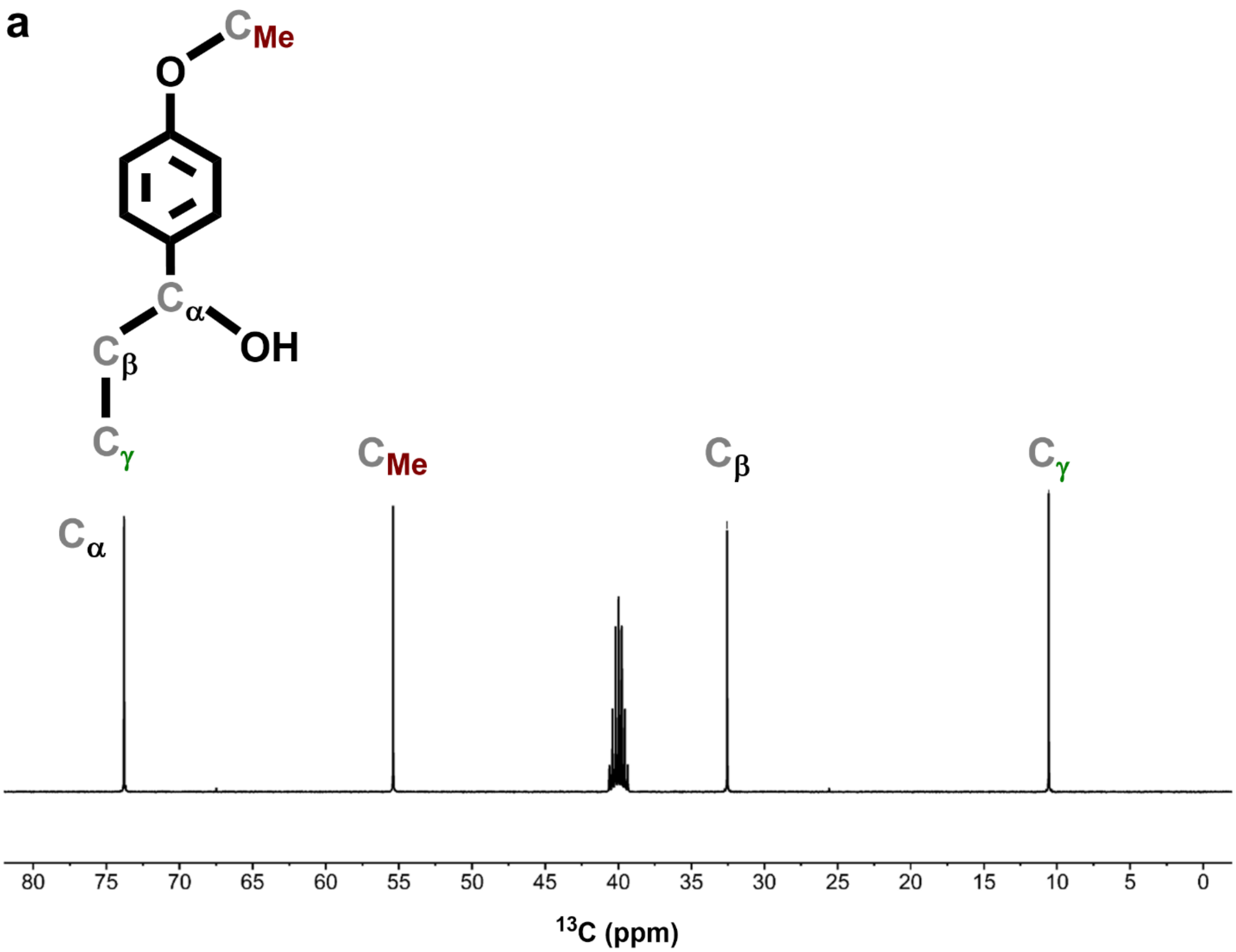
O
CMe
Cα
OH
Cβ
Cγ
CMe
Cβ
Cγ
Cα
80
75
70
65
60
55
50
45
40
35
30
25
20
15
10
5
0
13C (ppm)

b

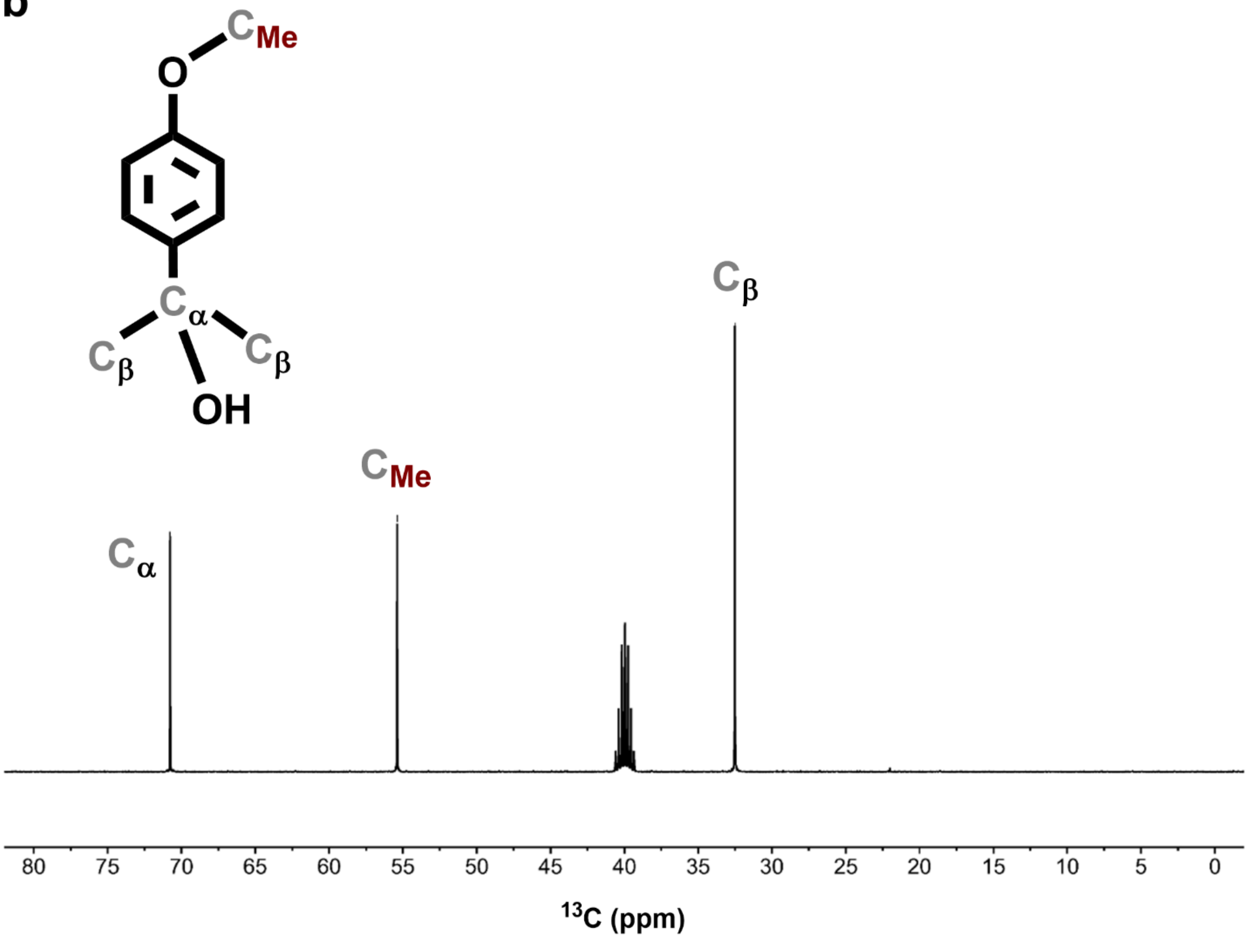

C_Me
O
C_α
C_β
C_β
OH
C_β
C_Me
C_α
80
75
70
65
60
55
50
45
40
35
30
25
20
15
10
5
0
13C (ppm)

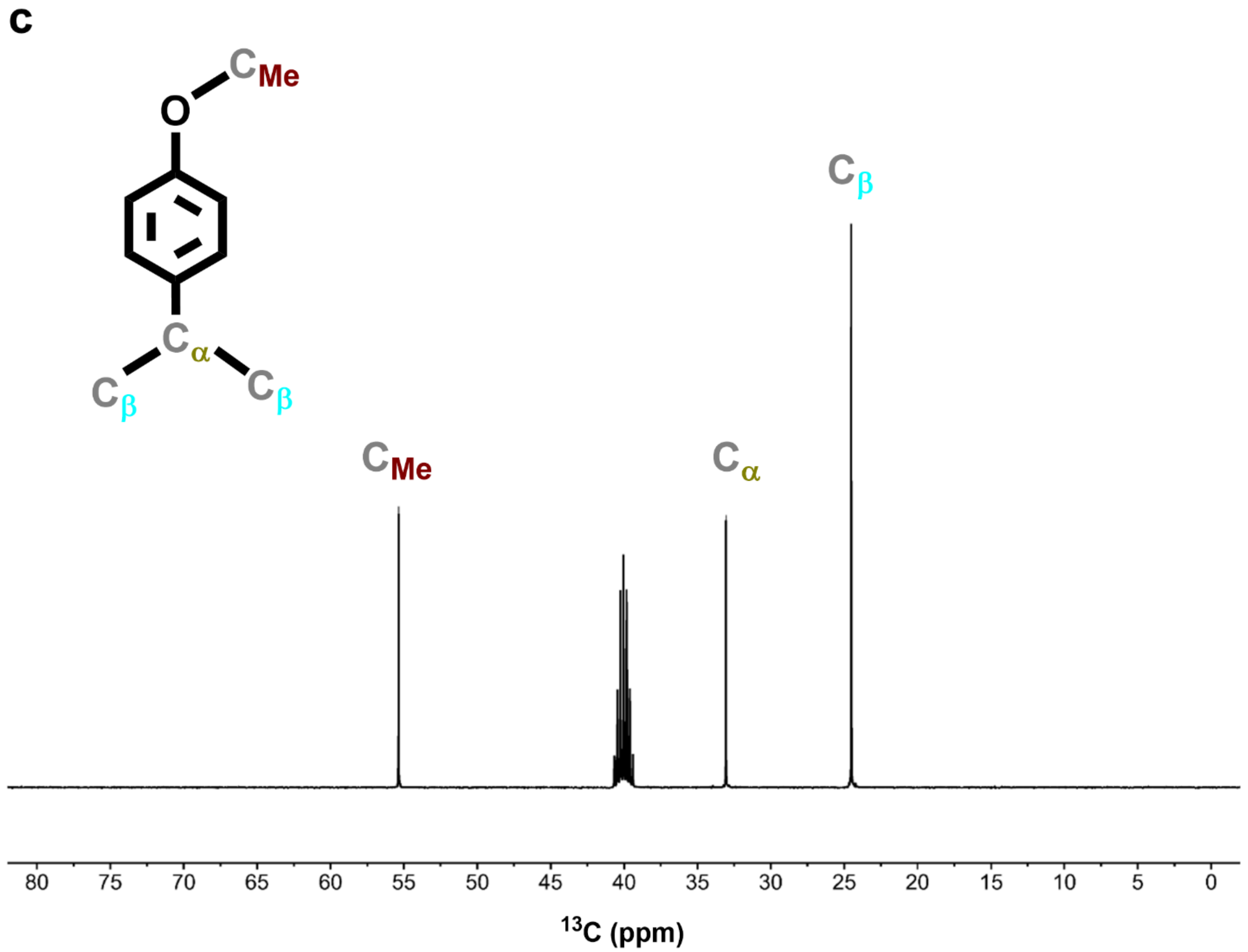
c
O
C_Me
C_α
C_β
C_β
C_Me
C_α
C_β
80 75 70 65 60 55 50 45 40 35 30 25 20 15 10 5 0
13C (ppm)

d

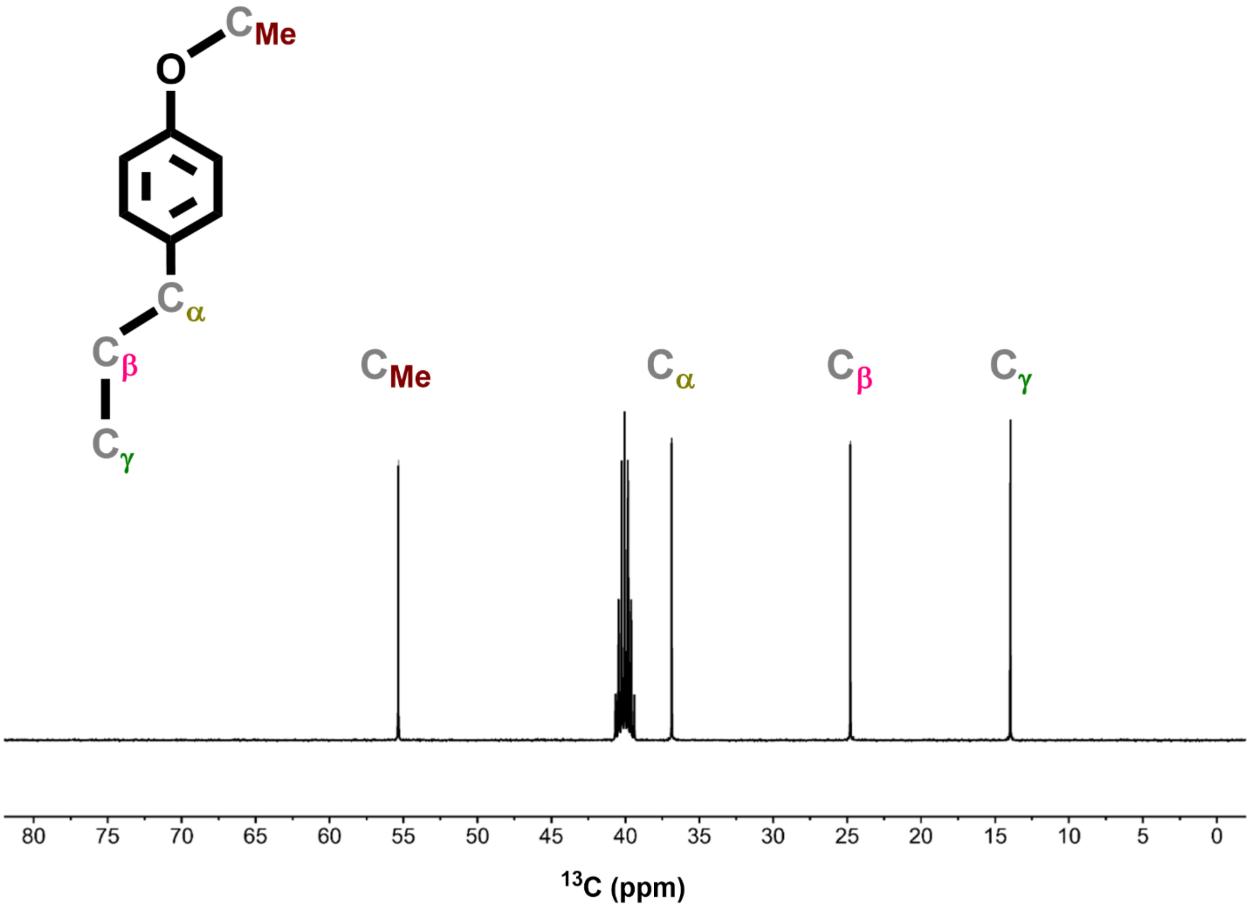

Supplementary Figure 7. ^{13}C NMR spectra of the model compounds in aliphatic region. a 1-(4-methoxyphenyl)-1-propanol (**1a**). **b** 2-(4-methoxyphenyl)propan-2-ol. **c** 2-(4'-methoxyphenyl)propane. **d** 1-methoxy-4-propylbenzene. C_α, C_β and C_γ represent the C atoms at the aliphatic α-C, β-C and γ-C positions, respectively.

a

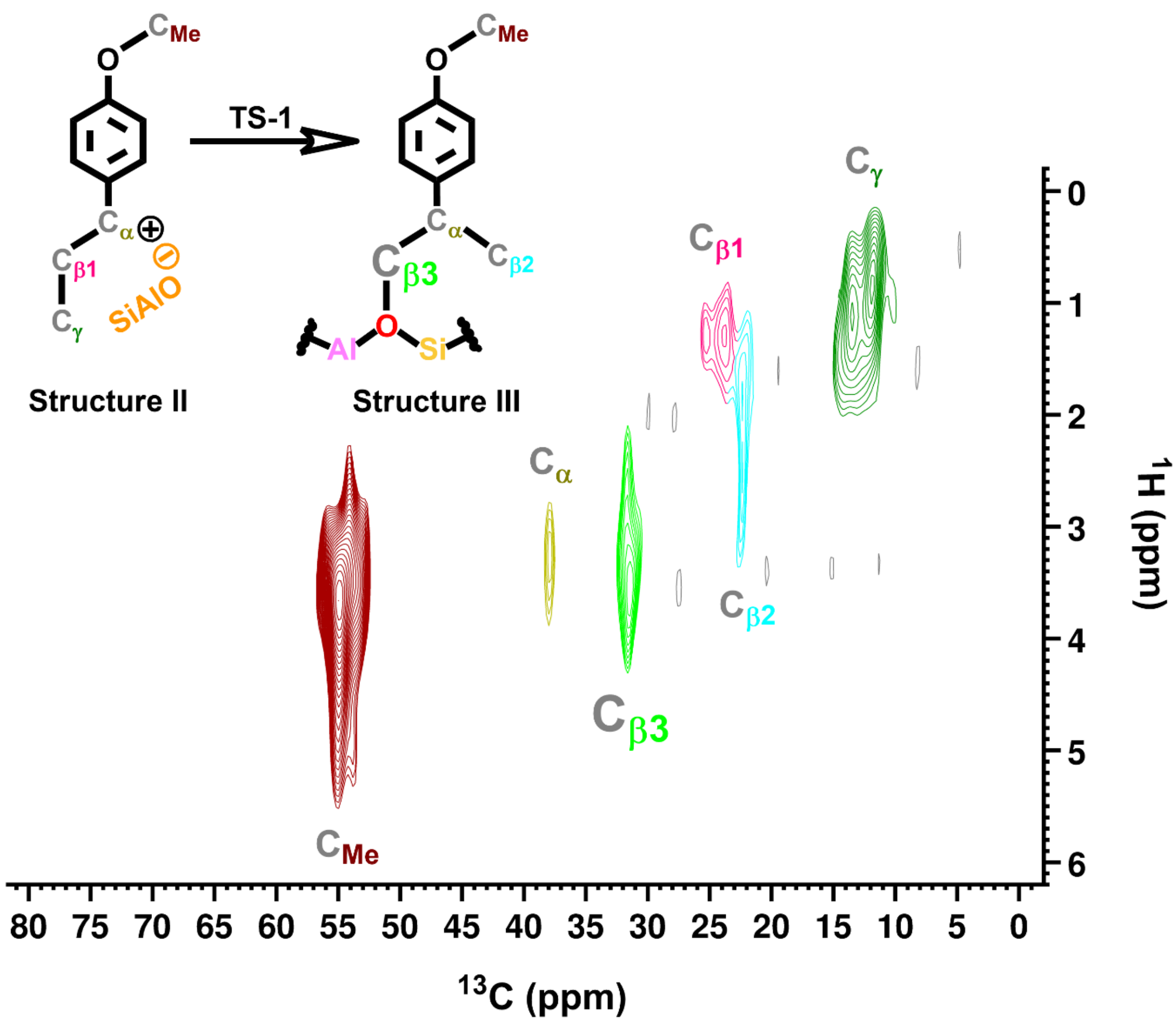

TS-1
Structure II
Structure III
SiAlO
Al
Si
80 75 70 65 60 55 50 45 40 35 30 25 20 15 10 5 0
13C (ppm)
1H (ppm)

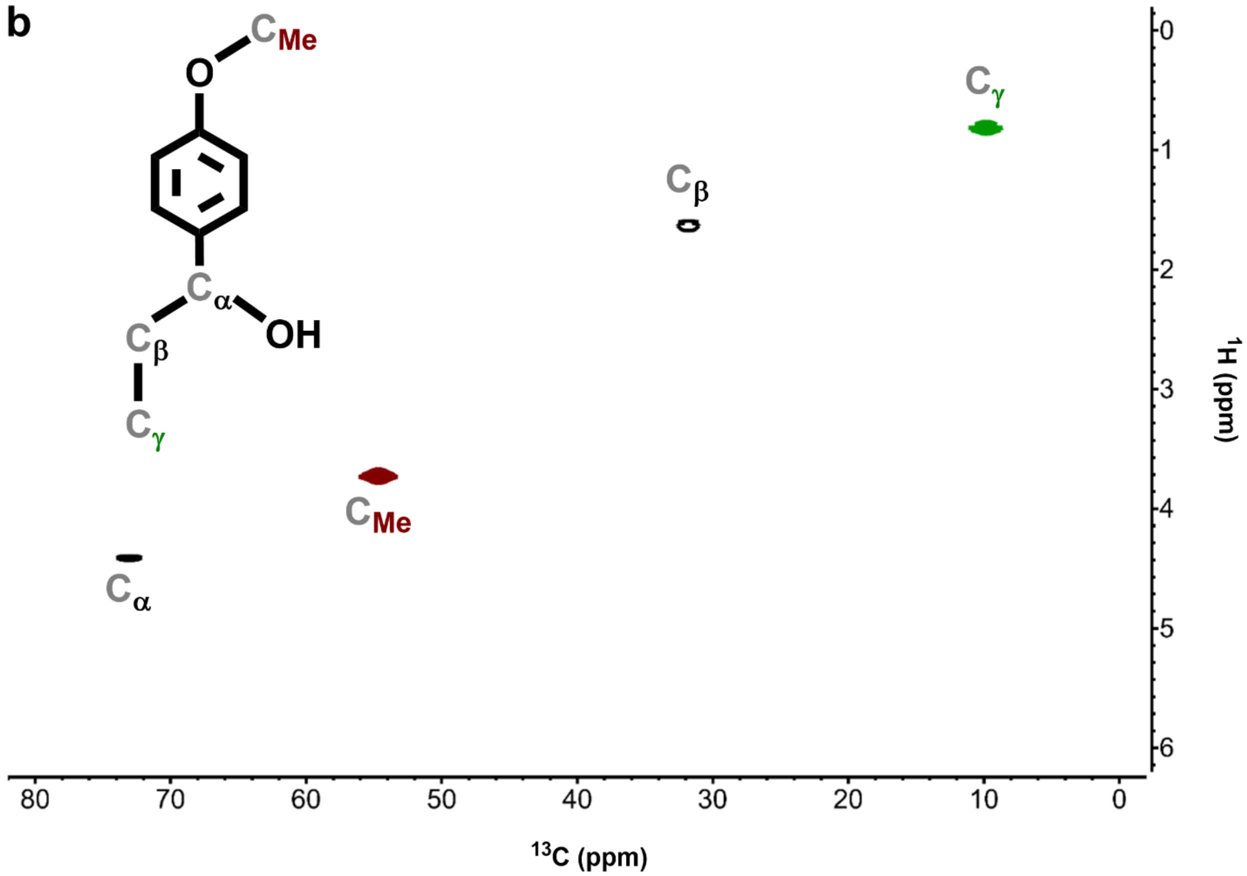

b
C
Me
O
OH
Cα
Cβ
Cγ
CMe
Cα
Cβ
Cγ
0
1
2
3
4
5
6
¹H (ppm)
80
70
60
50
40
30
20
10
0
¹³C (ppm)

c

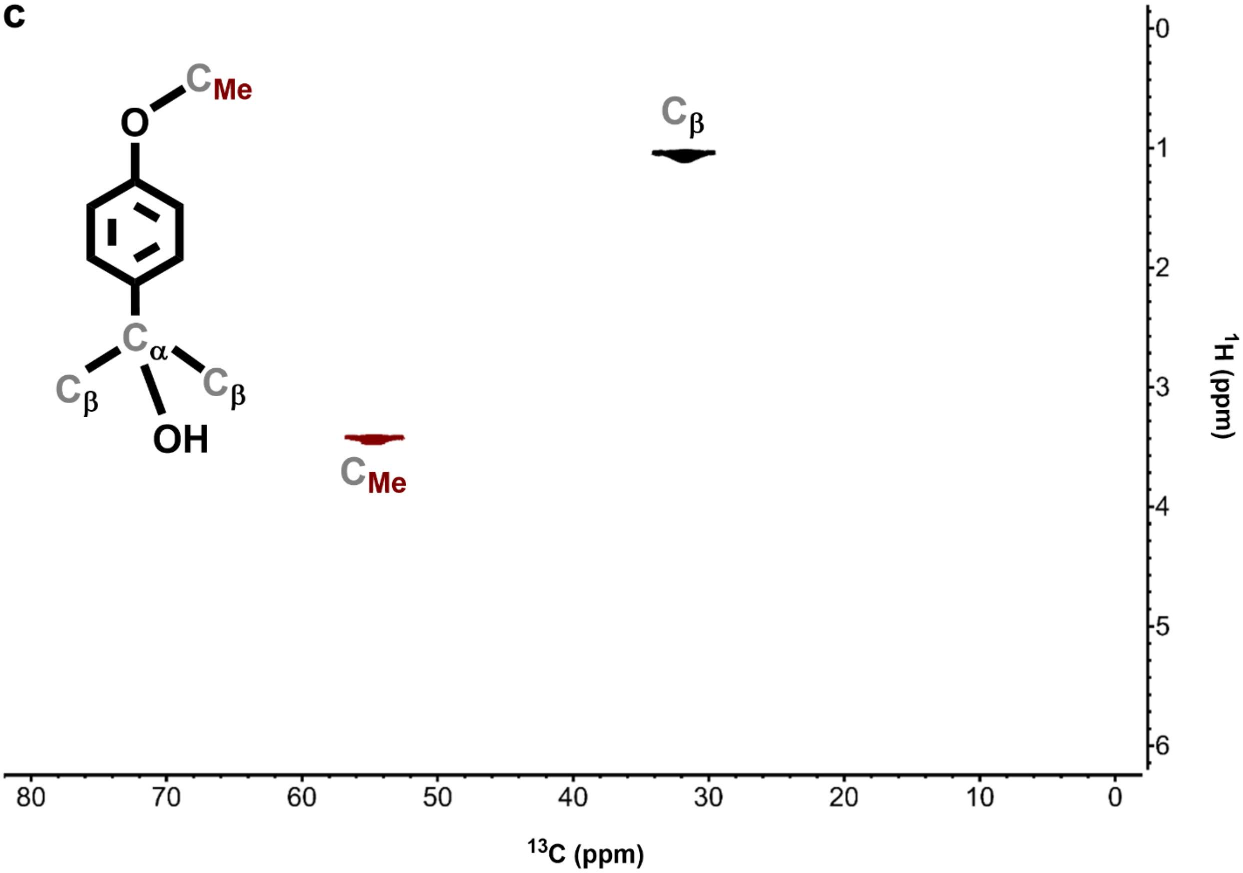

C_Me
O
C_α
C_β
C_β
OH
C_β
C_Me
0
1
2
3
4
5
6
¹H (ppm)
80
70
60
50
40
30
20
10
0
¹³C (ppm)

d

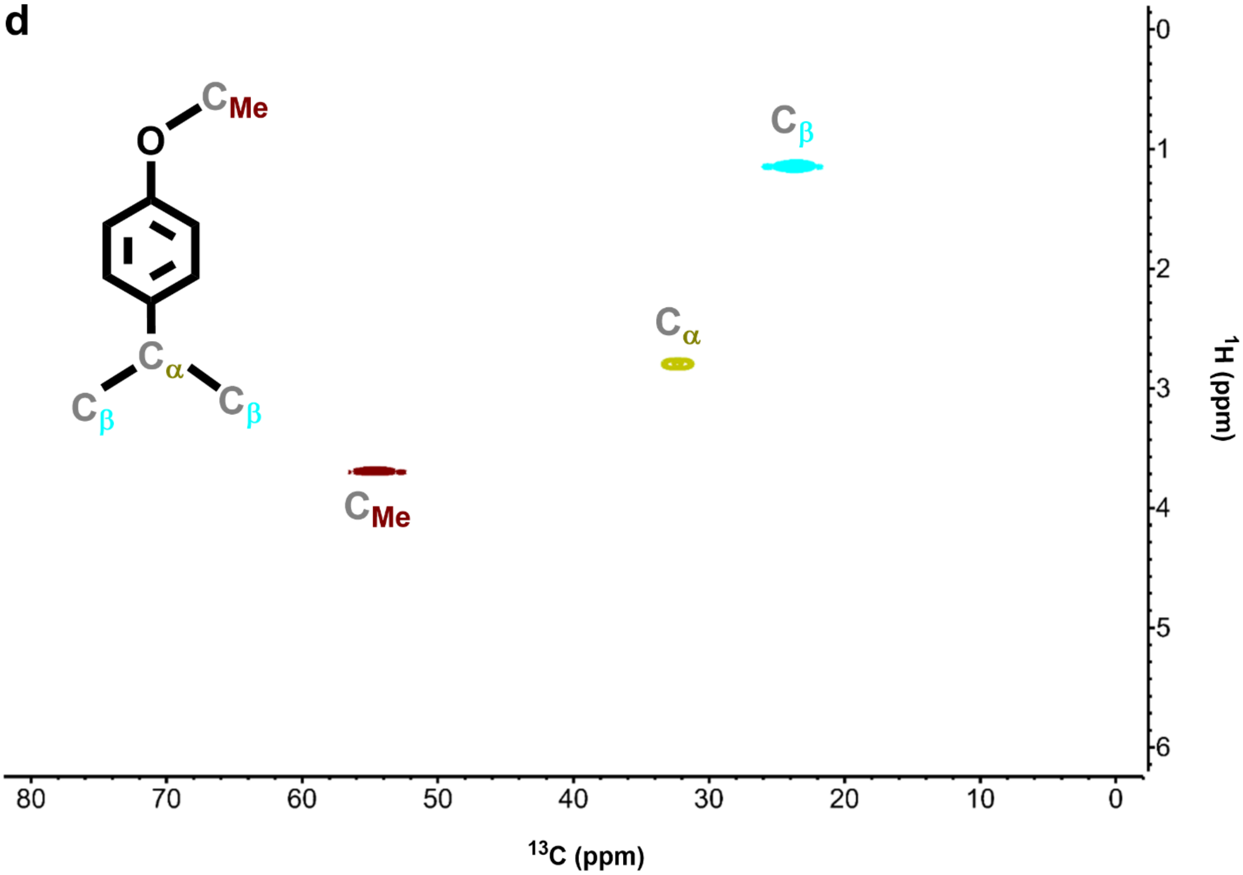

O
CMe
Cα
Cβ
Cβ
Cβ
Cα
CMe
0
1
2
3
4
5
6
1H (ppm)
80
70
60
50
40
30
20
10
0
13C (ppm)

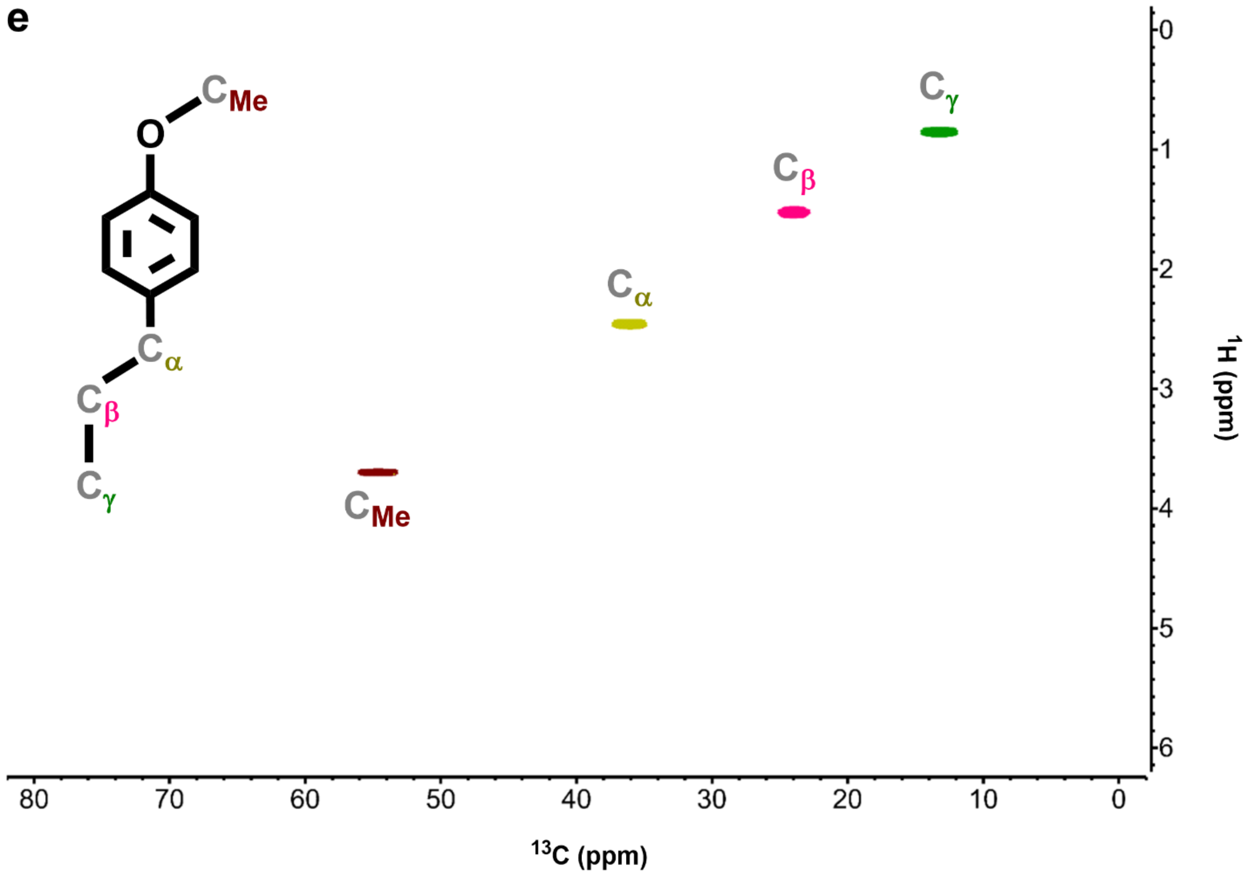

Supplementary Figure 8. 2D $^{13}C\{^{1}H\}$ HETCOR and HSQC NMR spectra. a Solid-state 2D $^{13}C\{^{1}H\}$ HETCOR NMR spectrum of the intermediates in the reaction of 1-(4-methoxyphenyl)-1-propanol (**1a**). **b** 2D HSQC NMR spectrum of **1a**. **c** 2D HSQC NMR spectrum of 2-(4-methoxyphenyl)propan-2-ol. **d** 2D HSQC NMR spectrum of 2-(4'-methoxyphenyl)propane. **e** 2D HSQC NMR spectrum of 1-methoxy-4-propylbenzene.

Reaction conditions: **1a** (1 mmol), RuW/HY_{30} (0.20 g, 2.5 wt% Ru, 14 wt% W), H_2O (5.0 ml), 180 ºC, 10 min, 0.1 MPa N_2, 800 rpm.

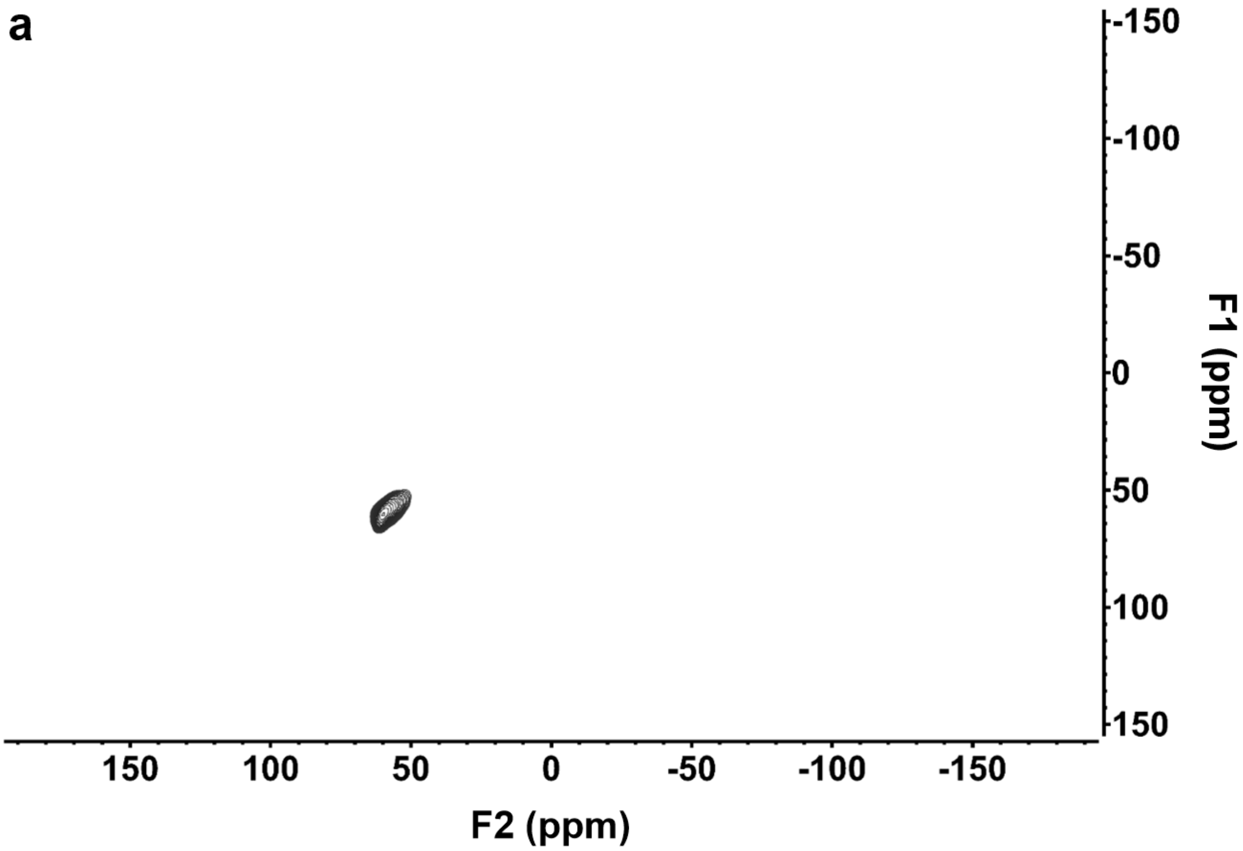
a
-150
-100
-50
0
50
100
150
F1 (ppm)
150
100
50
0
-50
-100
-150
F2 (ppm)

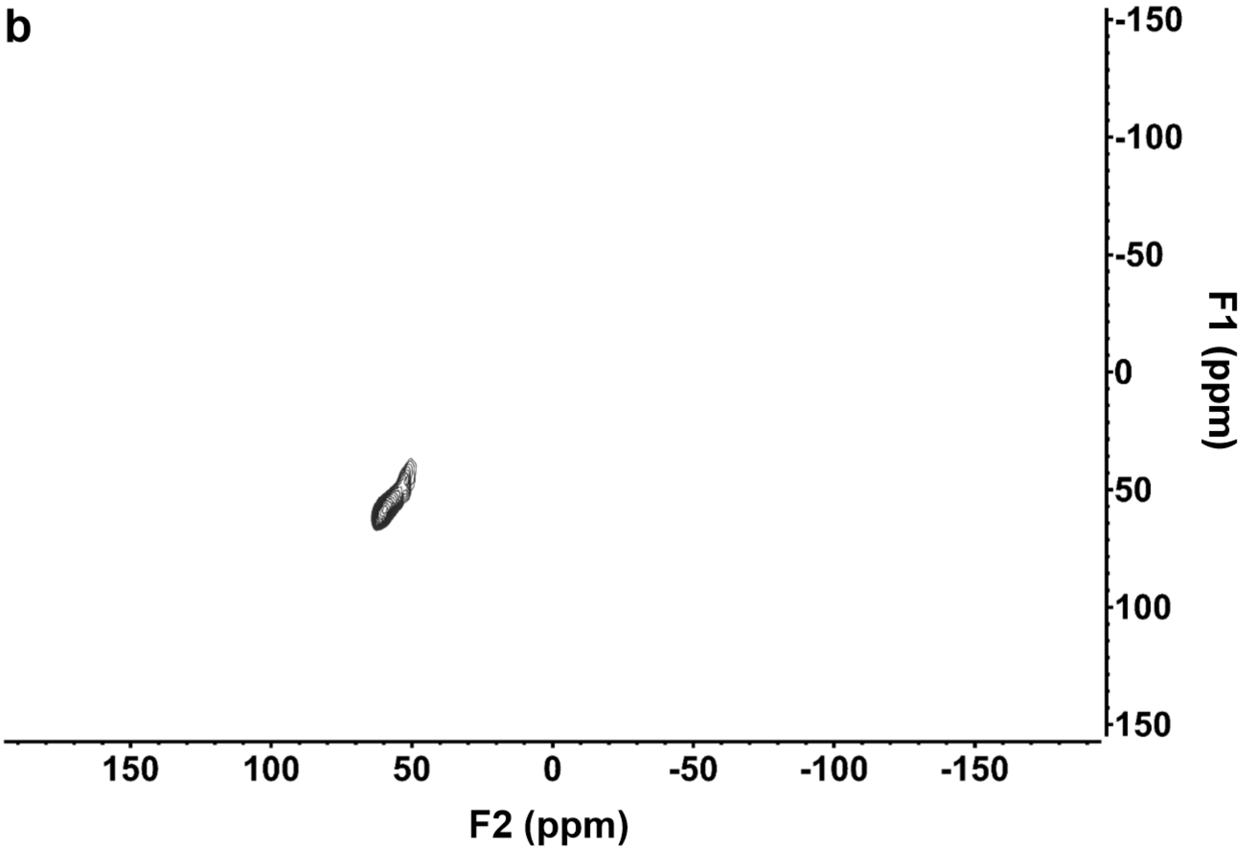

Supplementary Figure 9. ^{27}Al MQMAS NMR of the RuW/HY$_{30}$ catalyst. a ^{27}Al MQMAS analysis of the boiled RuW/HY$_{30}$ catalyst in the blank experiment, blue loops represent the regular framework Al(IV), black loops stand for the extra-framework Al(VI). **b** ^{27}Al MQMAS analysis of the RuW/HY$_{30}$ catalyst in reaction of **1a**, blue and green loops respectively represent the regular framework Al(IV) and the framework Al(IV) in the [Al-O(C$_{\beta3}$)-Si] center (Structure III, Fig. 3A), black loops represent the extra-framework Al(VI).

Reaction conditions: RuW/HY$_{30}$ (0.20 g, 2.5wt% Ru, 14wt% W), H_2O (5.0 ml), 180 ºC, 10 min, 0.1 MPa N_2, 800 rpm. **a** No reactant. **b 1a** (1 mmol).

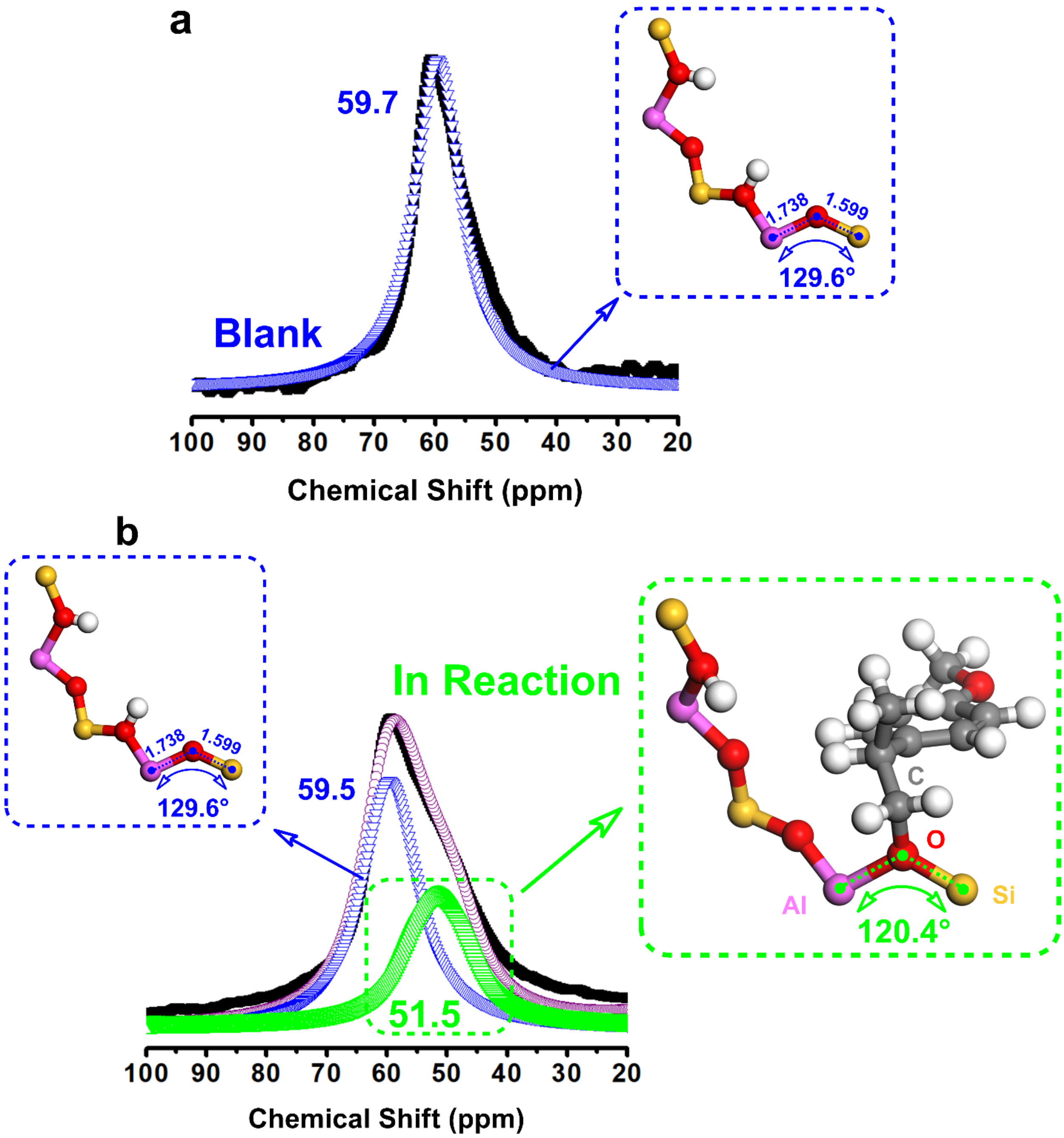

Supplementary Figure 10. ^{27}Al NMR of the RuW/HY$_{30}$ catalyst. a ^{27}Al NMR spectrum of the boiled RuW/HY$_{30}$ catalyst in the blank experiment, black solid square (■) represents the observed intensities, blue inverted triangle (▽) is the fitting curve of framework Al(IV). **b** ^{27}Al NMR spectrum of the RuW/HY$_{30}$ catalyst in reaction, black solid square (■) represents the observed intensities, purple circle marks (○) is the total fitting curve, blue inverted triangle (▽) and green regular triangle (△) are the fitting curves of the regular framework Al(IV) and the framework Al(IV) in the [Al-O($C_{\beta3}$)-Si] center (Structure III, Fig. 3), respectively.

Reaction conditions: RuW/HY$_{30}$ (0.20 g, 2.5 wt% Ru, 14 wt% W), H_2O (5.0 ml), 180 ºC, 10 min, 0.1 MPa N_2, 800 rpm. **a** No reactant. **b** **1a** (1 mmol).

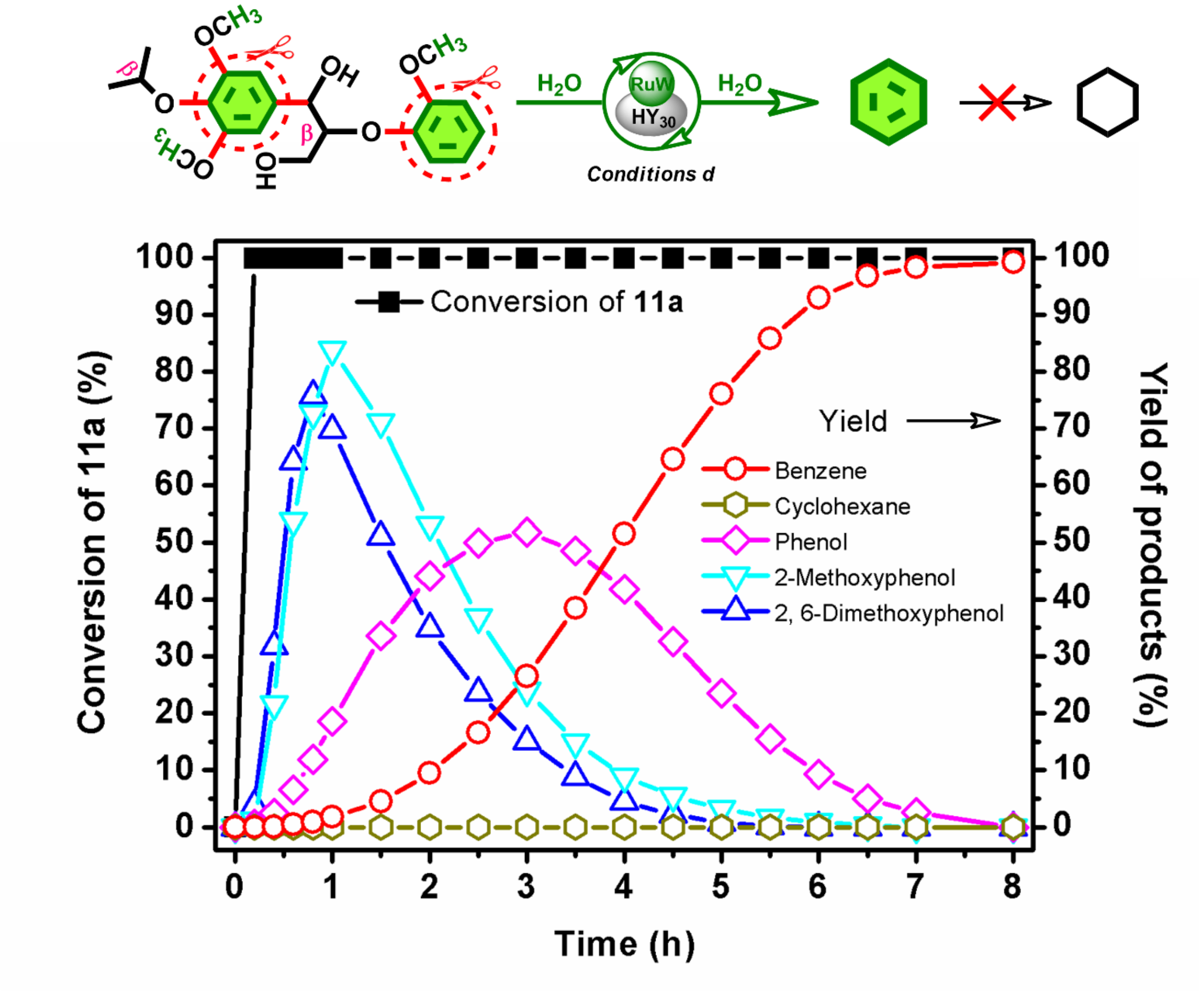

Supplementary Figure 11. Determination of the product distributions in the transformation of 1-(4-isopropoxy-3, 5-dimethoxyphenyl)-2-(2-methoxyphenoxy)propane-1, 3-diol (11a) over the RuW/HY_{30} catalyst.

Reaction conditions: **11a** (0.5 mmol), RuW/HY_{30} (0.35 g, 3.5 wt% Ru, 20 wt% W), H_2O (5.0 ml), 210 ºC, 0.1 MPa N_2, 800 rpm.

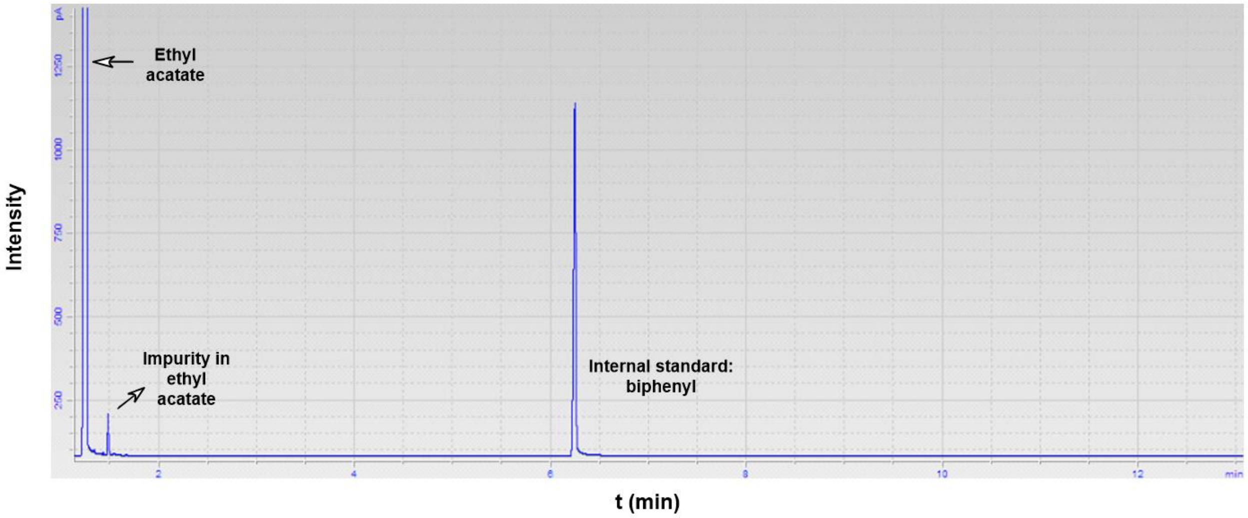

Supplementary Figure 12. GC trace of the blank experiment for the transformation of the pine lignin.

Reaction conditions: lignin (0.50 g), H_2O (6.5 mL), 240 ºC, 0.1 MPa N_2, 12 h, 800 rpm.

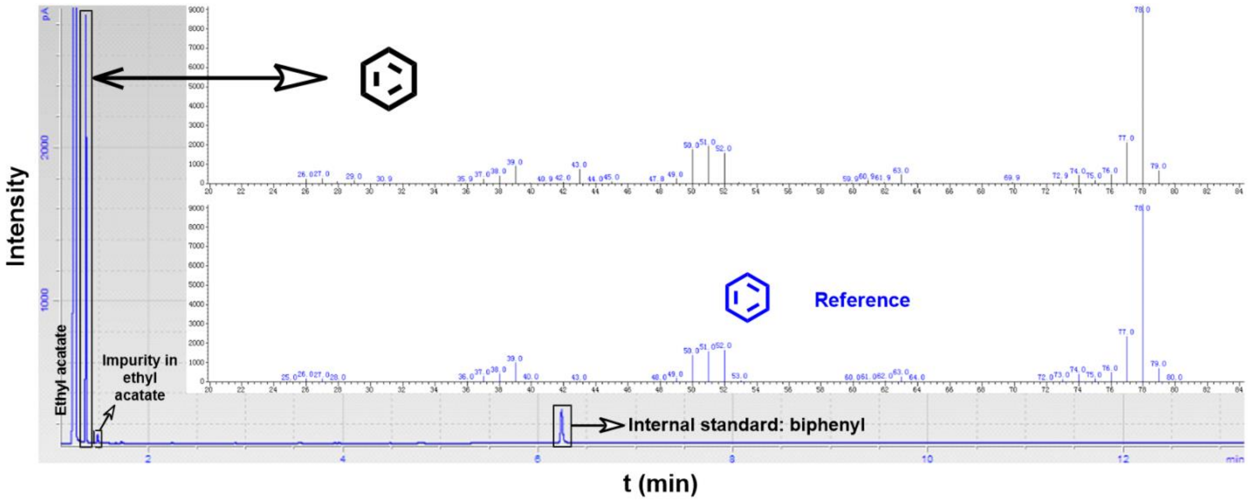

Supplementary Figure 13. GC trace of the in situ refining experiment for the transformation of the pine lignin.

Reaction conditions: lignin (0.50 g), RuW/HY_{30} (0.50 g, 3.5 wt% Ru, 20 wt% W), H_2O (6.5 mL), 240 ºC, 0.1 MPa N_2, 12 h, 800 rpm.

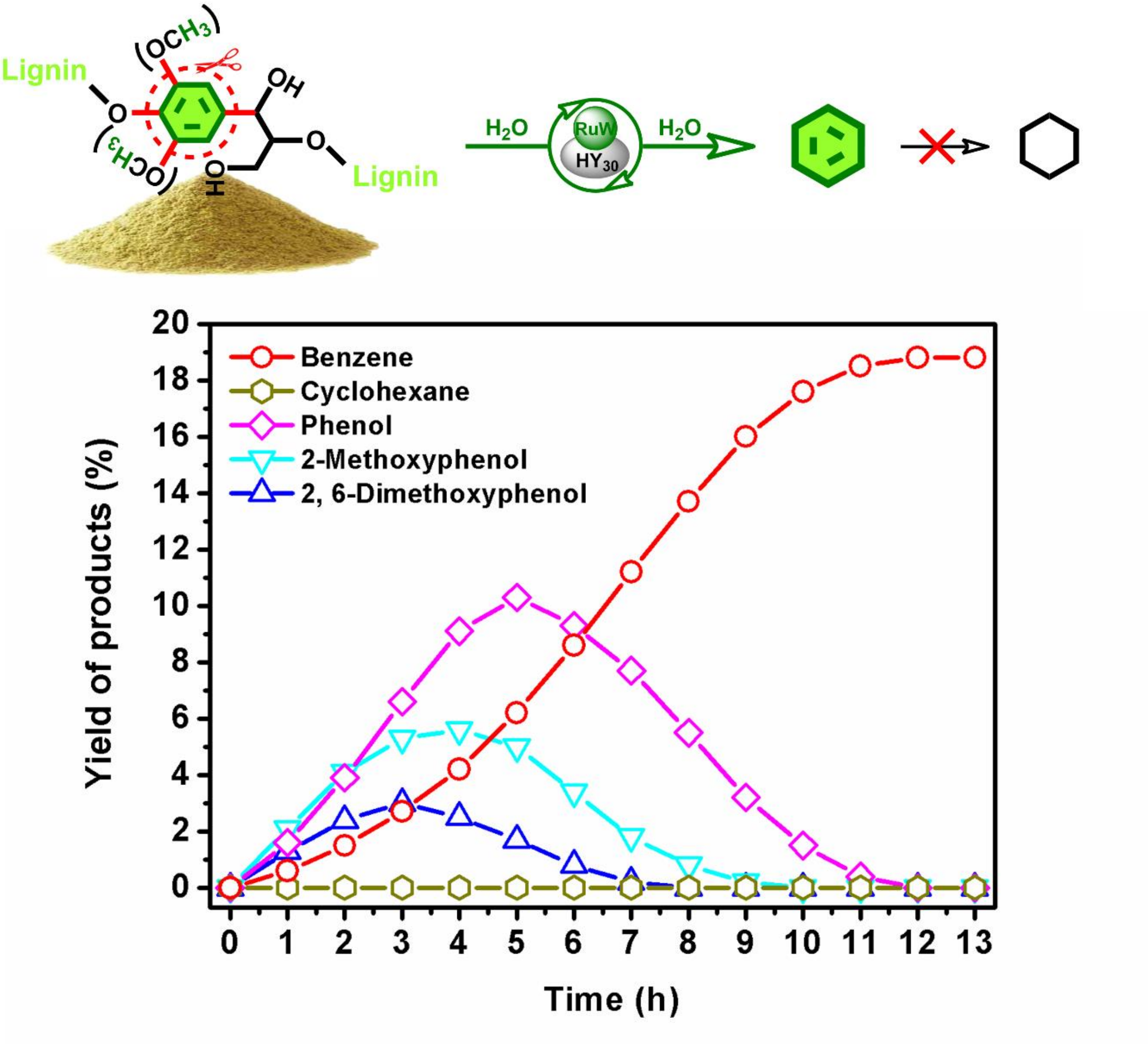

Supplementary Figure 14. Determination of the product distributions during the transformation of the pine lignin.

Reaction conditions: lignin (0.50 g), RuW/HY30 (0.50 g, 3.5 wt% Ru, 20 wt% W), H_2O (6.5 ml), 240 ºC, 0.1 MPa N_2, 800 rpm.

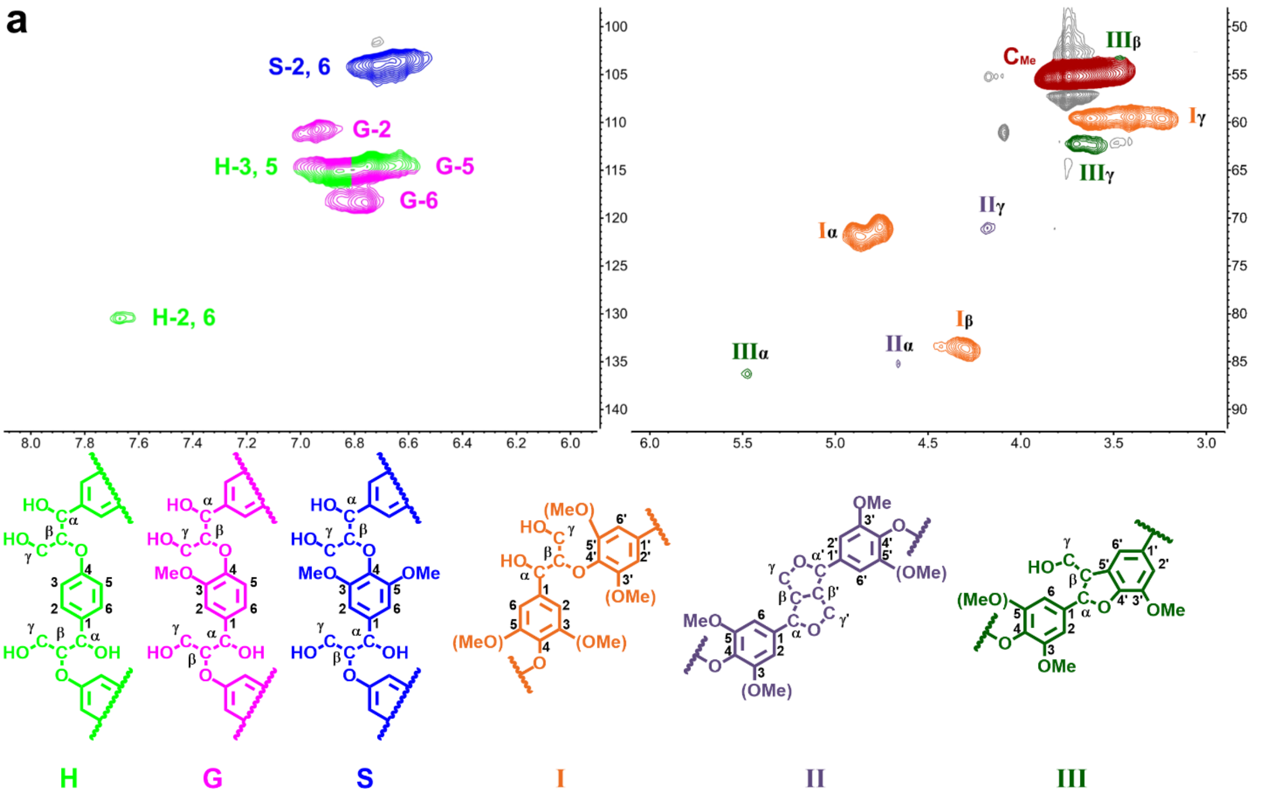
a
S-2, 6
G-2
H-3, 5
G-5
G-6
H-2, 6
CMe
IIIβ
Iγ
IIIγ
IIγ
Iα
Iβ
IIα
IIIα
H
G
S
I
II
III

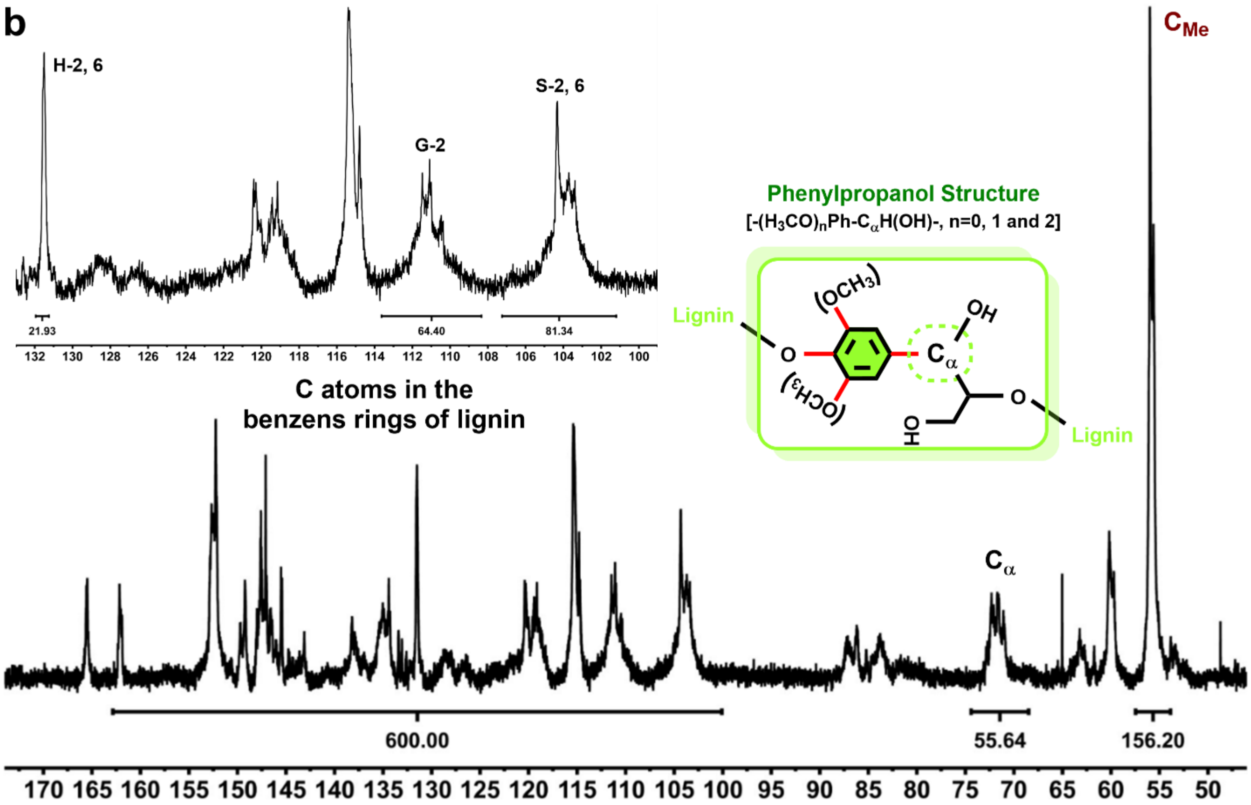

Supplementary Figure 15. $^{1}H/^{13}C$ 2D-HSQC and quantitative ^{13}C NMR spectra of the pine lignin. a $^{1}H/^{13}C$ 2D-HSQC NMR spectra. **b** Quantitative ^{13}C NMR spectra (inset: the content of H (hydroxyphenyl), G (guaiacyl) and S (syringyl)-derived building blocks in lignin).

(H) p-Hydroxyphenyl unit; (G) guaiacyl unit; (S) syringyl unit; (I) β-aryl ether (β-O-4); (II) resinol (β-β); (III) phenylcoumaran (β-5); C_α, the C atom in the phenylpropanol structure; C_{Me}, the C atom in the methoxy group.

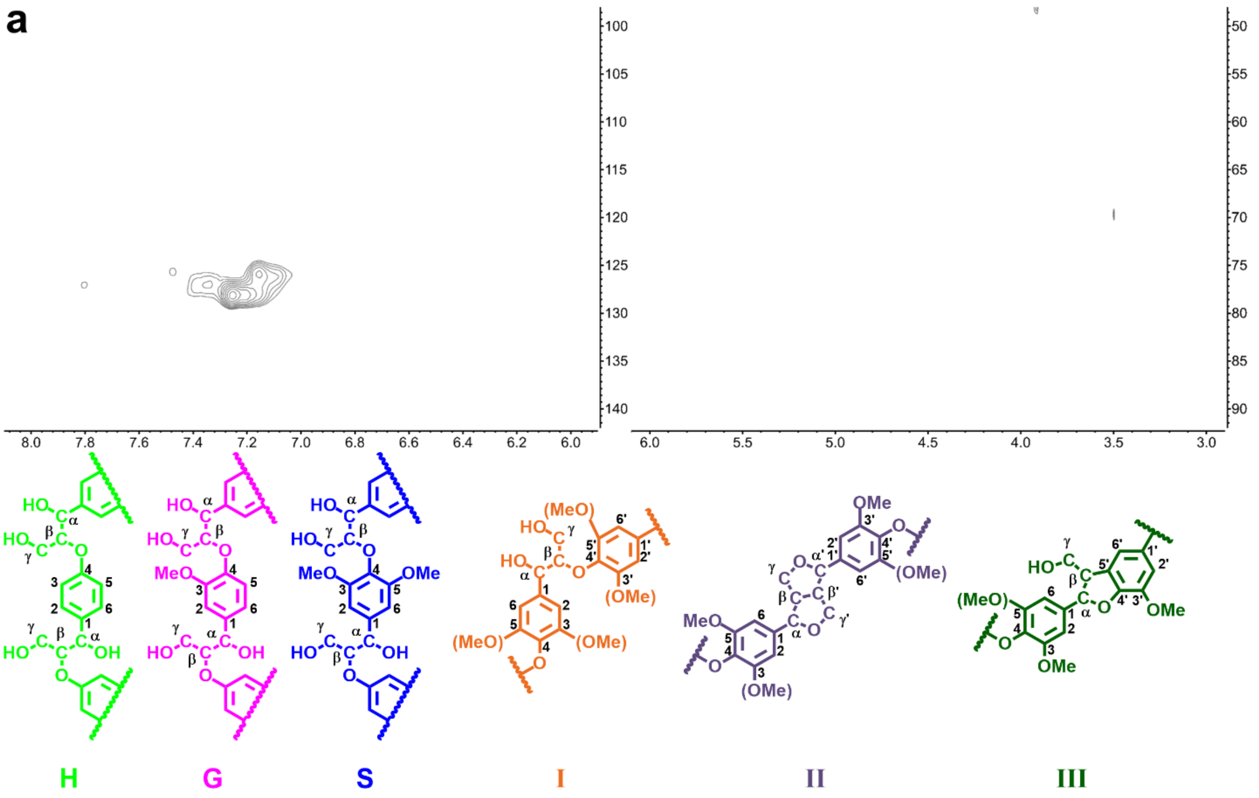
a
H
G
S
I
II
III

b

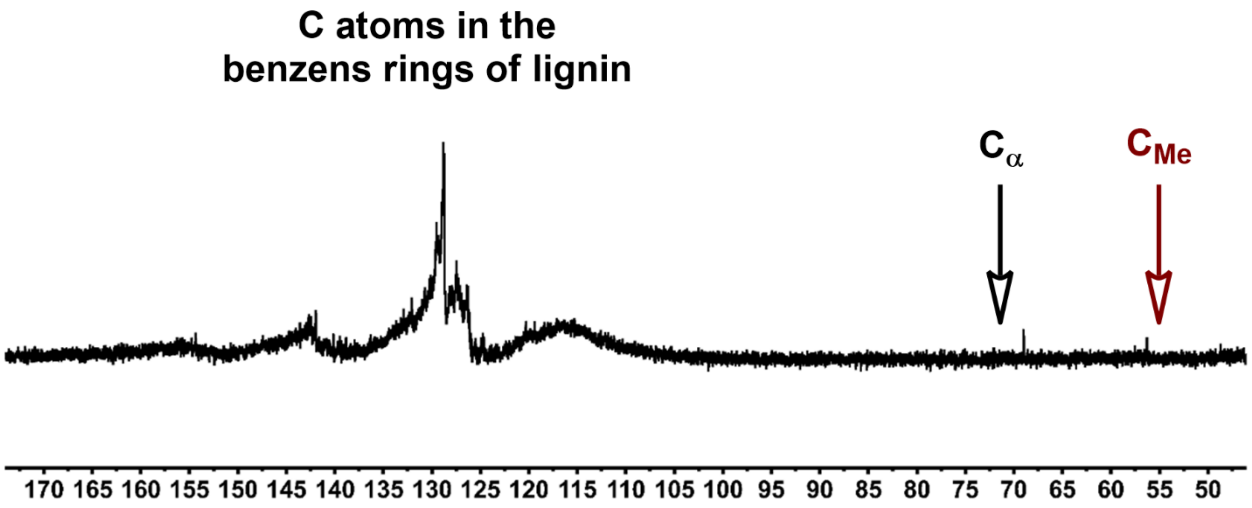

Supplementary Figure 16. $^{1}H/^{13}C$ 2D-HSQC and quantitative ^{13}C NMR spectra of the lignin residue obtained in the transformation of the pine lignin. a $^{1}H/^{13}C$ 2D-HSQC NMR spectra. b Quantitative ^{13}C NMR spectra.

(H) p-Hydroxyphenyl unit; (G) guaiacyl unit; (S) syringyl unit; (I) β-aryl ether (β-O-4); (II) resinol (β-β); (III) phenylcoumaran (β-5); C_{α}, the C atom in the phenylpropanol structure; C_{Me}, the C atom in the methoxy group.

Reaction conditions: lignin (0.50 g), RuW/HY_{30} (0.50 g, 3.5 wt% Ru, 20 wt% W), H_2O (6.5 ml), 240 ºC, 12h, 0.1 MPa N_2, 800 rpm.

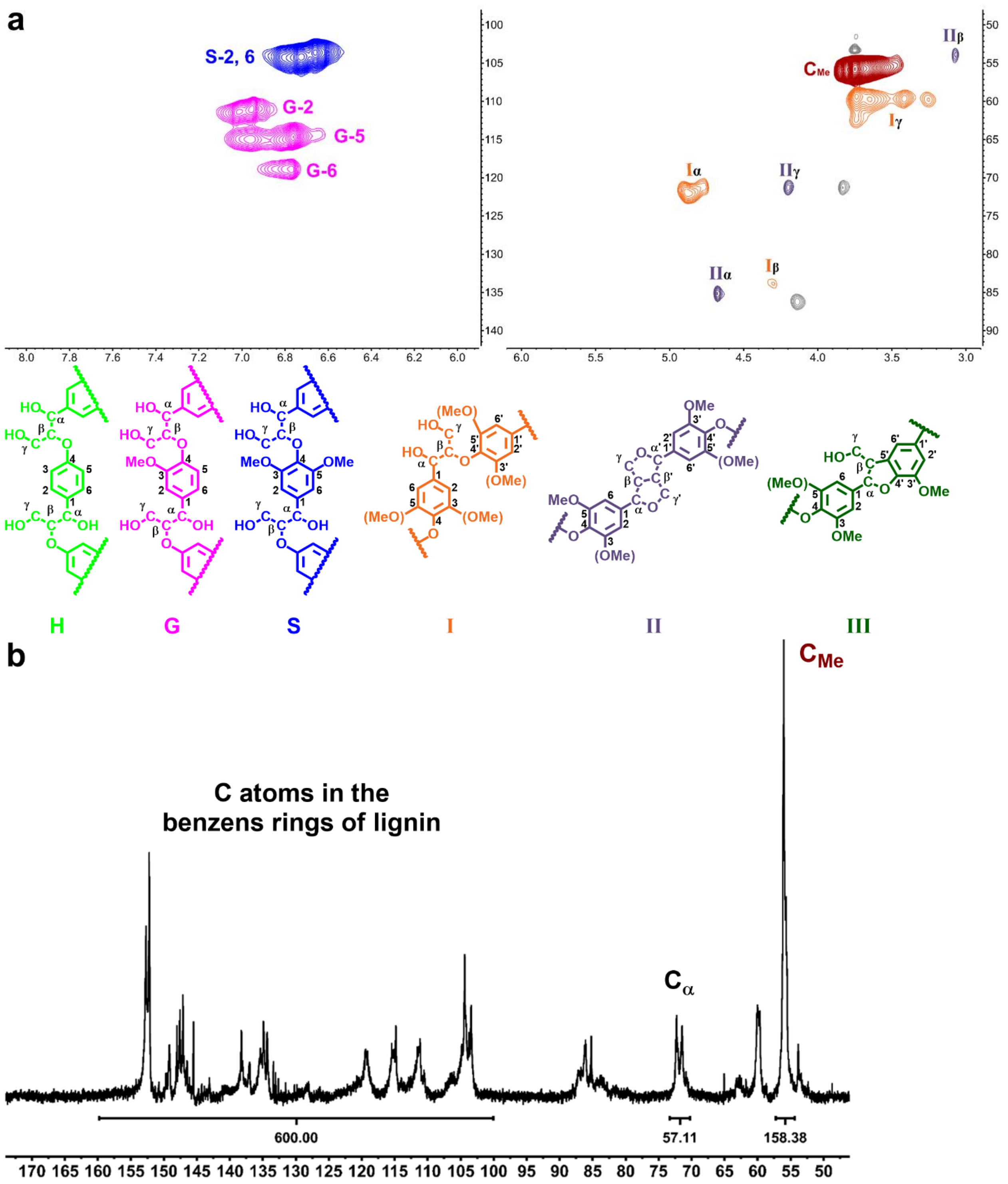

Supplementary Figure 17. $^{1}H/^{13}C$ 2D-HSQC and quantitative ^{13}C NMR spectra of the cedrela lignin. a $^{1}H/^{13}C$ 2D-HSQC NMR spectra. **b** Quantitative ^{13}C NMR spectra.

(H) p-Hydroxyphenyl unit; (G) guaiacyl unit; (S) syringyl unit; (I) β-aryl ether (β-O-4); (II) resinol (β-β); (III) phenylcoumaran (β-5); C_{α}, the C atom in the phenylpropanol structure; C_{Me}, the C atom in the methoxy group.

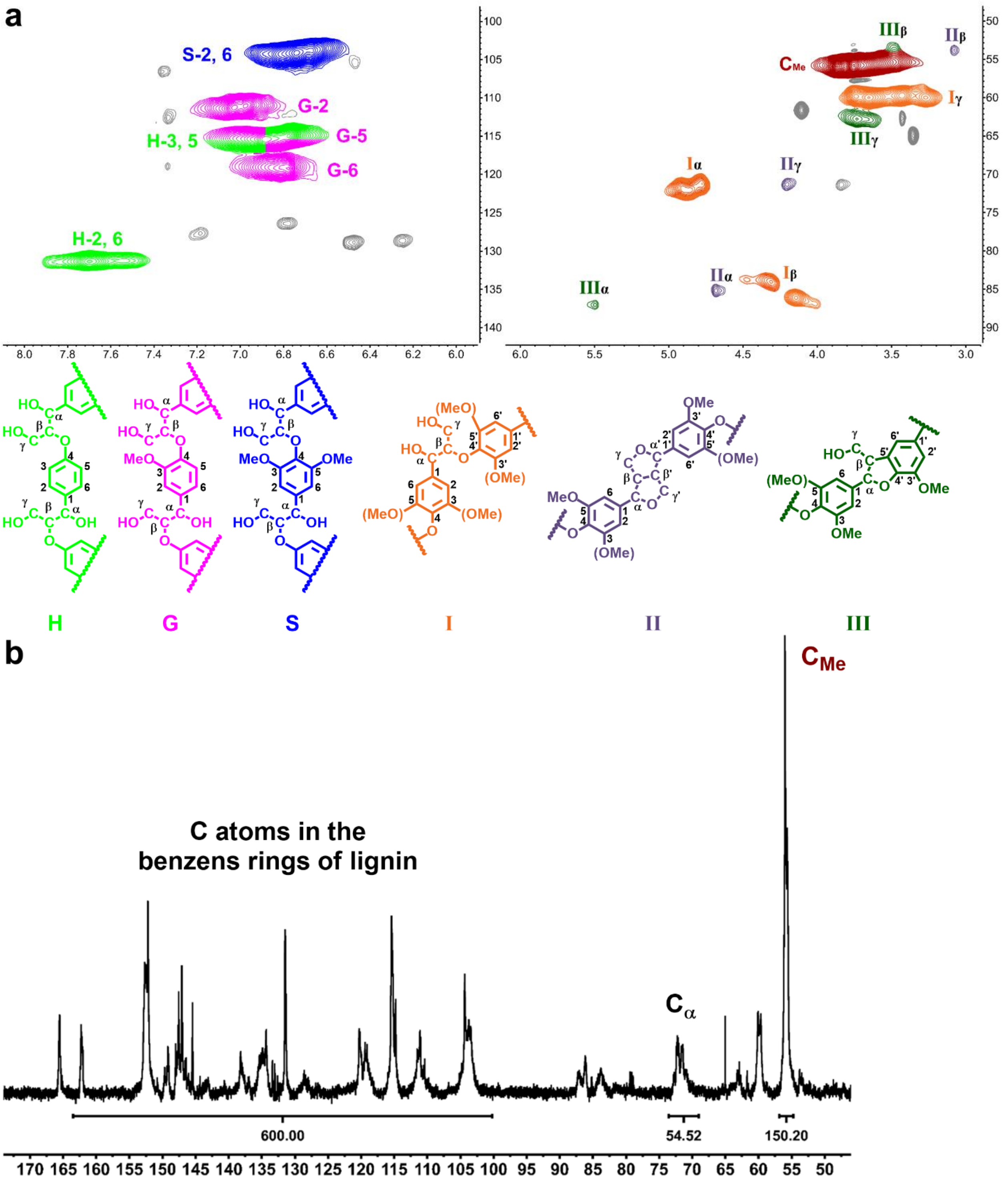

Supplementary Figure 18. $^{1}H/^{13}C$ 2D-HSQC and quantitative ^{13}C NMR spectra of the poplar lignin. a $^{1}H/^{13}C$ 2D-HSQC NMR spectra. **b** Quantitative ^{13}C NMR spectra.

(H) p-Hydroxyphenyl unit; (G) guaiacyl unit; (S) syringyl unit; (I) β-aryl ether (β-O-4); (II) resinol (β-β); (III) phenylcoumaran (β-5); C_{α}, the C atom in the phenylpropanol structure; C_{Me}, the C atom in the methoxy group.

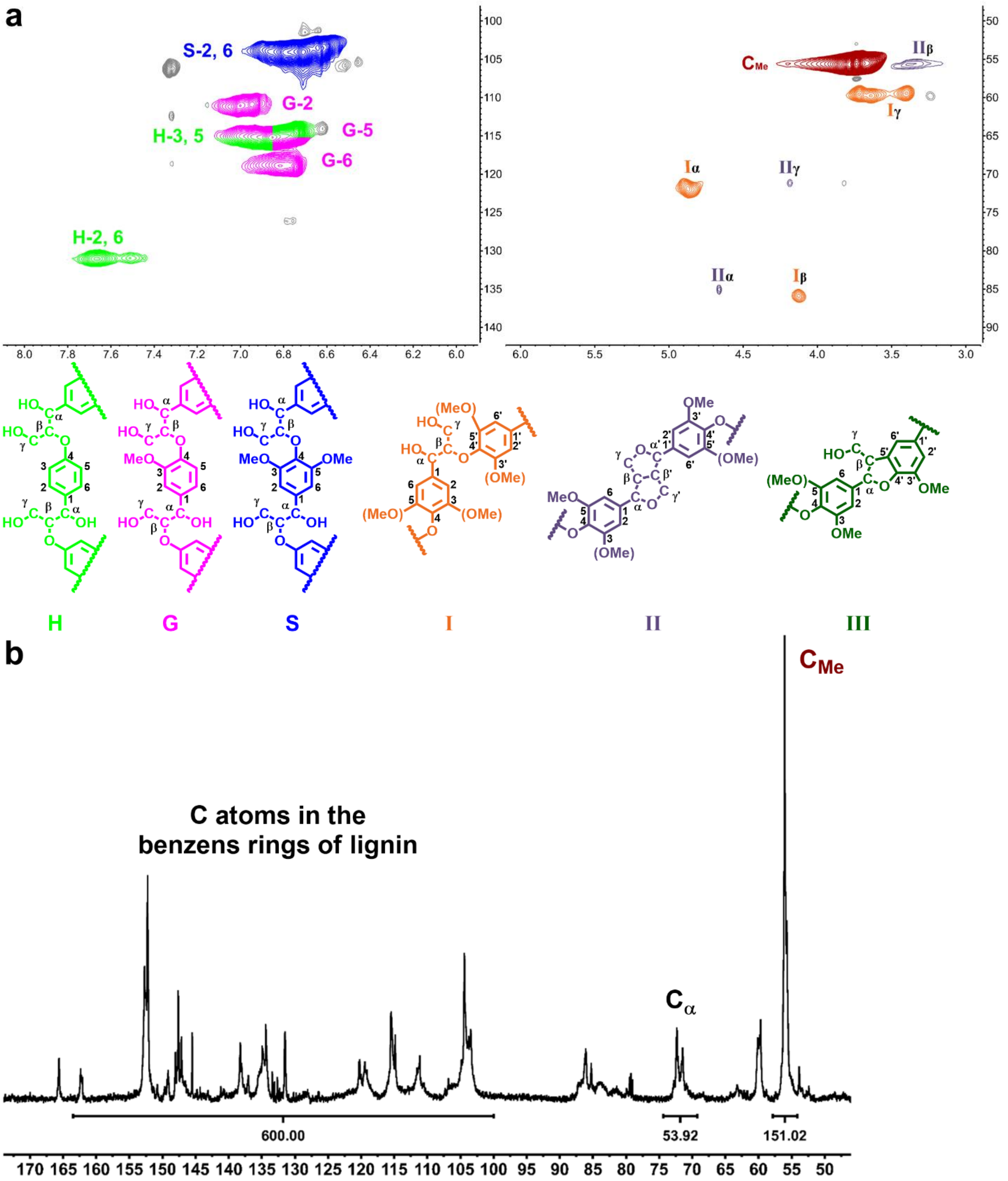

Supplementary Figure 19. $^1H/^{13}C$ 2D-HSQC and quantitative ^{13}C NMR spectra of the willow lignin. a $^1H/^{13}C$ 2D-HSQC NMR spectra. **b** Quantitative ^{13}C NMR spectra.

(H) p-Hydroxyphenyl unit; (G) guaiacyl unit; (S) syringyl unit; (I) β-aryl ether (β-O-4); (II) resinol (β-β); (III) phenylcoumaran (β-5); C_{α}, the C atom in the phenylpropanol structure; C_{Me}, the C atom in the methoxy group.

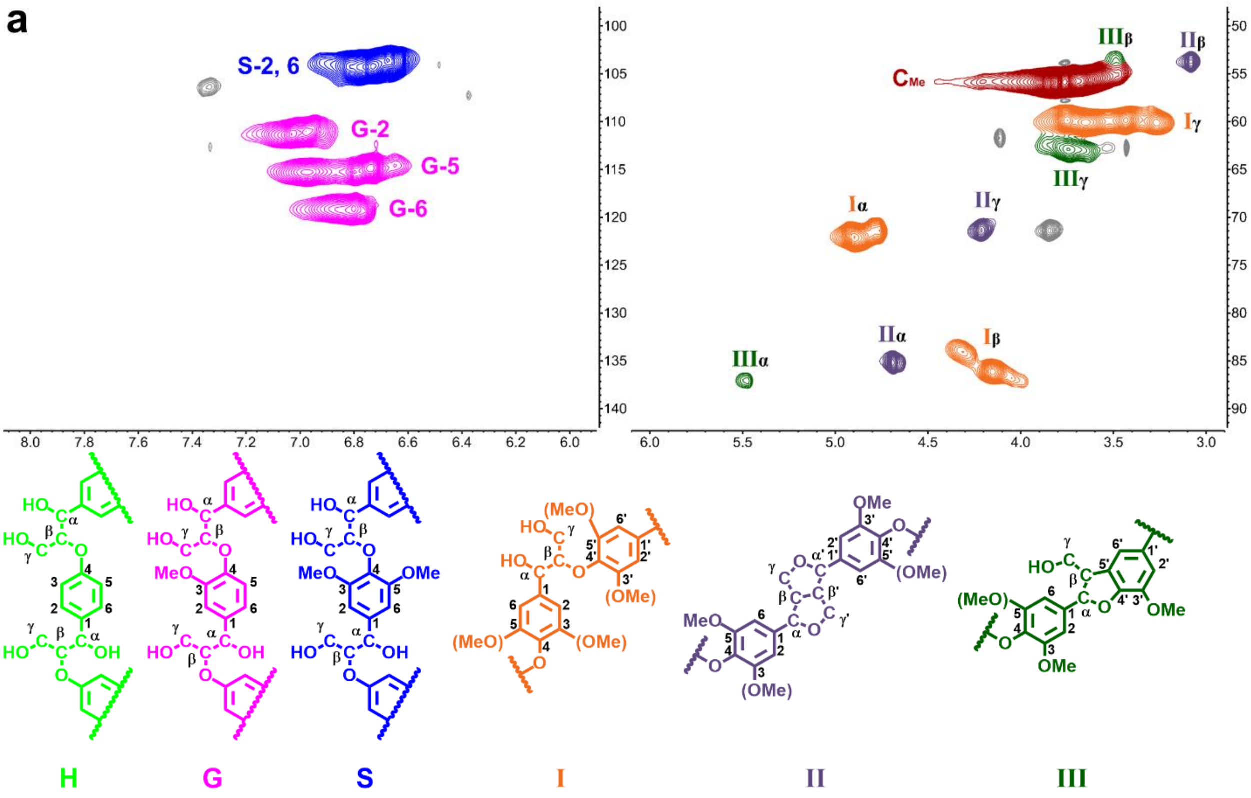
a
S-2, 6
G-2
G-5
G-6
CMe
IIIβ
IIβ
Iγ
IIIγ
Iα
IIγ
IIα
Iβ
IIIα
H
G
S
I
II
III

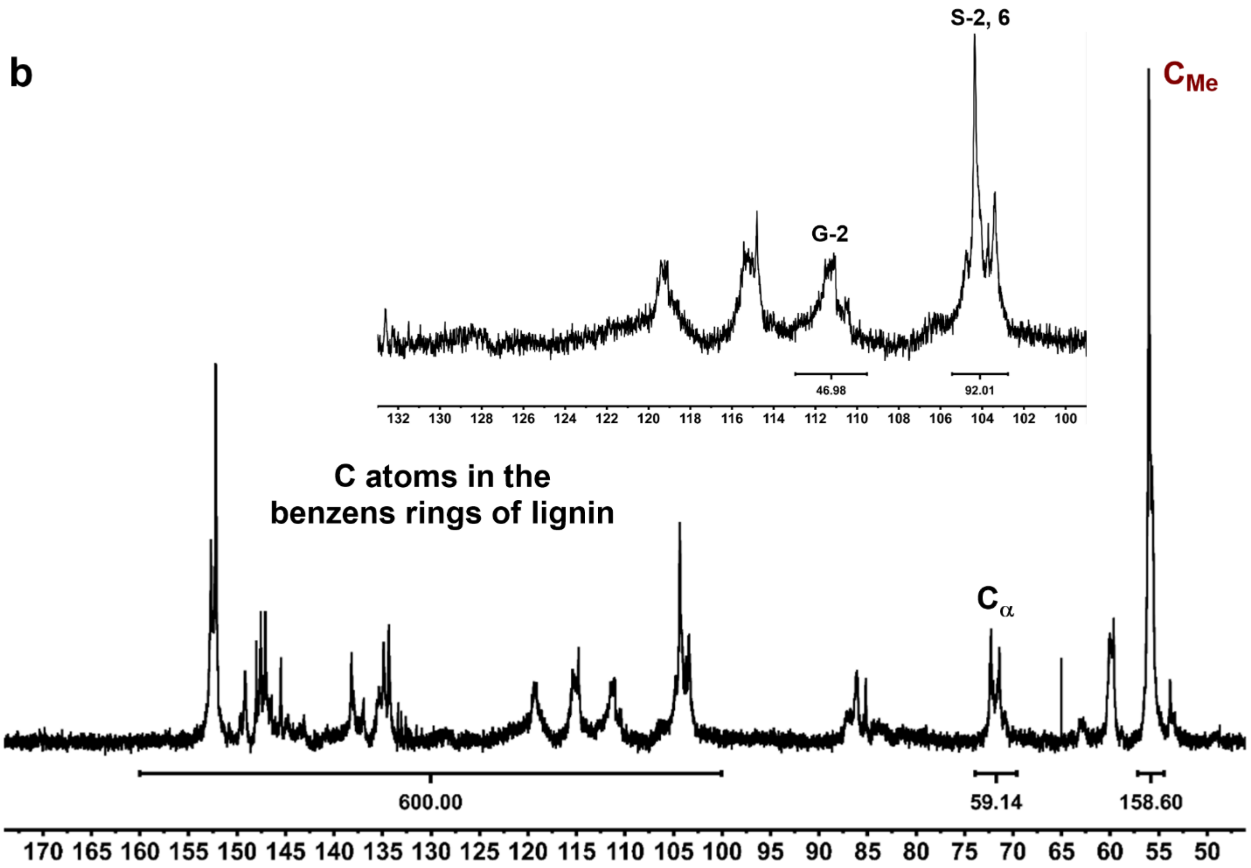

Supplementary Figure 20. $^{1}H/^{13}C$ 2D-HSQC and quantitative ^{13}C NMR spectra of the eucalyptus lignin. a $^{1}H/^{13}C$ 2D-HSQC NMR spectra. **b** Quantitative ^{13}C NMR spectra (inset: the content of G (guaiacyl) and S (syringyl)-derived building blocks in lignin).

(H) p-Hydroxyphenyl unit; (G) guaiacyl unit; (S) syringyl unit; (I) β-aryl ether (β-O-4); (II) resinol (β-β); (III) phenylcoumaran (β-5); C_α, the C atom in the phenylpropanol structure; C_{Me}, the C atom in the methoxy group.

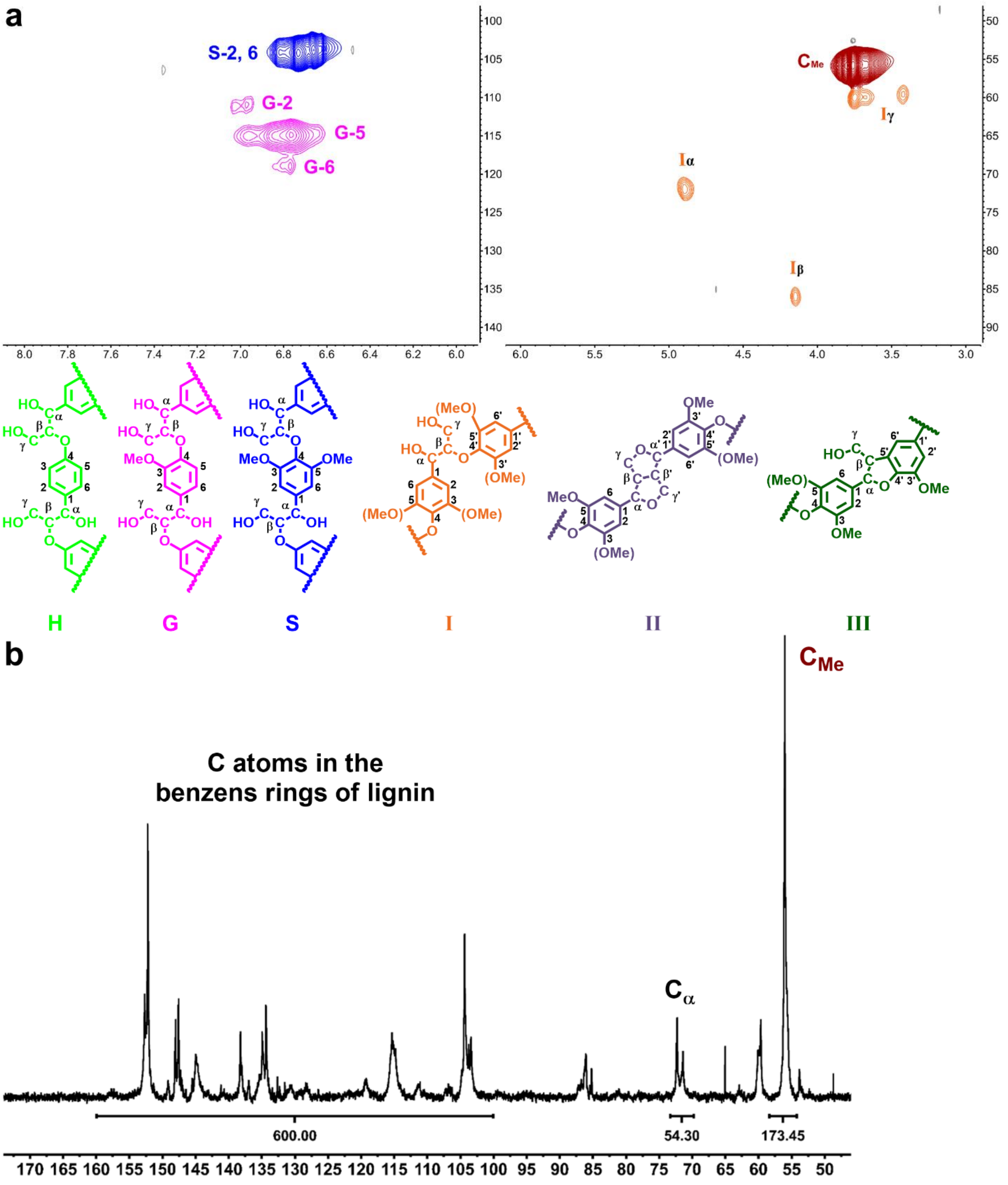

Supplementary Figure 21. $^{1}H/^{13}C$ 2D-HSQC and quantitative ^{13}C NMR spectra of the peach lignin. a $^{1}H/^{13}C$ 2D-HSQC NMR spectra. **b** Quantitative ^{13}C NMR spectra.

(H) p-Hydroxyphenyl unit; (G) guaiacyl unit; (S) syringyl unit; (I) β-aryl ether (β-O-4); (II) resinol (β-β); (III) phenylcoumaran (β-5); C_{α}, the C atom in the phenylpropanol structure; C_{Me}, the C atom in the methoxy group.

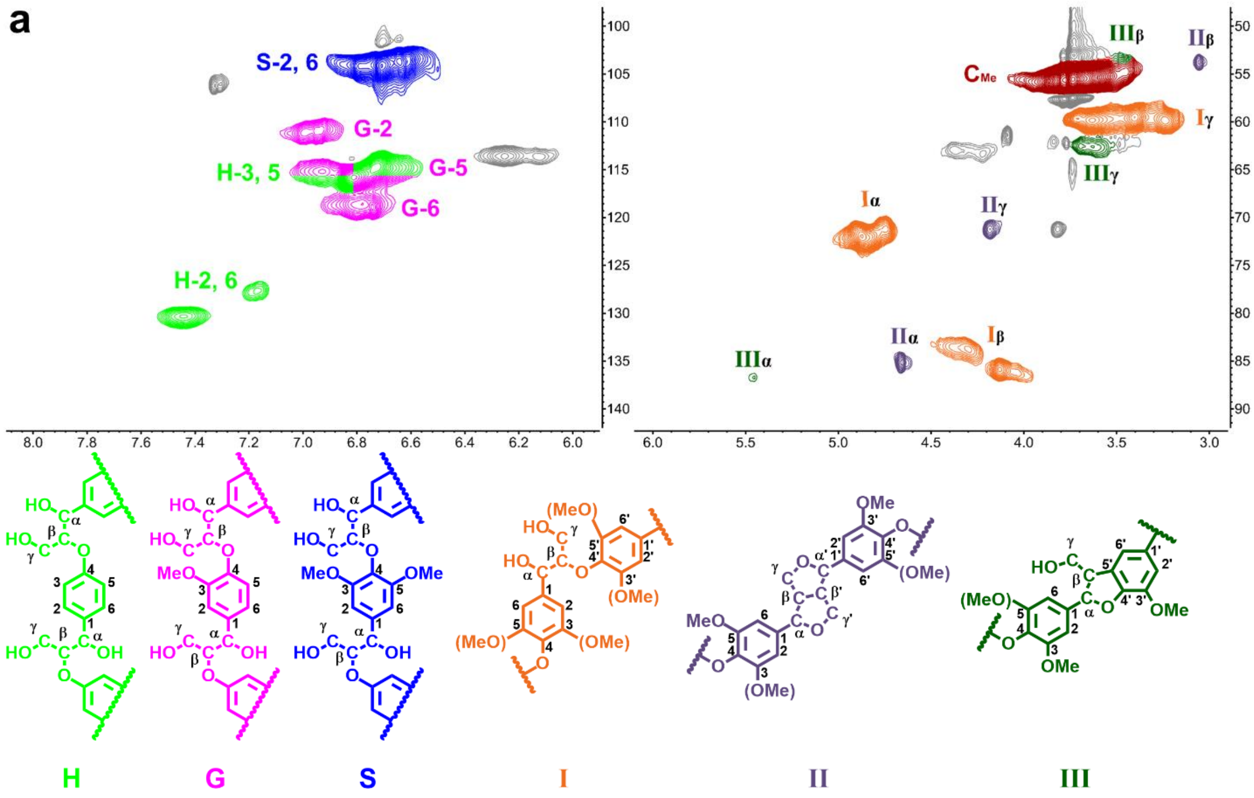
a
S-2, 6
G-2
H-3, 5
G-5
G-6
H-2, 6
C_{Me}
IIIβ
IIβ
Iγ
IIIγ
Iα
IIγ
IIα
Iβ
IIIα
H
G
S
I
II
III

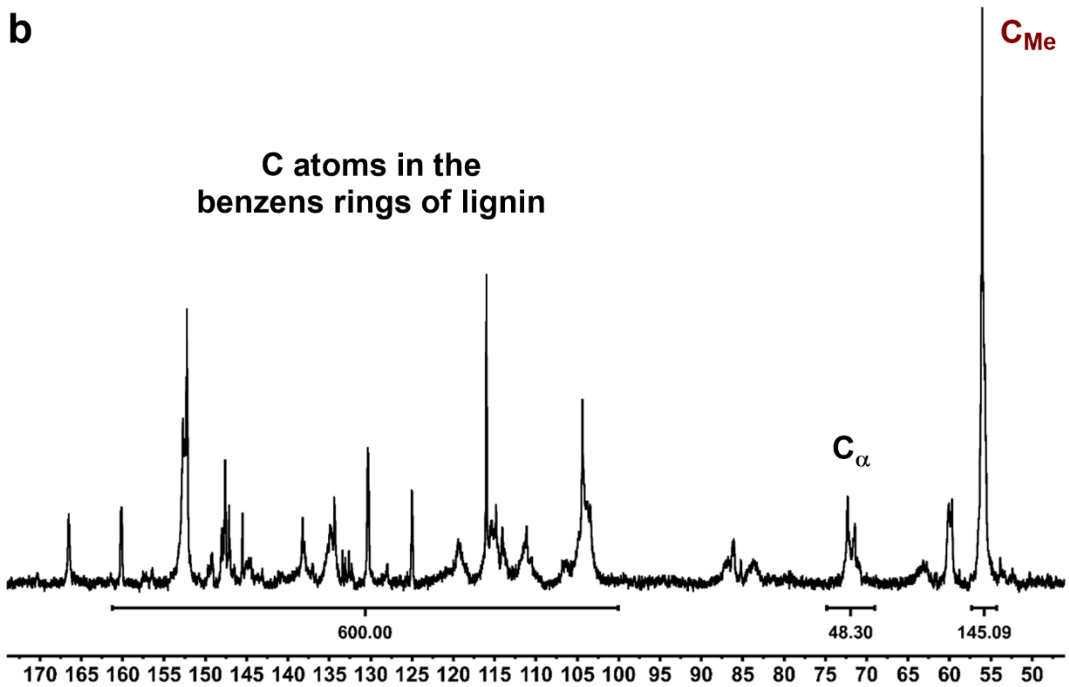

Supplementary Figure 22. $^{1}H/^{13}C$ 2D-HSQC and quantitative ^{13}C NMR spectra of the Phyllostachys pubescens lignin. a $^{1}H/^{13}C$ 2D-HSQC NMR spectra. **b** Quantitative ^{13}C NMR spectra.

(H) p-Hydroxyphenyl unit; (G) guaiacyl unit; (S) syringyl unit; (I) β-aryl ether (β-O-4); (II) resinol (β-β); (III) phenylcoumaran (β-5); C_{α}, the C atom in the phenylpropanol structure; C_{Me}, the C atom in the methoxy group.

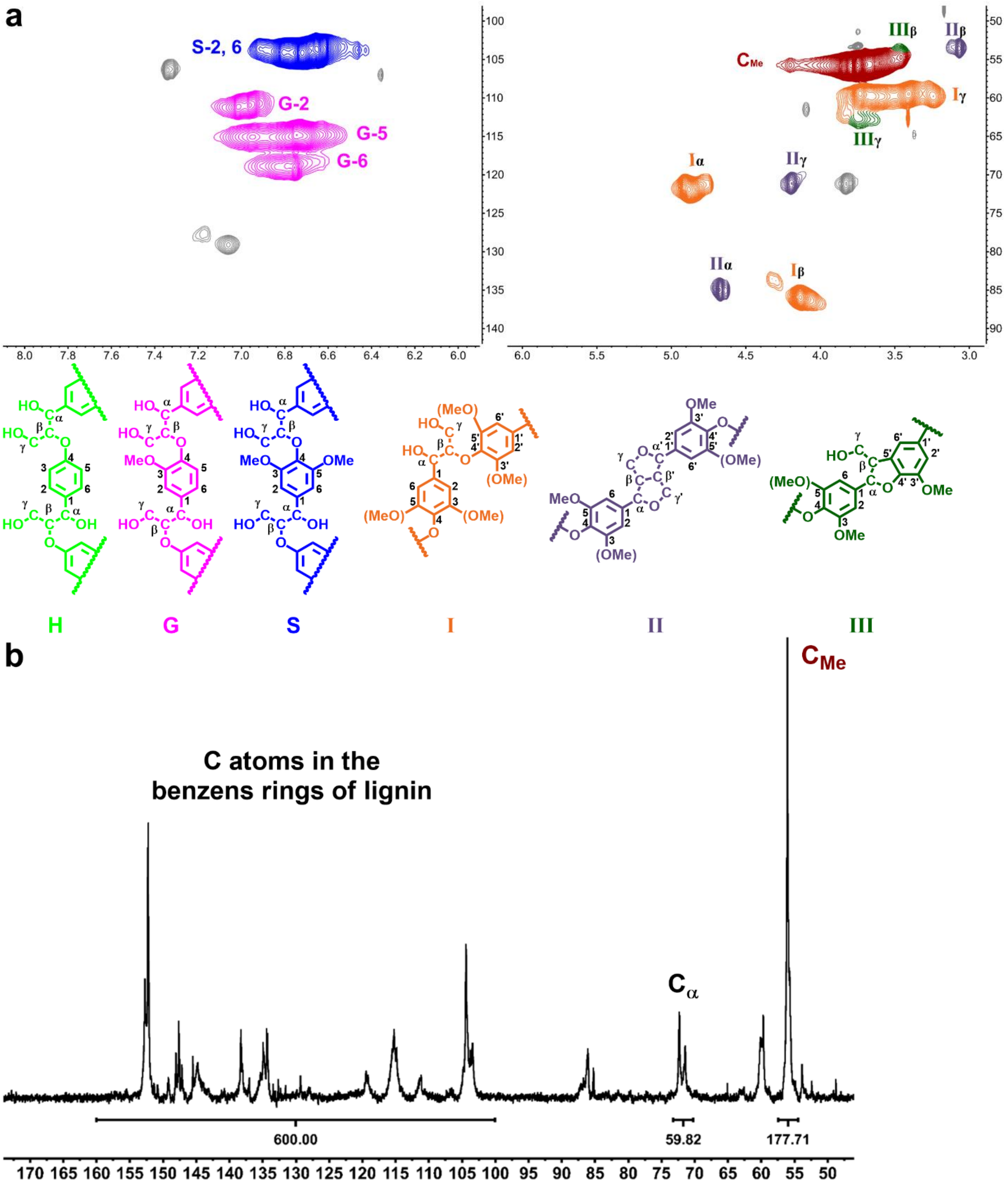

Supplementary Figure 23. ^{1}H/^{13}C 2D-HSQC and quantitative ^{13}C NMR spectra of the applewood lignin. a ^{1}H/^{13}C 2D-HSQC NMR spectra. **b** Quantitative ^{13}C NMR spectra.

(H) p-Hydroxyphenyl unit; (G) guaiacyl unit; (S) syringyl unit; (I) β-aryl ether (β-O-4); (II) resinol (β-β); (III) phenylcoumaran (β-5); $C_{α}$, the C atom in the phenylpropanol structure; C_{Me}, the C atom in the methoxy group.

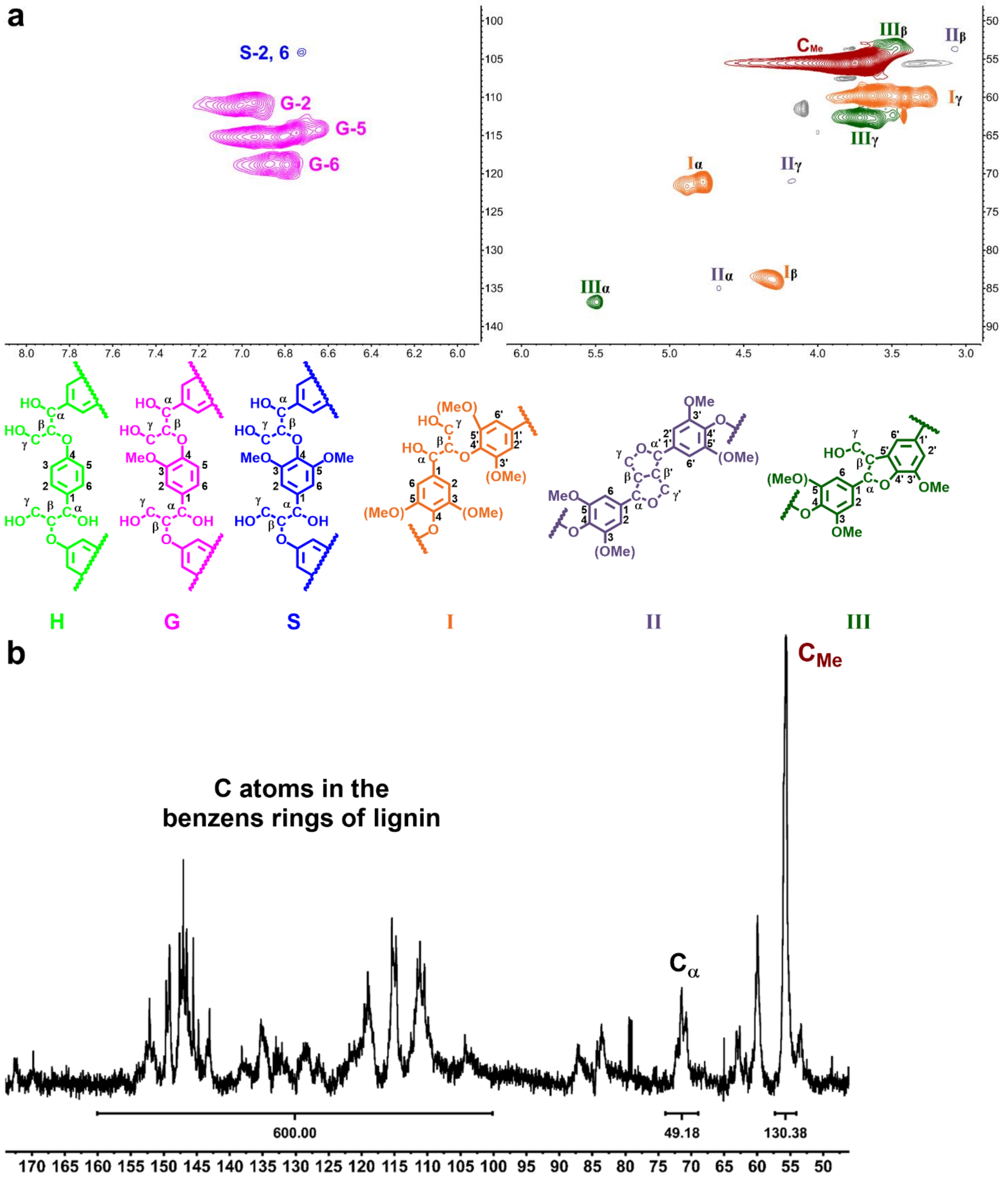

Supplementary Figure 24. $^{1}H/^{13}C$ 2D-HSQC and quantitative ^{13}C NMR spectra of the cedar lignin. a $^{1}H/^{13}C$ 2D-HSQC NMR spectra. **b** Quantitative ^{13}C NMR spectra.

(H) p-Hydroxyphenyl unit; (G) guaiacyl unit; (S) syringyl unit; (I) β-aryl ether (β-O-4); (II) resinol (β-β); (III) phenylcoumaran (β-5); C_{α}, the C atom in the phenylpropanol structure; C_{Me}, the C atom in the methoxy group.

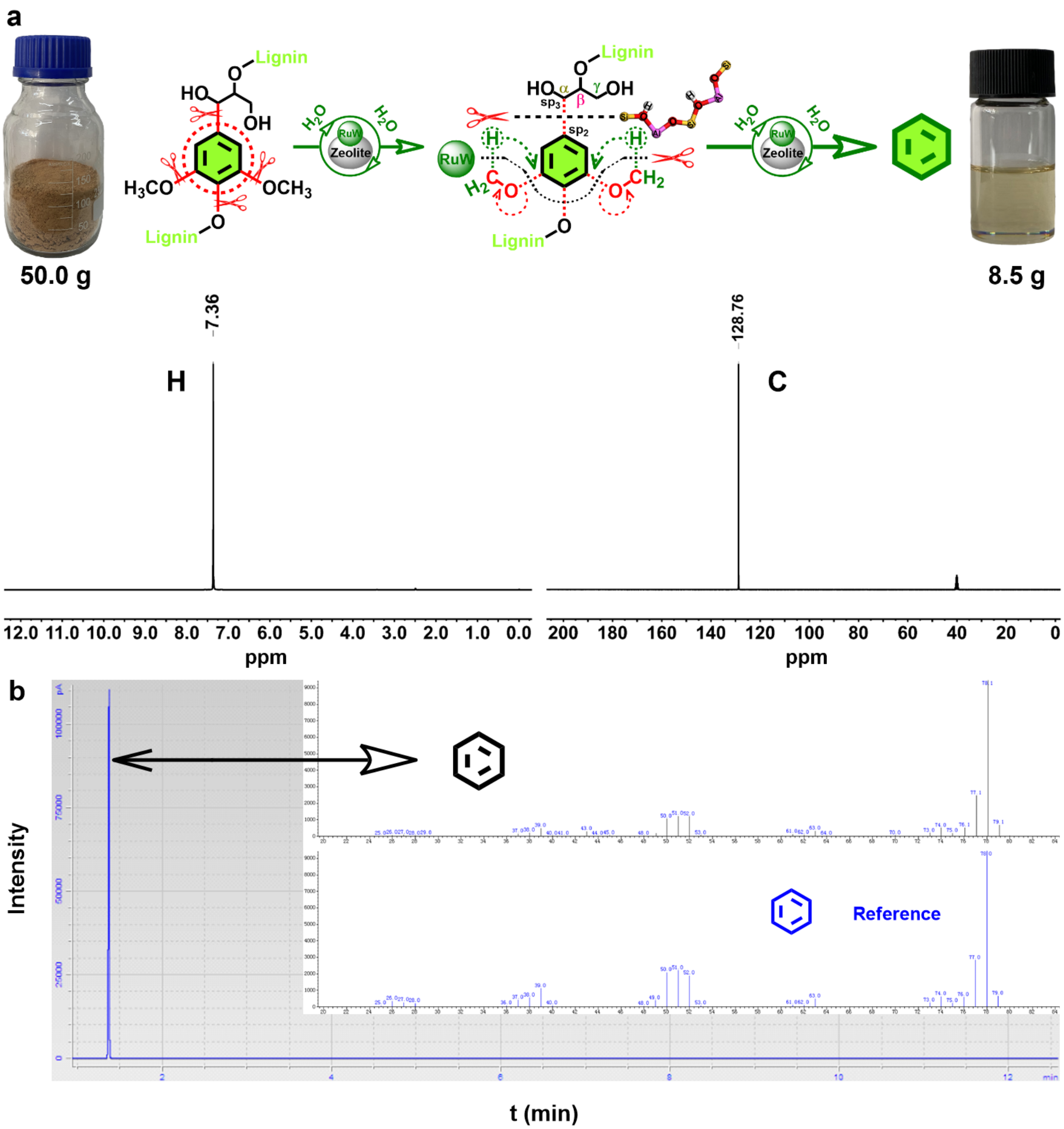

Supplementary Figure 25. Sale-up experiment for the transformation of the pine lignin. a ^{1}H and ^{13}C NMR analyses of the organic layer. **b** GC trace of the organic layer (inset: MS analysis of benzene).

Reaction conditions: lignin (50.0 g), RuW/HY30 (50.0 g, 3.5 wt% Ru, 20 wt% W), H_2O (650 mL), 240 °C, 12 h, 0.1 MPa N_2, 800 rpm. ^{1}H and ^{13}C chemical shifts were referenced to DMSO-d6 at 2.50 and 39.52 ppm.

Supplementary Tables

Supplementary Table 1. Screening of catalysts for in situ refining 1-(4-methoxyphenyl)-1-propanol (1a) over supported non-noble metal catalysts.

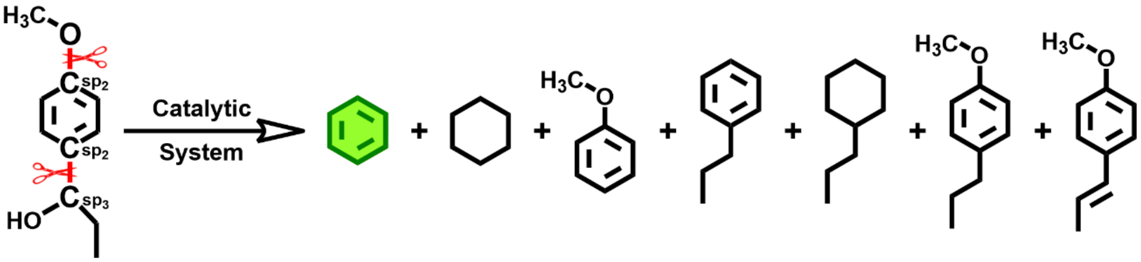

1a **1b** **1c** **1d** **1e** **1f** **1g** **1h**

Entry	Catalytic system[a]		t (h)	Conv. (%)	Select. (%)							Yie. of **1b** (%)
	Catalyst[b]	Gas			**1b**	**1c**	**1d**	**1e**	**1f**	**1g**	**1h**	
1	NiW/HY30	N_2	6.0	99.9	0.0	0.0	99.1	0.0	0.0	0.0	0.0	0.0
2	CoW/HY30	N_2	6.0	99.9	0.0	0.0	99.3	0.0	0.0	0.0	0.0	0.0
3	FeW/HY30	N_2	6.0	99.9	0.0	0.0	99.2	0.0	0.0	0.0	0.0	0.0
4	MoW/HY30	N_2	6.0	99.9	0.0	0.0	99.0	0.0	0.0	0.0	0.0	0.0
5	CuW/HY30	N_2	6.0	99.9	0.0	0.0	99.1	0.0	0.0	0.0	0.0	0.0
6	NiW/HY30	H_2	6.0	99.9	10.6	5.9	49.3	2.5	1.0	20.1	0.0	10.6
7	CoW/HY30	H_2	6.0	99.9	2.9	0.0	76.9	1.5	0.0	18.1	0.0	2.9
8	FeW/HY30	H_2	6.0	99.9	1.8	0.0	81.8	0.6	0.0	15.6	0.0	1.8
9	MoW/HY30	H_2	6.0	99.9	0.0	0.0	96.6	0.0	0.0	3.2	0.0	0.0
10	CuW/HY30	H_2	6.0	99.9	0.0	0.0	99.1	0.0	0.0	0.0	0.0	0.0

[a]Reaction results are the averages of three experiments conducted in parallel. **1a** (1.0 mmol), H_2O (5.0 mL), 180 °C, 0.1 MPa N_2 or 1.0 MPa H_2, 800 rpm.
[b]MW/HY30 catalysts (M: Ni, Co, Fe, Mo and Cu. 0.20 g, 2.5 wt% M, 14 wt% W, the content of metal is based on HY30 zeolite, and determined by ICP).

Supplementary Table 2. Structure parameters of RuW/HY_{30} catalyst extracted from the EXAFS fitting.

Sample	Edge	Path	N[b]	R (Å)[c]	σ^2 (Å^2·10^{-3})[d]	ΔE_0 (eV)[e]	R factor (%)[f]
RuW/HY_{30}[a]	Ru *K*-edge	Ru-W1	3.1	2.68	5.6	-10.2	1.2
		Ru-W2	2.3	3.13	5.6	-10.2	
		Ru-Ru1	3.6	2.66	7.3	-6.9	
		Ru-Ru2	2.7	3.10	7.3	-6.9	
	W L_3-edge	W-Ru1	0.9	2.68	5.6	3.6	1.2
		W-Ru2	0.7	3.13	5.6	3.6	
		W-W1	5.6	2.73	7.3	5.6	
		W-W2	4.2	3.15	7.3	5.6	

[a]RuW/HY_{30} catalyst (2.5 wt% Ru, 14 wt% W).
[b]N: coordination numbers.
[c]R: bond distance.
[d]σ^2: Debye-Waller factors.
[e]ΔE_0: the inner potential correction.
[f]R factor: goodness of fit. S_0^2 were set as 0.90 and 0.86, respectively for Ru-W/Ru-Ru and W-Ru/W-W, which were obtained from the experimental EXAFS fit of Ru foil and W foil references by fixing CN as the known crystallographic value and was fixed to all the samples. Error bounds (accuracies) that characterize the structural parameters obtained by EXAFS spectroscopy were estimated as $N \pm 15\%$; $R \pm 0.02$ Å; $\sigma^2 \pm 5\%$; $\Delta E_0 \pm 20\%$.

Supplementary Table 3. Acid properties of the RuW/HY zeolites.

Catalyst[a]	Quantity (μmol/g) and distribution of acid sites										
	Total acid				Strong acid				Strong/Total[f]		
	B[b]	L[c]	B+L[d]	B/L[e]	B	L	B+L	B/L	B	L	B+L
RuW/HY30	118	19	137	6.21	95	11	106	8.64	80.5	57.9	77.4
RuW/HY3	231	175	406	1.32	128	79	207	1.62	55.4	45.1	51.0
RuW/HY5	225	158	383	1.42	125	73	198	1.71	55.6	46.2	51.7
RuW/HY15	179	75	254	2.39	115	38	153	3.03	64.2	50.7	60.2
RuW/HY40	92	14	106	6.57	75	9	84	8.33	81.5	64.3	79.2

[a]RuW/Zeolite catalysts (2.5 wt% Ru, 14 wt% W).
[b]Bronsted acid site.
[c]Lewis acid site.
[d]Sum of the quantities of Bronsted acid and Lewis acid sites.
[e]Ratio of the quantities of Bronsted acid and Lewis acid sites.
[f]Ratio of the quantities of strong acid and total acid sites, %.

Supplementary Table 4. Textural properties of the catalysts.

Catalyst[a]	Surface Area ($m^2 \cdot g^{-1}$)			Volume ($cm^3 \cdot g^{-1}$)		
	S_{micro}[b]	S_{meso}[c]	R_S[d]	V_{micro}[e]	V_{meso}[f]	R_V[g]
RuW/HY_{30}	153	268	1.75	0.075	0.218	2.91
RuW/HY_3	291	85	0.29	0.151	0.079	0.52
RuW/HY_5	279	99	0.35	0.143	0.088	0.62
RuW/HY_{15}	243	139	0.57	0.115	0.125	1.09
RuW/HY_{40}	150	275	1.83	0.070	0.225	3.21
RuW/Beta[h]	193	118	0.61	0.085	0.088	1.04
RuW/HZSM-5_{14}	97	56	0.58	0.043	0.042	0.98
RuW/HZSM-5_{18}	106	65	0.61	0.049	0.052	1.06
RuW/HZSM-5_{30}	112	69	0.62	0.052	0.058	1.12
RuW/HZSM-5_{35}	123	75	0.61	0.057	0.066	1.16
RuW/HZSM-5_{65}	141	63	0.45	0.067	0.050	0.75
RuW/HZSM-5_{235}	163	53	0.33	0.079	0.038	0.48
RuW/Mordenite[i]	157	65	0.41	0.077	0.051	0.66
RuW/SAPO[j]	90	37	0.41	0.040	0.029	0.73
RuW/MCM-41[k]	—	614	—	—	0.380	—

[a]RuW/Zeolite catalysts (2.5 wt% Ru, 14 wt% W).
[b]The t-plot micropore surface area.
[c]The t-plot mesopore surface area.
[d]Ratio of the mesopore surface area and micropore surface area.
[e]The t-plot micropore volume.
[f]BJH mesopore volume.
[g]Ratio of the mesopore volumn and micropore volumn.
[h]Hydrogen-type Beta, Si/Al ratio is 25.
[i]Hydrogen-type Mordenite, Si/Al ratio is 10.
[j]Hydrogen-type SAPO-34, Si/Al ratios is 0.25.
[k]All-silica MCM-41.

Supplementary Table 5. Reaction of 1-phenylpropan-1-ol in formaldehyde solution over supported metal catalysts.

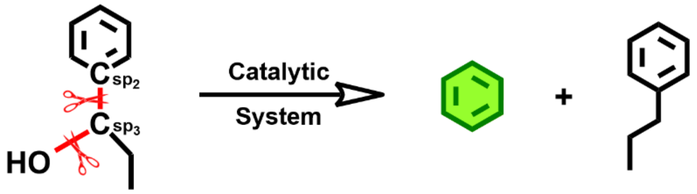

Entry	Catalytic system[a]		t (h)	Conversion (%)	Selectivity (%)		Yield of benzene (%)
	Catalyst[b]	Gas			Benzene	Propylbenzene	
1[c]	RuW/SiO_2	N_2	5.5	99.9	0.0	99.6	0.0
2[d]	RuW/Al_2O_3	N_2	5.5	99.9	0.0	93.2	0.0
3	RuW/HY_3	N_2	5.5	99.9	10.1	82.5	10.1
4	RuW/HY_5	N_2	5.5	99.9	12.8	80.7	12.8
5	RuW/HY_{15}	N_2	5.5	99.9	31.0	65.6	31.0
6[e]	RuW/Beta	N_2	5.5	99.9	9.5	82.6	9.5
7	RuW/HZSM-5_{14}	N_2	5.5	99.9	8.2	83.3	8.2
8	RuW/HZSM-5_{18}	N_2	5.5	99.9	7.5	86.6	7.5
9	RuW/HZSM-5_{30}	N_2	5.5	99.9	7.2	85.2	7.2
10	RuW/HZSM-5_{35}	N_2	5.5	99.9	6.0	90.8	6.0
11	RuW/HZSM-5_{65}	N_2	5.5	99.9	4.9	93.9	4.9
12	RuW/HZSM-5_{235}	N_2	5.5	99.9	1.2	97.9	1.2
13[f]	RuW/Mordenite	N_2	5.5	99.9	0.0	91.0	0.0
14[g]	RuW/SAPO	N_2	5.5	99.9	0.0	95.8	0.0
15[h]	RuW/MCM-41	N_2	5.5	99.9	0.0	99.5	0.0

[a]Reaction results are the averages of three experiments conducted in parallel. **1a** (1.0 mmol), paraformaldehyde (30 mg), H_2O (5.0 mL), 180 °C, 0.1 MPa N_2, 800 rpm. Thermolabile paraformaldehyde is used as the source of formaldehyde to simulate the gradual supply of the formaldehyde generated from the SSH reaction.
[b]RuW/SiO_2, RuW/Al_2O_3 and RuW/zeolite catalysts (0.20 g, 2.5 wt% Ru, 14 wt% W, the content of metal is based on zeolite, SiO_2 and Al_2O_3 materials, and determined by ICP).
[c]Amorphous SiO_2.
[d]γ-Al_2O_3.
[e]Hydrogen-type Beta, Si/Al ratio is 25.
[f]Hydrogen-type Mordenite, Si/Al ratio is 10.
[g]Hydrogen-type SAPO-34, Si/Al ratios is 0.25.
[h]All-silicon MCM-41.

Supplementary Table 6. ^{13}C NMR chemical shifts of the model compounds.

Model compounds	C_{α}[a]	C_{β}[b]	C_{γ}[c]	C_{Me}[d]
1-(4-methoxyphenyl)-1-propanol (**1a**)	73.8	32.6	10.6	55.4
2-(4-methoxyphenyl)propan-2-ol	70.8	32.5	—	55.4
2-(4'-methoxyphenyl)propane	33.1	24.5	—	55.4
1-methoxy-4-propylbenzene	36.9	24.8	14.0	55.4

[a]C_{α}, the C atom at the aliphatic α-C position.
[b]C_{β}, the C atom at the aliphatic β-C position.
[c]C_{γ}, the C atom at the aliphatic γ-C position.
[d]C_{Me}, the C atom in the methoxy group.

Supplementary Table 7. Solid-state 2D $^{13}C\{^{1}H\}$ HETCOR NMR chemical shifts of the intermediates in the reaction of 1-(4-methoxyphenyl)-1-propanol (1a).

Samples	C_{α}[c]	C_{β}[d]	$C_{\beta1}$[e]	$C_{\beta2}$[f]	$C_{\beta3}$[g]	C_{γ}[h]	C_{Me}[i]
1-(4-methoxyphenyl)-1-propanol (**1a**)[a]	38.0{3.1}	—	25.3{1.4}	21.8{1.8}	31.0{3.0}	13.3{0.8}	55.2{3.6}
1-(4-methoxyphenyl)-1-propanol (**1a**)[b]	73.8{4.4}	32.4{1.6}	—	—	—	10.5{0.8}	55.3{3.7}
2-(4-methoxyphenyl)propan-2-ol[b]	—‡	32.2{1.4}	—	—	—	—	55.3{3.7}
2-(4'-methoxyphenyl)propane[b]	33.0{2.8}	23.9{1.2}	—	—	—	—	55.3{3.7}
1-methoxy-4-propylbenzene[b]	36.5{2.4}	24.3{1.5}	—	—	—	13.9{0.9}	55.3{3.7}

[a]$^{13}C\{^{1}H\}$ HETCOR NMR chemical shifts of the intermediates in the reaction of 1-(4-methoxyphenyl)-1-propanol (**1a**). Reaction conditions: **1a** (1 mmol), RuW/HY_{30} (0.20 g, 2.5 wt% Ru, 14 wt% W), H_2O (5.0 ml), 180 ºC, 10 min, 0.1 MPa N_2, 800 rpm.
[b]$^{13}C/^{1}H$ 2D HSQC NMR chemical shifts of the model compounds.
[c]C_{α}, the C atom at the aliphatic α-C position. No signal for the Catom without H atom at C_{α} position of 2-(4-methoxyphenyl)propan-2-ol.
[d]C_{β}, the C atom at the aliphatic β-C position of the model compounds.
[e]$C_{\beta1}$, the C atom at the aliphatic β1-C position of the intermediates in the reaction of **1a**.
[f]$C_{\beta2}$, the C atom at the aliphatic β2-C position of the intermediates in the reaction of **1a**.
[g]$C_{\beta3}$, the C atom at the aliphatic β3-C position of the intermediates in the reaction of **1a**.
[h]C_{γ}, the C atom at the aliphatic γ-C position.
[i]C_{Me}, the C atom in the methoxy group.

Supplementary Table 8. Reaction of 4-(1-hydroxypropyl)phenol in formaldehyde solution over the RuW/HY$_{30}$ catalyst.

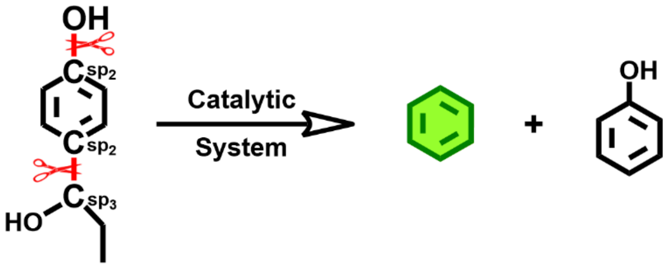

Entry	Catalytic system[a]		t (h)	Conversion (%)	Selectivity (%)		Yield of Benzene (%)
	Catalyst[b]	M_{PF}[c] (mg)			Benzene	Phenol	
1	RuW/HY$_{30}$	0.0	5.5	99.9	0.0	99.9	0.0
2	RuW/HY$_{30}$	0.3	5.5	99.9	0.9	99.0	0.9
3	RuW/HY$_{30}$	0.6	5.5	99.9	2.2	97.7	2.2
4	RuW/HY$_{30}$	0.9	5.5	99.9	3.1	96.8	3.1
5	RuW/HY$_{30}$	1.5	5.5	99.9	6.3	93.6	6.3
6	RuW/HY$_{30}$	2.4	5.5	99.9	10.8	89.1	10.8
7	RuW/HY$_{30}$	3.6	5.5	99.9	15.6	84.3	15.6
8	RuW/HY$_{30}$	4.5	5.5	99.9	20.1	79.8	20.1

[a]Reaction results are the averages of three experiments conducted in parallel. 4-(1-hydroxypropyl)phenol (1.0 mmol), H_2O (5.0 mL), 180 °C, 0.1 MPa N_2, 800 rpm.
[b]RuW/HY$_{30}$ catalyst (0.20 g, 2.5 wt% Ru, 14 wt% W, the content of metal is based on HY$_{30}$ zeolite, and determined by ICP).
[c]m_{PF}, the mass of paraformaldehyde in the reaction system.

Supplementary Table 9. Yields of benzene product in the refining of lignins.

Lignin	Content of phenylpropanol structure (mol%)[a]	Methoxy group/benzene ring ratio[b]	Yield of benzene (wt%)[c]
Pine	55.6	1.56	18.8
Cedrela	57.1	1.58	17.5
Poplar	54.5	1.50	15.6
Willow	53.9	1.51	14.5
Eucalyptus	59.1	1.59	13.1
Peach	54.3	1.73	12.8
Phyllostachys pubescens	48.3	1.45	11.9
Applewood	59.8	1.78	11.1
Cedar	49.2	1.30	10.5

[a]Phenylpropanol structure, [-$(H_3CO)_n$Ph-C_αH(OH)-, n=0, 1 and 2], the content of the defined phenylpropanol structure in lignin is calculated based on the quantitative analysis of the C_α atom (Supplementary Figures 15, 17 to 24), using Eq. (2) as follows:

$$\text{Content of phenylpropanol structure} = \frac{\text{Quantity of } C_\alpha \text{ atoms in the phenylpropanol structures}}{\text{Quantity of C atoms in the benzene rings}} \times 6 \times 100\%$$

Eq. (2).

[b]Methoxy group/benzene ring ratio, the ratio of the quantity of methoxy group substituted on the benzene ring to the quantity of benzene ring in lignin. The ratio is calculated based on the quantitative analysis of the C atom in the methoxy group (Supplementary Figures 15, 17 to 24), using Eq. (3) as follows:

$$\text{Methoxy group/benzene ring ratio} = \frac{\text{Quantity of C atoms in the methoxy groups}}{\text{Quantity of C atoms in the benzene rings}} \times 6 \quad \text{Eq. (3).}$$

[c]Yields of benzene product provided are the averages of three experiments conducted in parallel. Reaction conditions: lignin (0.50 g), RuW/HY_{30} (0.50 g, 3.5 wt% Ru, 20 wt% W), H_2O (6.5 ml), 240 ºC, 12 h, 0.1 MPa N_2, 800 rpm.

Supplementary NMR spectra

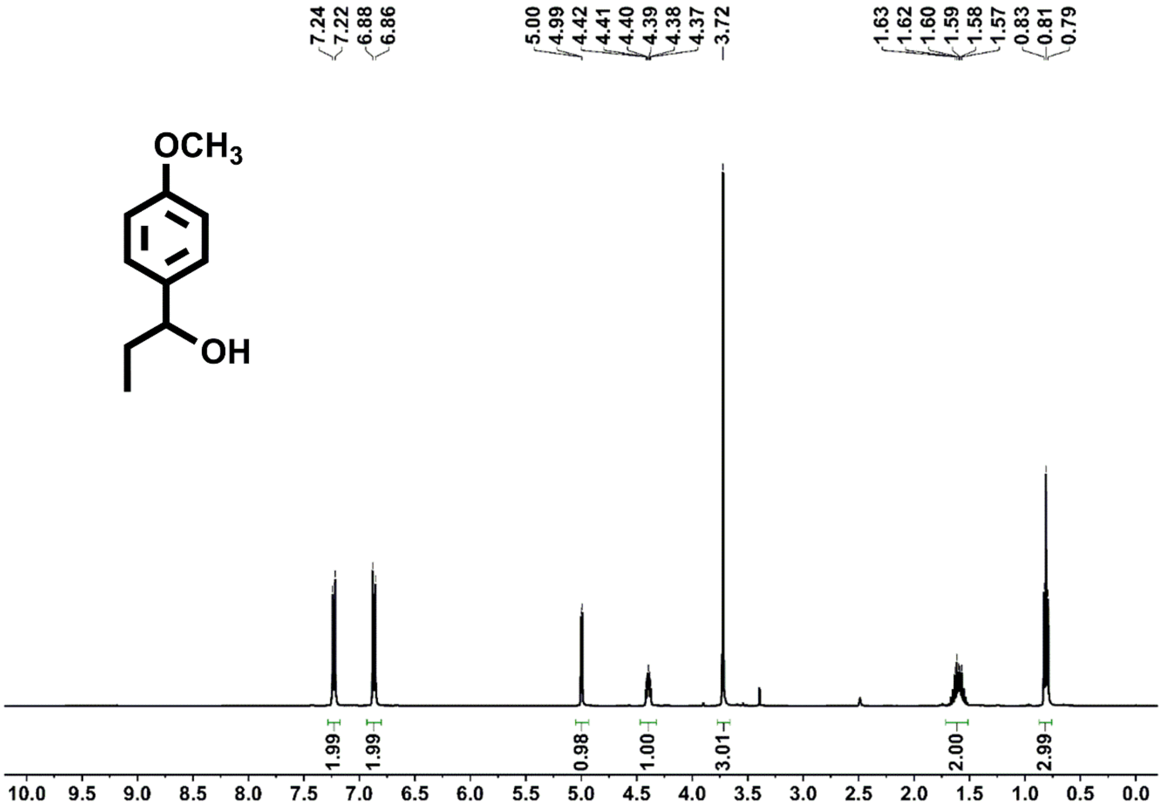

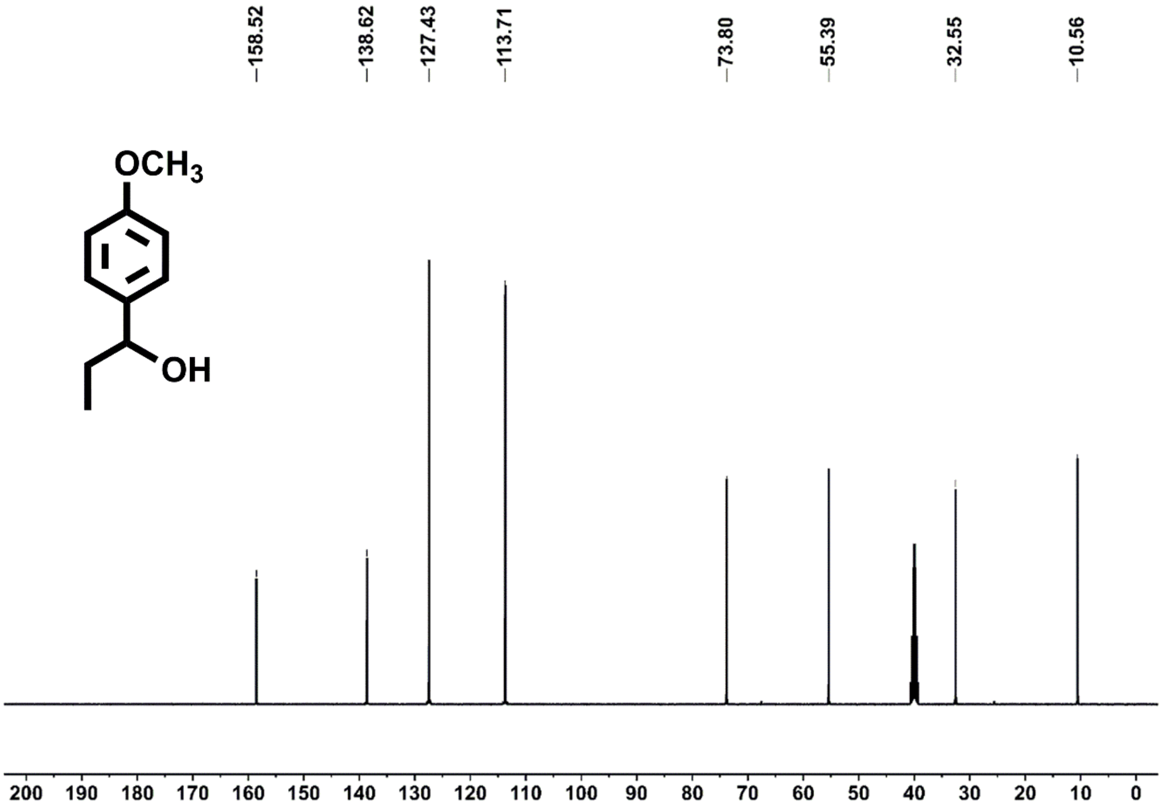

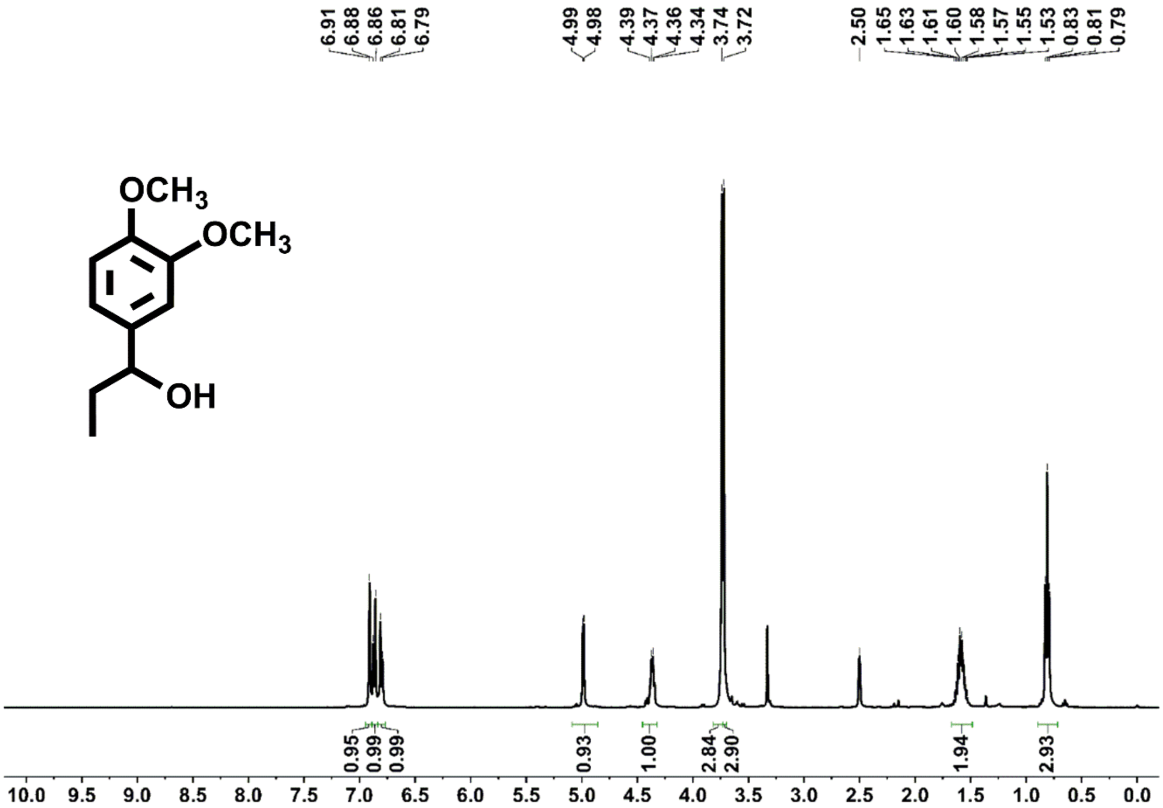
6.91
6.88
6.86
6.81
6.79
4.99
4.98
4.39
4.37
4.36
4.34
3.74
3.72
2.50
1.65
1.63
1.61
1.60
1.58
1.57
1.55
1.53
0.83
0.81
0.79
OCH3
OCH3
OH
0.95
0.99
0.99
0.93
1.00
2.84
2.90
1.94
2.93
10.0 9.5 9.0 8.5 8.0 7.5 7.0 6.5 6.0 5.5 5.0 4.5 4.0 3.5 3.0 2.5 2.0 1.5 1.0 0.5 0.0

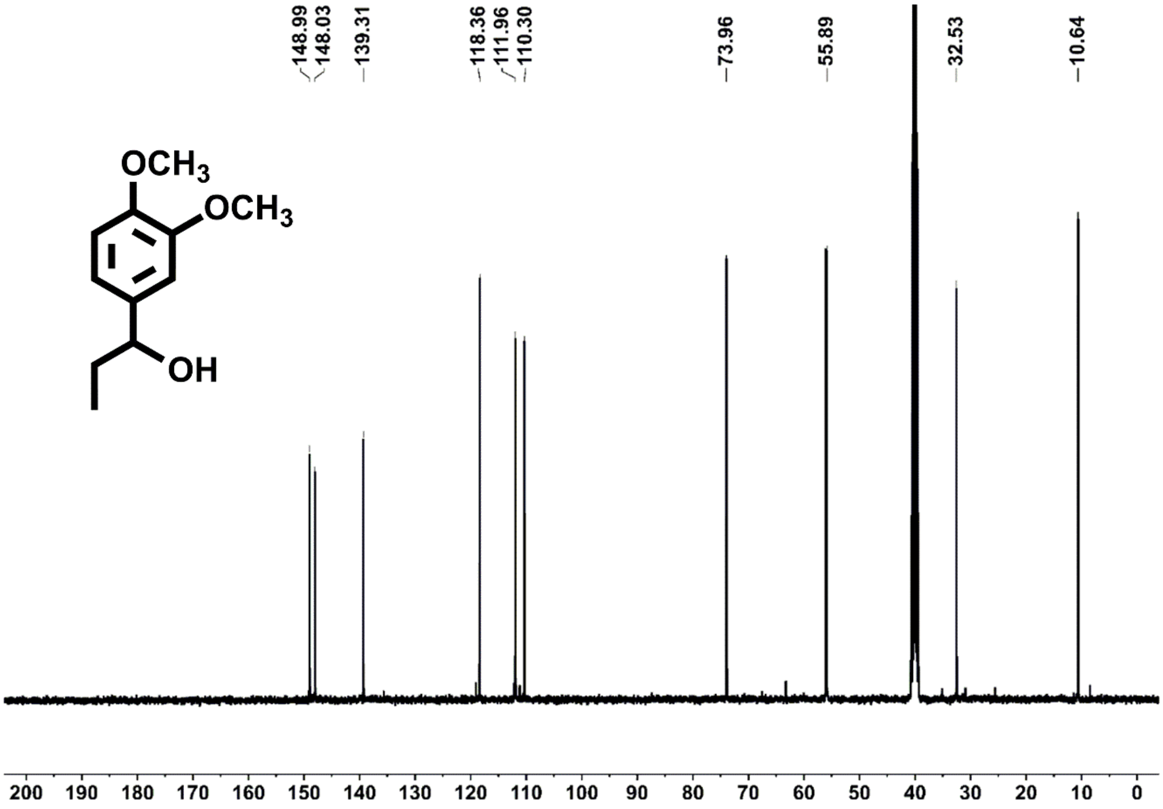
148.99
148.03
139.31
118.36
111.96
110.30
73.96
55.89
32.53
10.64
OCH3
OCH3
OH
200 190 180 170 160 150 140 130 120 110 100 90 80 70 60 50 40 30 20 10 0

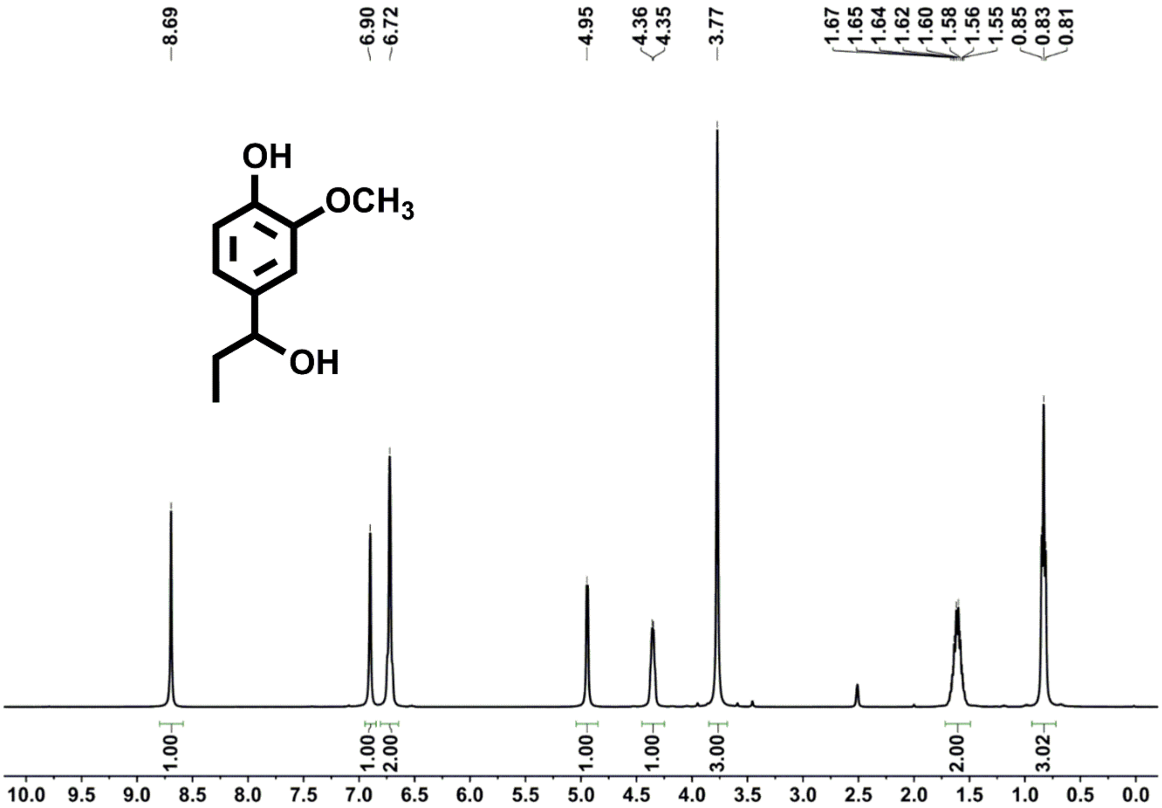
8.69
6.90
6.72
4.95
4.36
4.35
3.77
1.67
1.65
1.64
1.62
1.60
1.58
1.56
1.55
0.85
0.83
0.81
OH
OCH3
OH
1.00
1.00
2.00
1.00
1.00
3.00
2.00
3.02
10.0 9.5 9.0 8.5 8.0 7.5 7.0 6.5 6.0 5.5 5.0 4.5 4.0 3.5 3.0 2.5 2.0 1.5 1.0 0.5 0.0

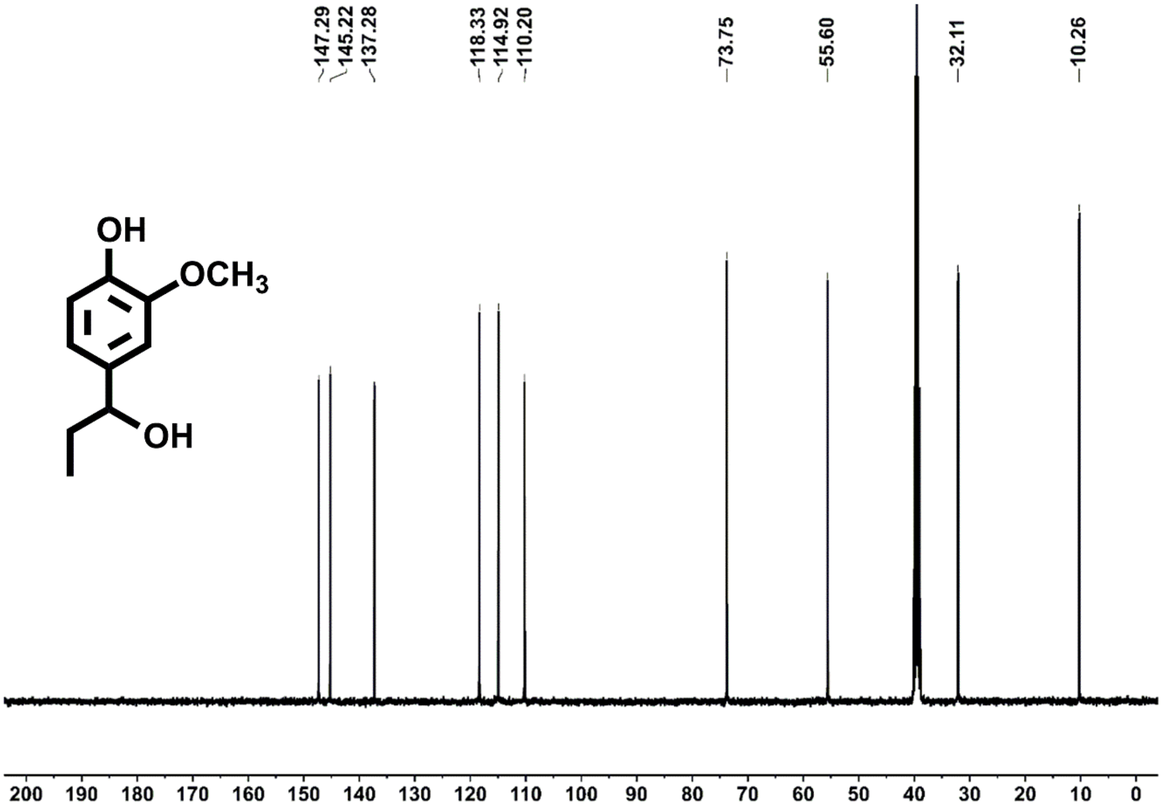
147.29
145.22
137.28
118.33
114.92
110.20
73.75
55.60
32.11
10.26
OH
OCH3
OH
200 190 180 170 160 150 140 130 120 110 100 90 80 70 60 50 40 30 20 10 0

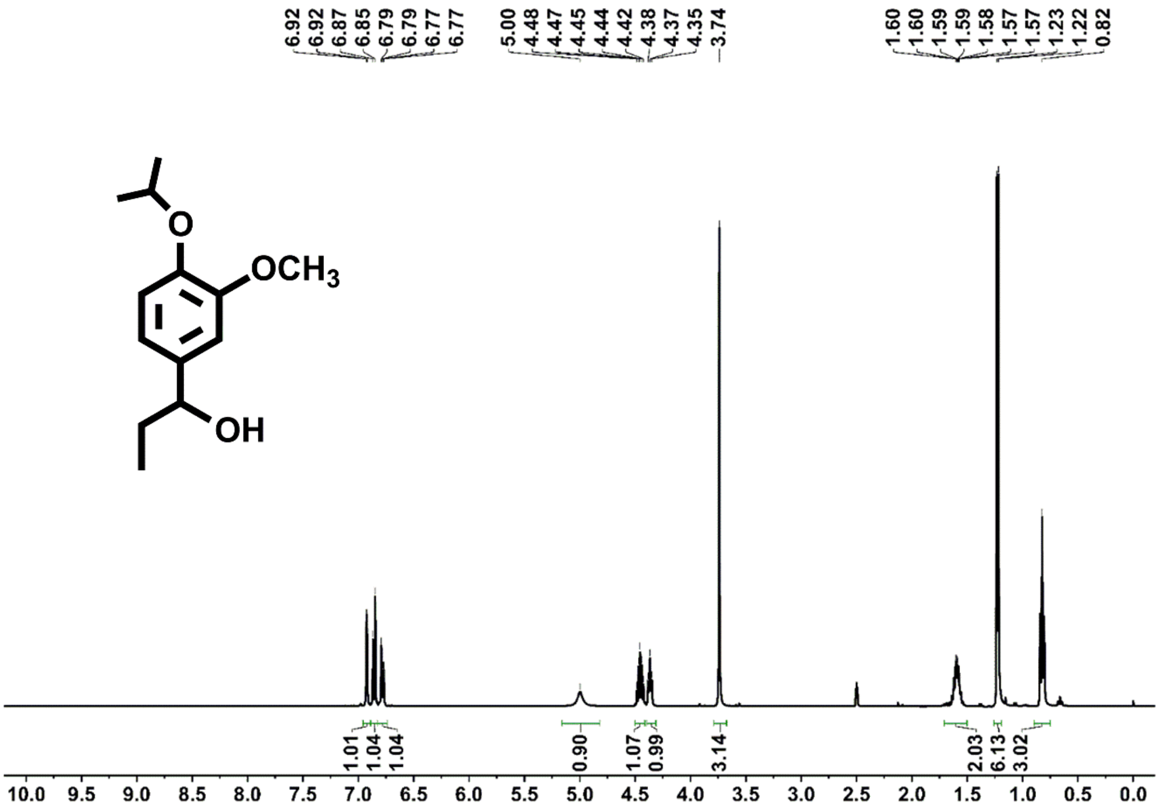
6.92 6.92 6.87 6.85 6.79 6.79 6.77 6.77
5.00 4.48 4.47 4.45 4.44 4.42 4.38 4.37 4.35 3.74
1.60 1.60 1.59 1.59 1.58 1.57 1.57 1.23 1.22 0.82
O
OCH_3
OH
1.01 1.04 1.04 0.90 1.07 0.99 3.14 2.03 6.13 3.02
10.0 9.5 9.0 8.5 8.0 7.5 7.0 6.5 6.0 5.5 5.0 4.5 4.0 3.5 3.0 2.5 2.0 1.5 1.0 0.5 0.0

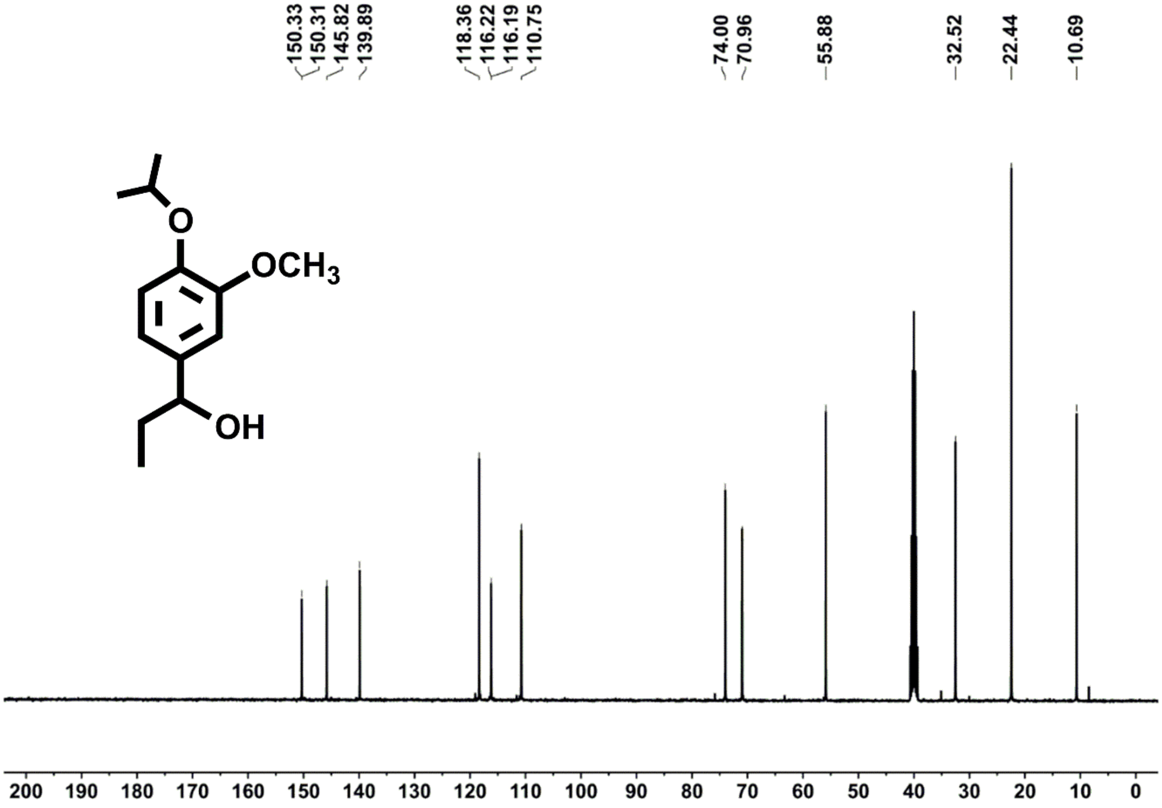
150.33 150.31 145.82 139.89
118.36 116.22 116.19 110.75
74.00 70.96
55.88
32.52
22.44
10.69
O
OCH_3
OH
200 190 180 170 160 150 140 130 120 110 100 90 80 70 60 50 40 30 20 10 0

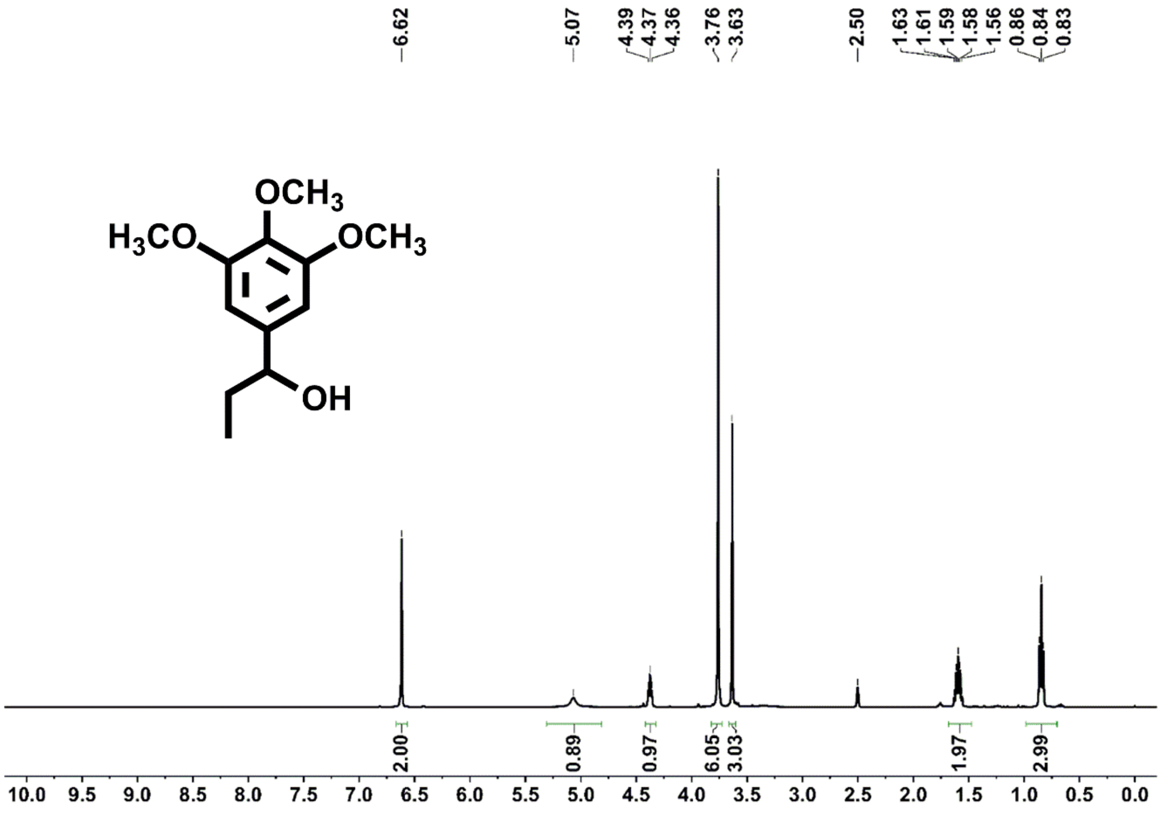
6.62
5.07
4.39
4.37
4.36
3.76
3.63
2.50
1.63
1.61
1.59
1.58
1.56
0.86
0.84
0.83
OCH_3
H_3CO
OCH_3
OH
2.00
0.89
0.97
6.05
3.03
1.97
2.99
10.0 9.5 9.0 8.5 8.0 7.5 7.0 6.5 6.0 5.5 5.0 4.5 4.0 3.5 3.0 2.5 2.0 1.5 1.0 0.5 0.0

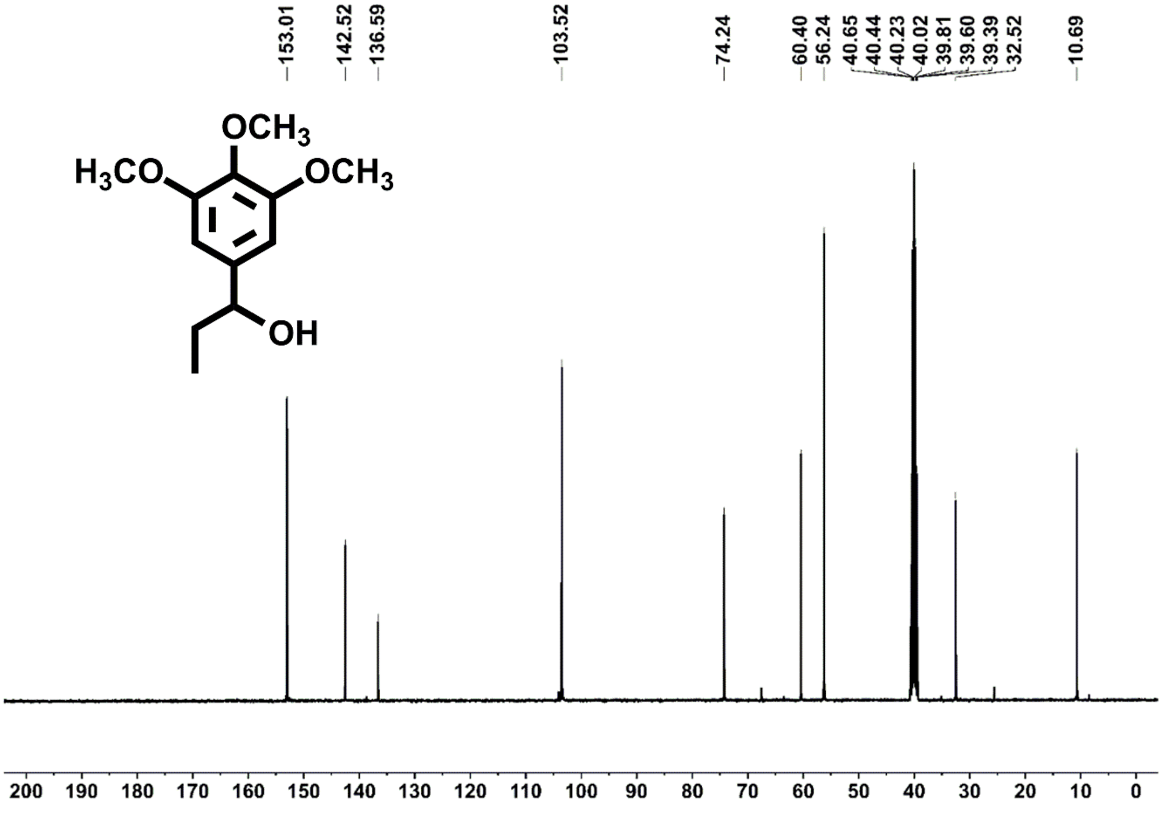
153.01
142.52
136.59
103.52
74.24
60.40
56.24
40.65
40.44
40.23
40.02
39.81
39.60
39.39
32.52
10.69
OCH_3
H_3CO
OCH_3
OH
200 190 180 170 160 150 140 130 120 110 100 90 80 70 60 50 40 30 20 10 0

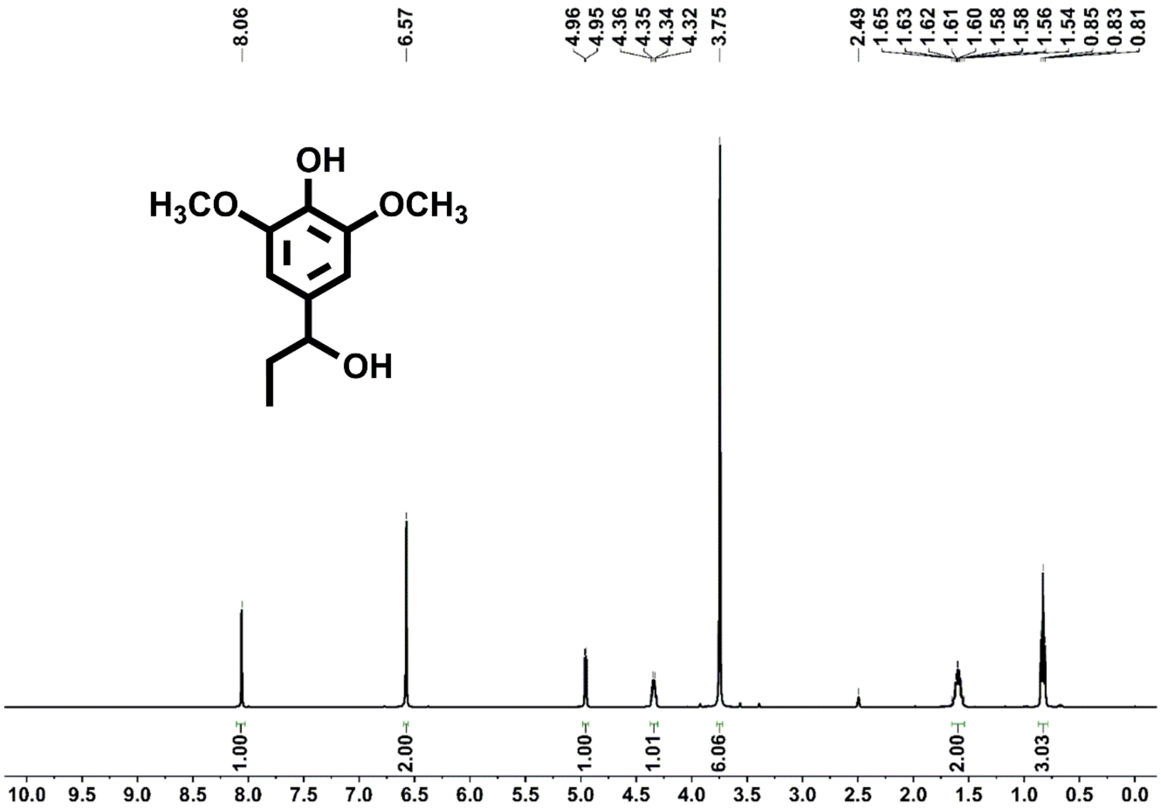
8.06
6.57
4.96
4.95
4.36
4.35
4.34
4.32
3.75
2.49
1.65
1.63
1.62
1.61
1.60
1.58
1.58
1.56
1.54
0.85
0.83
0.81
OH
H_3CO
OCH_3
OH
1.00
2.00
1.00
1.01
6.06
2.00
3.03
10.0 9.5 9.0 8.5 8.0 7.5 7.0 6.5 6.0 5.5 5.0 4.5 4.0 3.5 3.0 2.5 2.0 1.5 1.0 0.5 0.0

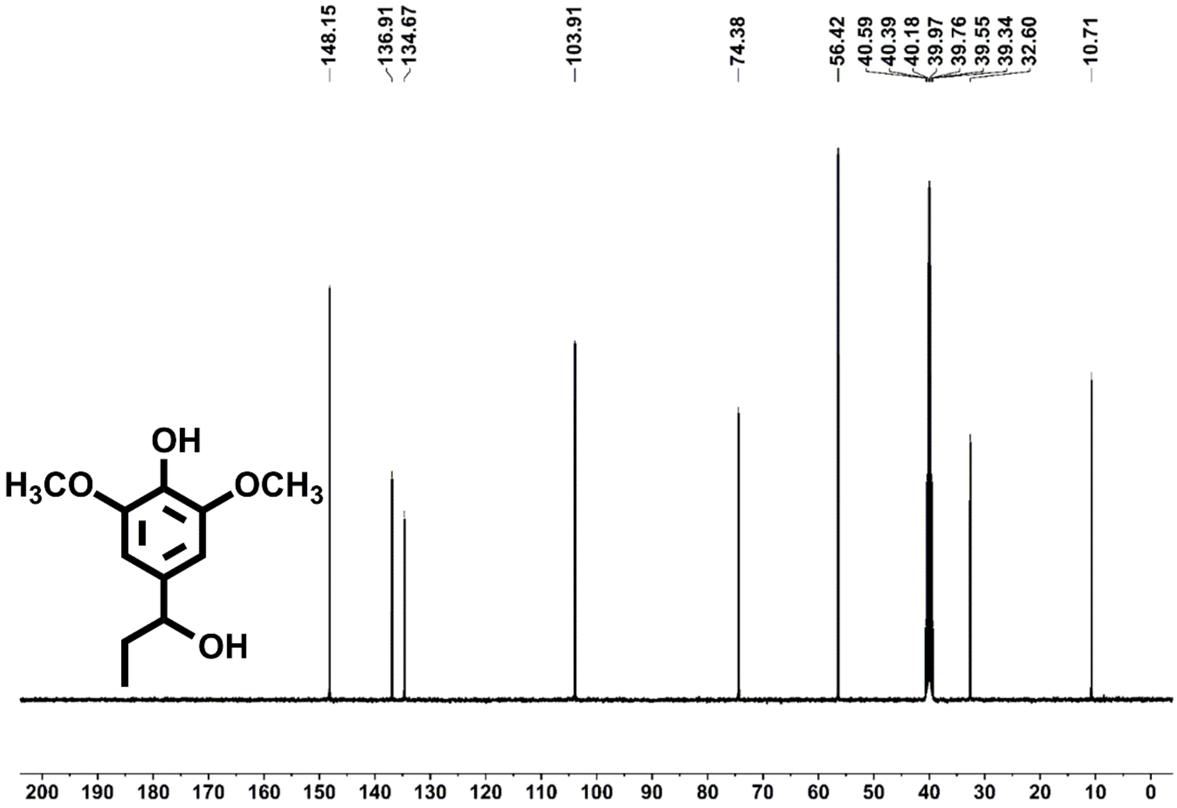
148.15
136.91
134.67
103.91
74.38
56.42
40.59
40.39
40.18
39.97
39.76
39.55
39.34
32.60
10.71
OH
H_3CO
OCH_3
OH
200 190 180 170 160 150 140 130 120 110 100 90 80 70 60 50 40 30 20 10 0

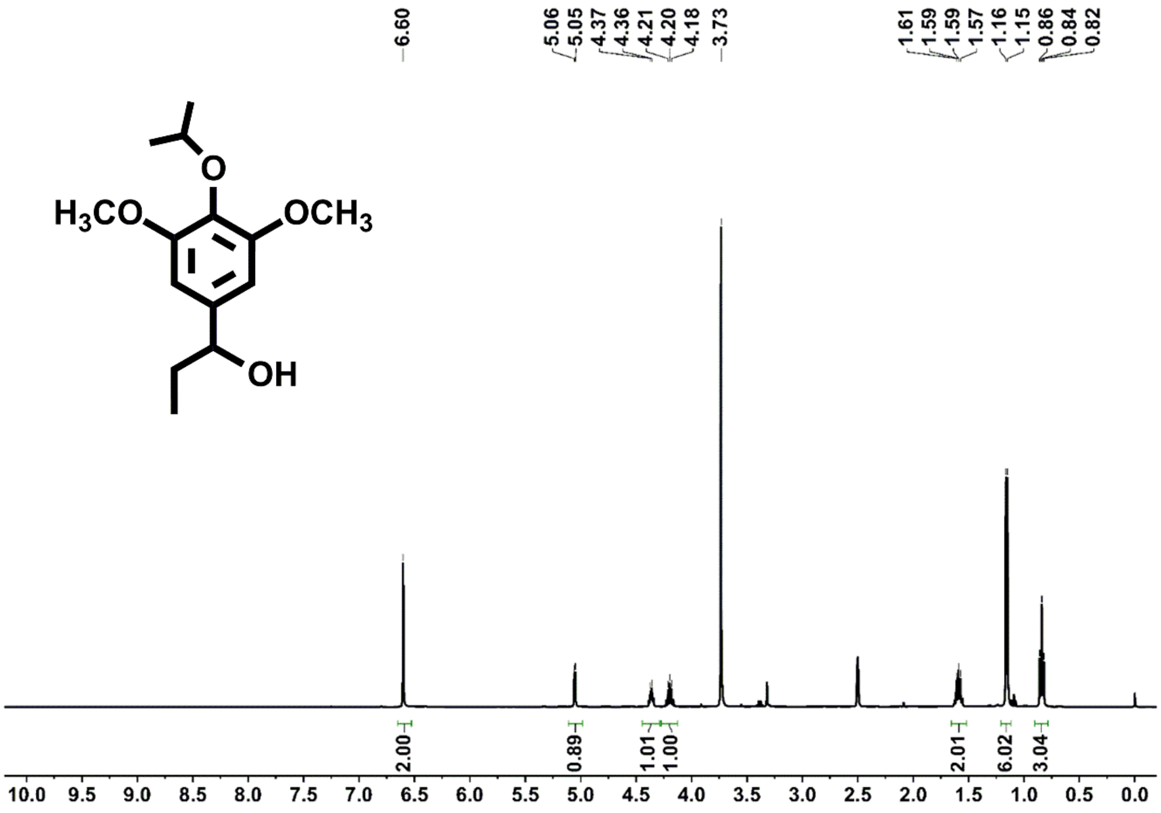

6.60
5.06
5.05
4.37
4.36
4.21
4.20
4.18
3.73
1.61
1.59
1.59
1.57
1.16
1.15
0.86
0.84
0.82
H_3CO
OCH_3
O
OH
2.00
0.89
1.01
1.00
2.01
6.02
3.04
10.0 9.5 9.0 8.5 8.0 7.5 7.0 6.5 6.0 5.5 5.0 4.5 4.0 3.5 3.0 2.5 2.0 1.5 1.0 0.5 0.0

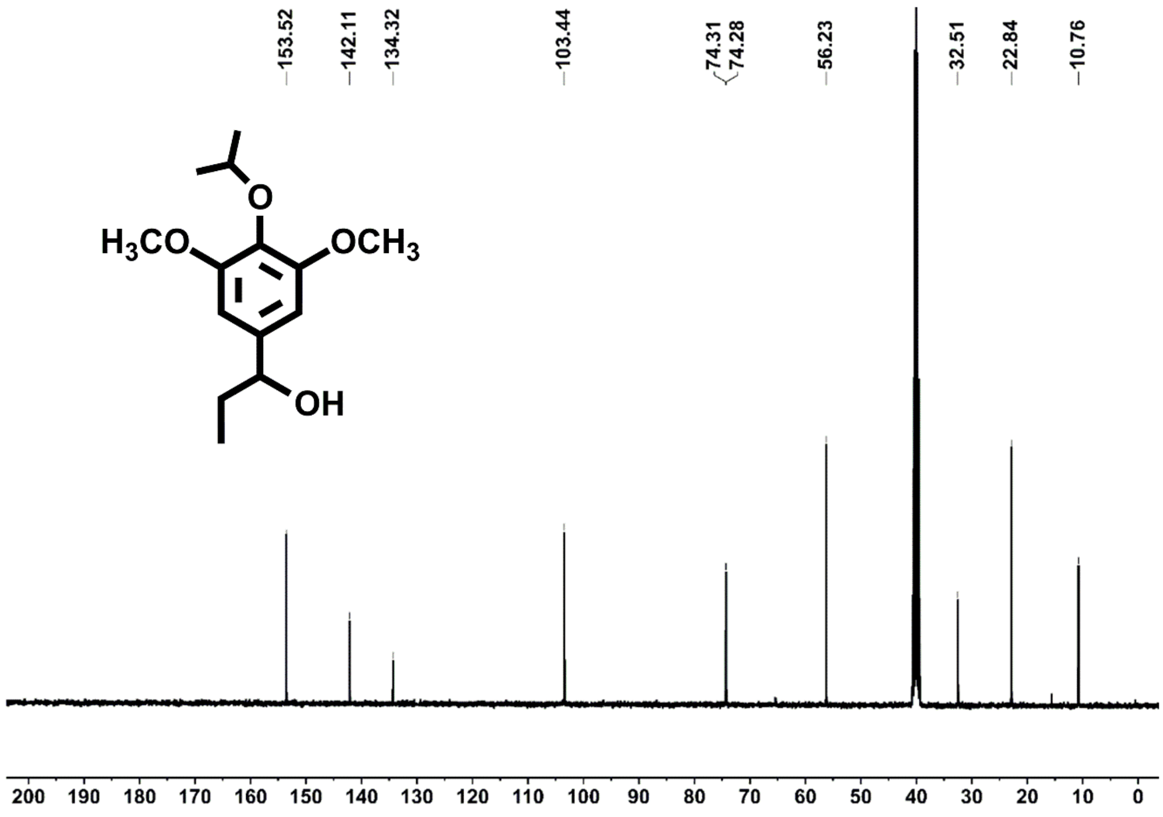

153.52
142.11
134.32
103.44
74.31
74.28
56.23
32.51
22.84
10.76
H_3CO
OCH_3
O
OH
200 190 180 170 160 150 140 130 120 110 100 90 80 70 60 50 40 30 20 10 0

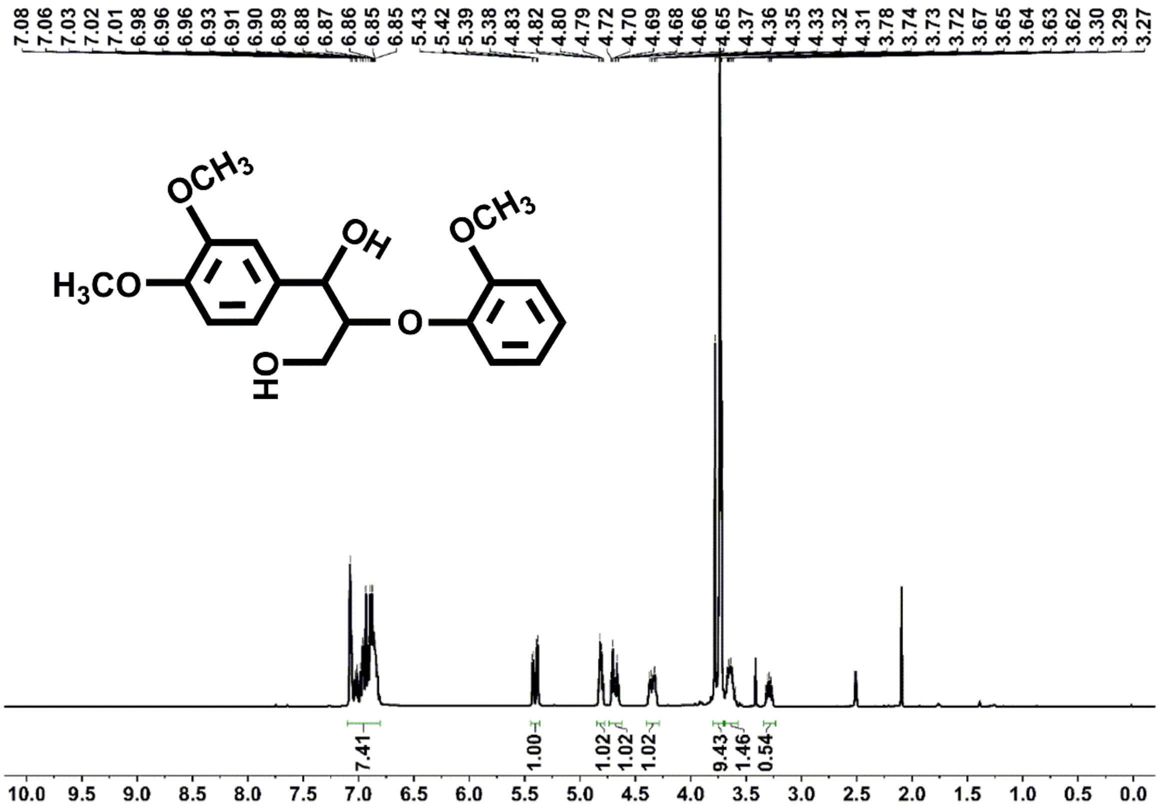
7.08 7.06 7.03 7.02 7.01 6.98 6.96 6.96 6.93 6.91 6.90 6.89 6.88 6.87 6.86 6.85 6.85 5.43 5.42 5.39 5.38 4.83 4.82 4.80 4.79 4.72 4.70 4.69 4.68 4.66 4.65 4.37 4.36 4.35 4.33 4.32 4.31 3.78 3.74 3.73 3.72 3.67 3.65 3.64 3.63 3.62 3.30 3.29 3.27
OCH3
OH
OCH3
H3CO
O
HO
7.41
1.00
1.02
1.02
1.02
9.43
1.46
0.54
10.0 9.5 9.0 8.5 8.0 7.5 7.0 6.5 6.0 5.5 5.0 4.5 4.0 3.5 3.0 2.5 2.0 1.5 1.0 0.5 0.0

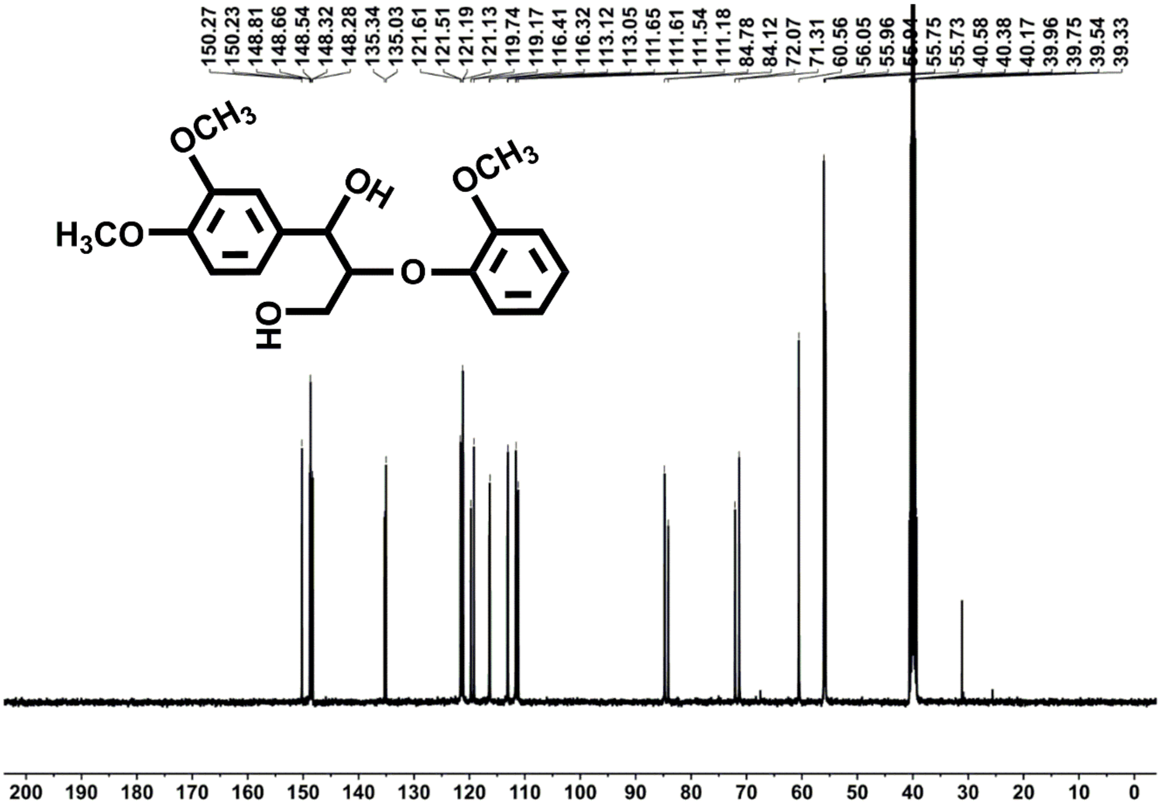
150.27 150.23 148.81 148.66 148.54 148.32 148.28 135.34 135.03 121.61 121.51 121.19 121.13 119.74 119.17 116.41 116.32 113.12 113.05 111.65 111.61 111.54 111.18 84.78 84.12 72.07 71.31 60.56 56.05 55.96 55.94 55.75 55.73 40.58 40.38 40.17 39.96 39.75 39.54 39.33
OCH3
OH
OCH3
H3CO
O
HO
200 190 180 170 160 150 140 130 120 110 100 90 80 70 60 50 40 30 20 10 0

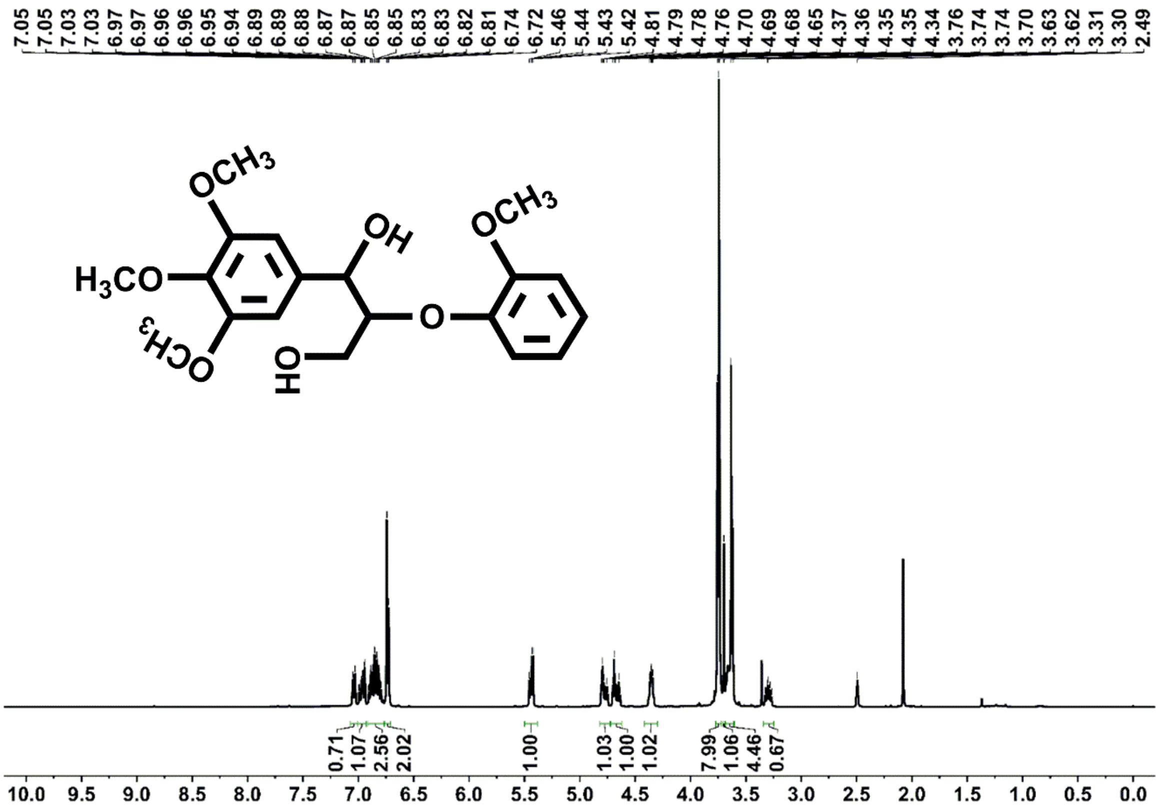
7.05
7.05
7.03
7.03
6.97
6.97
6.96
6.96
6.95
6.94
6.89
6.89
6.88
6.87
6.87
6.85
6.85
6.83
6.83
6.82
6.81
6.74
6.72
5.46
5.44
5.43
5.42
4.81
4.79
4.78
4.76
4.70
4.69
4.68
4.65
4.37
4.36
4.35
4.35
4.34
3.76
3.74
3.74
3.70
3.63
3.62
3.31
3.30
2.49
OCH3
OH
OCH3
H3CO
O
OCH3
HO
0.71
1.07
2.56
2.02
1.00
1.03
1.00
1.02
7.99
1.06
4.46
0.67
10.0 9.5 9.0 8.5 8.0 7.5 7.0 6.5 6.0 5.5 5.0 4.5 4.0 3.5 3.0 2.5 2.0 1.5 1.0 0.5 0.0

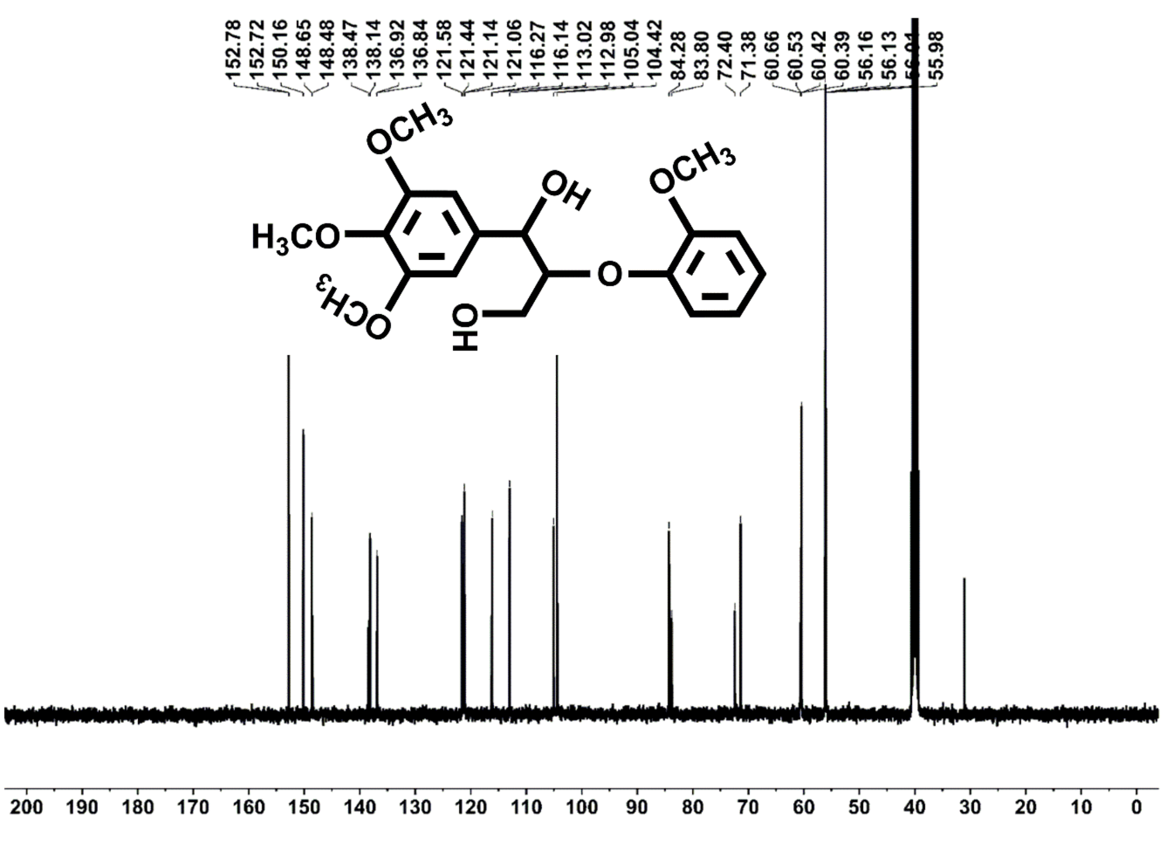
152.78
152.72
150.16
148.65
148.48
138.47
138.14
136.92
136.84
121.58
121.44
121.14
121.06
116.27
116.14
113.02
112.98
105.04
104.42
84.28
83.80
72.40
71.38
60.66
60.53
60.42
60.39
56.16
56.13
55.98
OCH3
OH
OCH3
H3CO
O
OCH3
HO
200 190 180 170 160 150 140 130 120 110 100 90 80 70 60 50 40 30 20 10 0

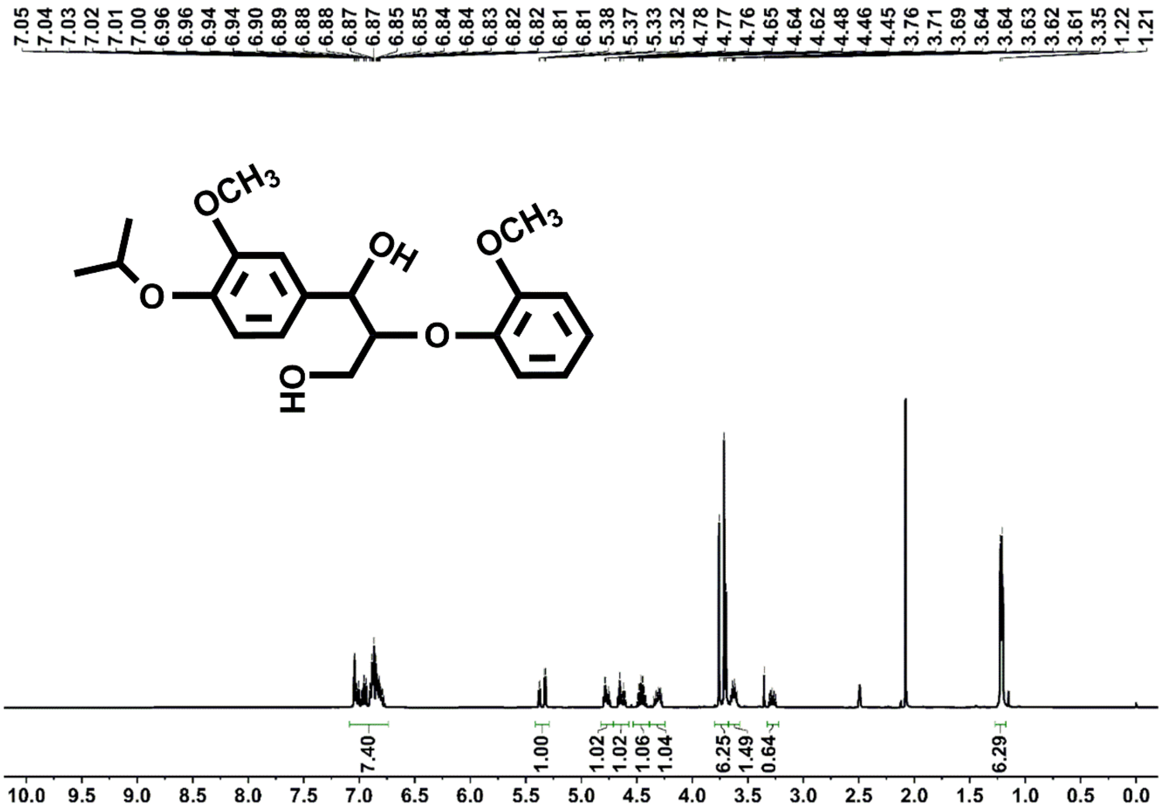
7.05 7.04 7.03 7.02 7.01 7.00 6.96 6.96 6.94 6.94 6.90 6.89 6.88 6.88 6.87 6.87 6.85 6.85 6.84 6.84 6.83 6.82 6.82 6.81 6.81 5.38 5.37 5.33 5.32 4.78 4.77 4.76 4.65 4.64 4.62 4.48 4.46 4.45 3.76 3.71 3.69 3.64 3.64 3.63 3.62 3.61 3.35 1.22 1.21
OCH3
OH
OCH3
O
O
HO
7.40
1.00
1.02
1.02
1.06
1.04
6.25
1.49
0.64
6.29
10.0 9.5 9.0 8.5 8.0 7.5 7.0 6.5 6.0 5.5 5.0 4.5 4.0 3.5 3.0 2.5 2.0 1.5 1.0 0.5 0.0

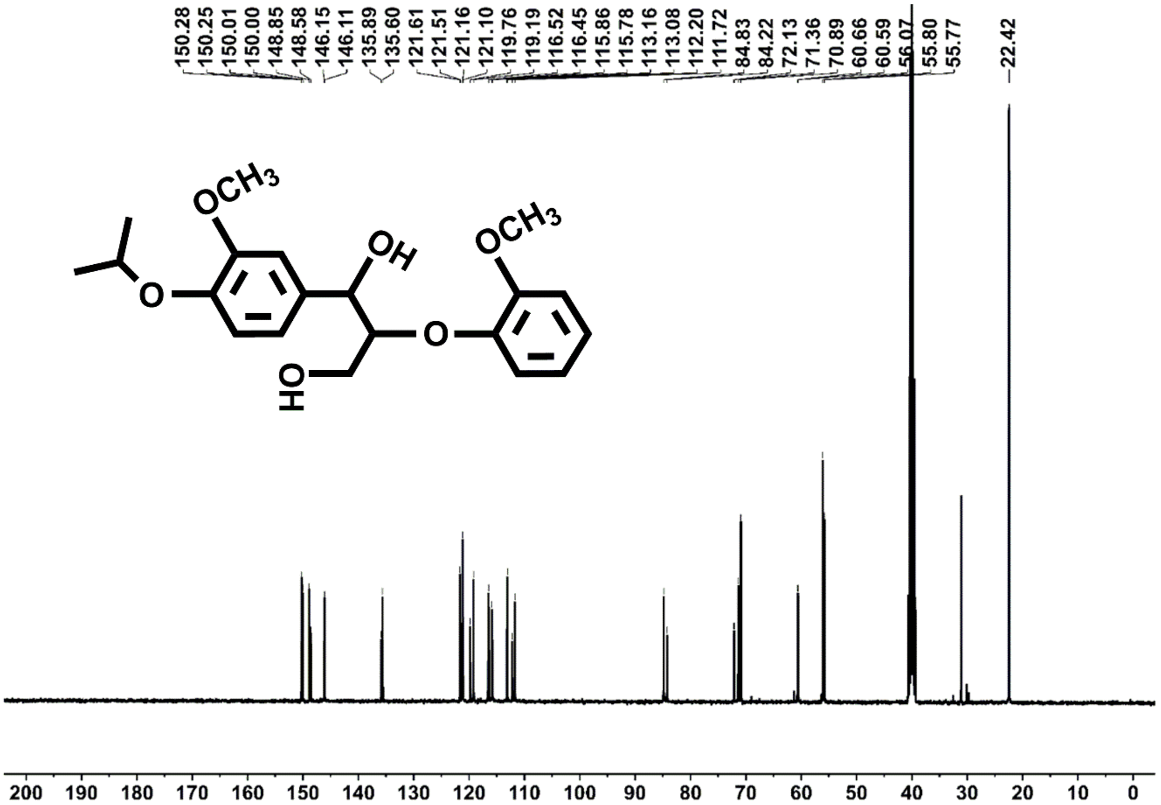
150.28 150.25 150.01 150.00 148.85 148.58 146.15 146.11 135.89 135.60 121.61 121.51 121.16 121.10 119.76 119.19 116.52 116.45 115.86 115.78 113.16 113.08 112.20 111.72 84.83 84.22 72.13 71.36 70.89 60.66 60.59 56.07 55.80 55.77
22.42
OCH3
OH
OCH3
O
O
HO
200 190 180 170 160 150 140 130 120 110 100 90 80 70 60 50 40 30 20 10 0

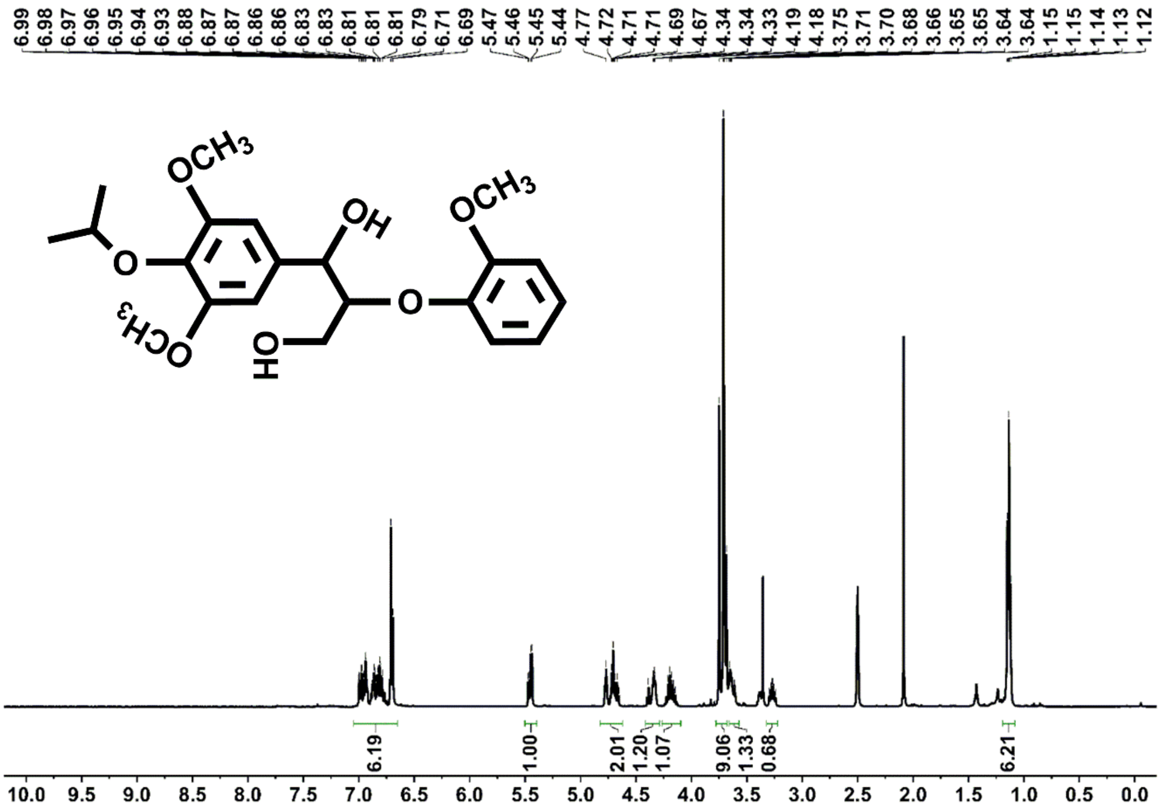
6.99 6.98 6.97 6.96 6.95 6.94 6.93 6.88 6.87 6.87 6.86 6.86 6.83 6.83 6.81 6.81 6.81 6.79 6.71 6.69 5.47 5.46 5.45 5.44 4.77 4.72 4.71 4.71 4.69 4.67 4.34 4.34 4.33 4.19 4.18 3.75 3.71 3.70 3.68 3.66 3.65 3.65 3.64 3.64 1.15 1.15 1.14 1.13 1.12
OCH3
OH
OCH3
O
OCH3
HO
6.19
1.00
2.01
1.20
1.07
9.06
1.33
0.68
6.21
10.0 9.5 9.0 8.5 8.0 7.5 7.0 6.5 6.0 5.5 5.0 4.5 4.0 3.5 3.0 2.5 2.0 1.5 1.0 0.5 0.0

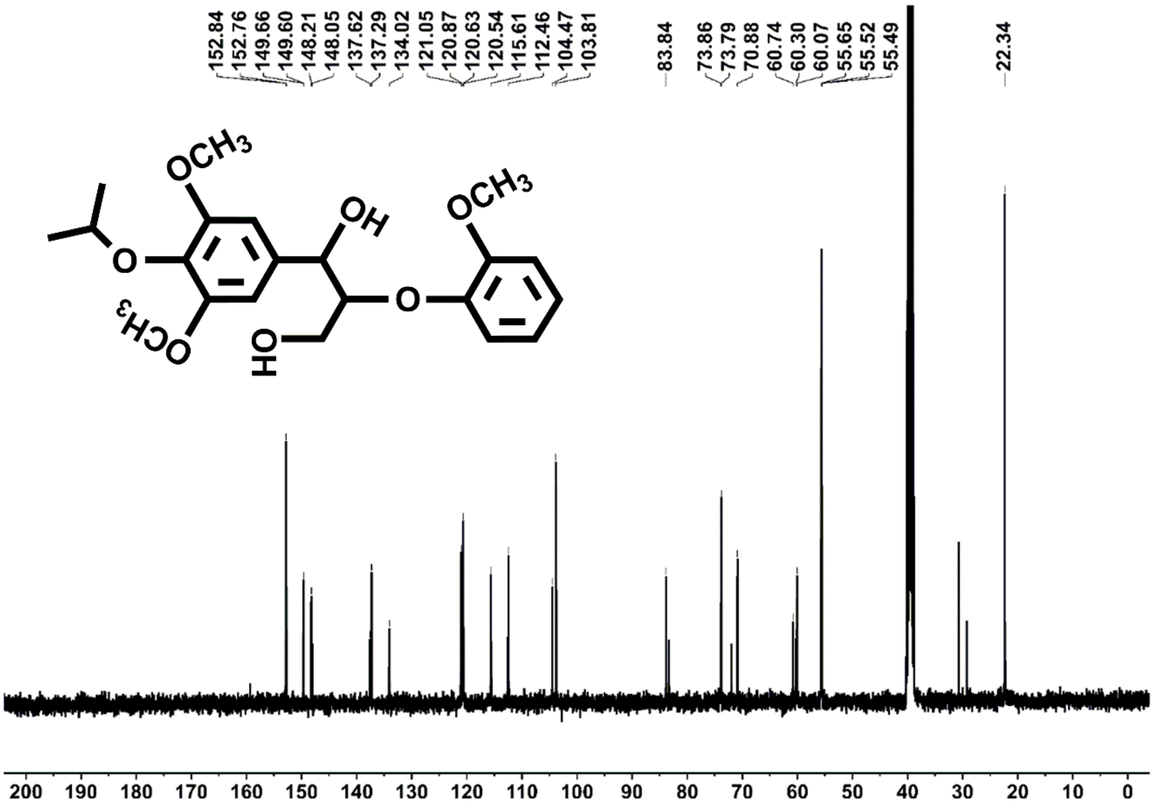
152.84 152.76 149.66 149.60 148.21 148.05 137.62 137.29 134.02 121.05 120.87 120.63 120.54 115.61 112.46 104.47 103.81
83.84
73.86 73.79 70.88 60.74 60.30 60.07 55.65 55.52 55.49
22.34
OCH3
OH
OCH3
O
OCH3
HO
200 190 180 170 160 150 140 130 120 110 100 90 80 70 60 50 40 30 20 10 0

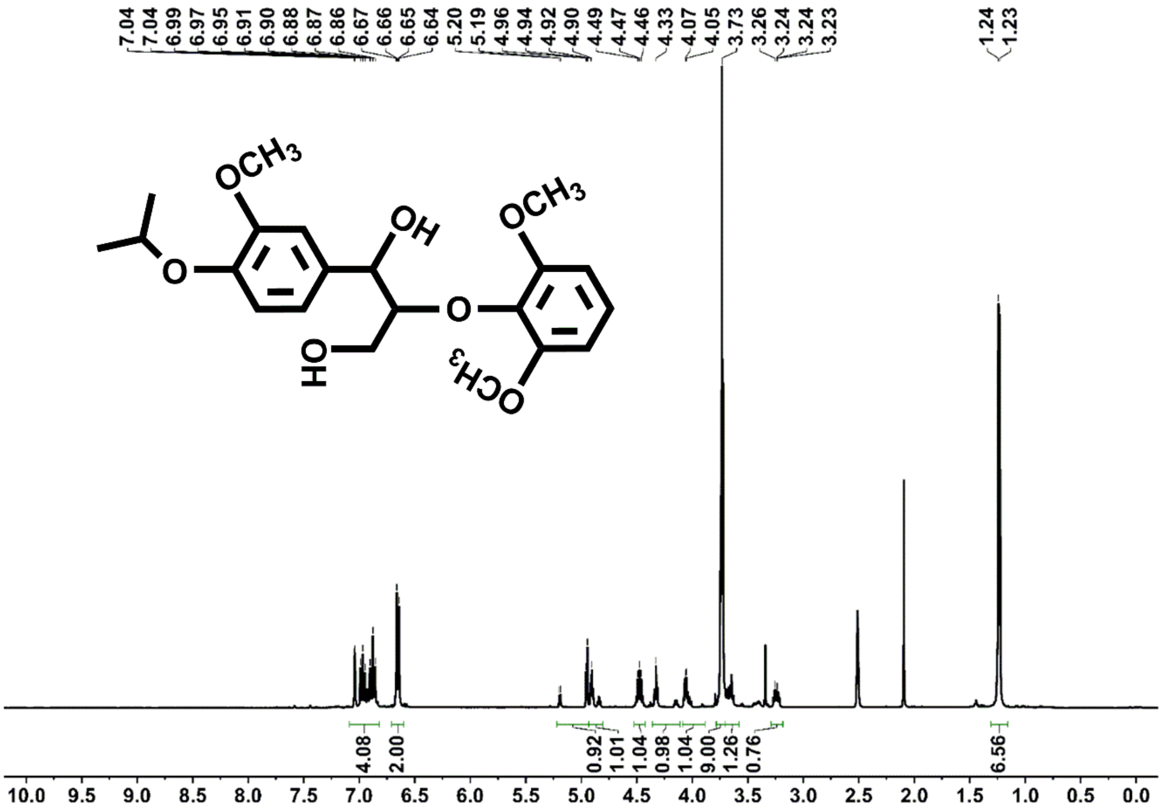
7.04 7.04 6.99 6.97 6.95 6.91 6.90 6.88 6.87 6.86 6.67 6.66 6.65 6.64 5.20 5.19 4.96 4.94 4.92 4.90 4.49 4.47 4.46 4.33 4.07 4.05 3.73 3.26 3.24 3.24 3.23
1.24 1.23
OCH3
OH
OCH3
O
O
HO
OCH3
4.08 2.00 0.92 1.01 1.04 0.98 1.04 9.00 1.26 0.76 6.56
10.0 9.5 9.0 8.5 8.0 7.5 7.0 6.5 6.0 5.5 5.0 4.5 4.0 3.5 3.0 2.5 2.0 1.5 1.0 0.5 0.0

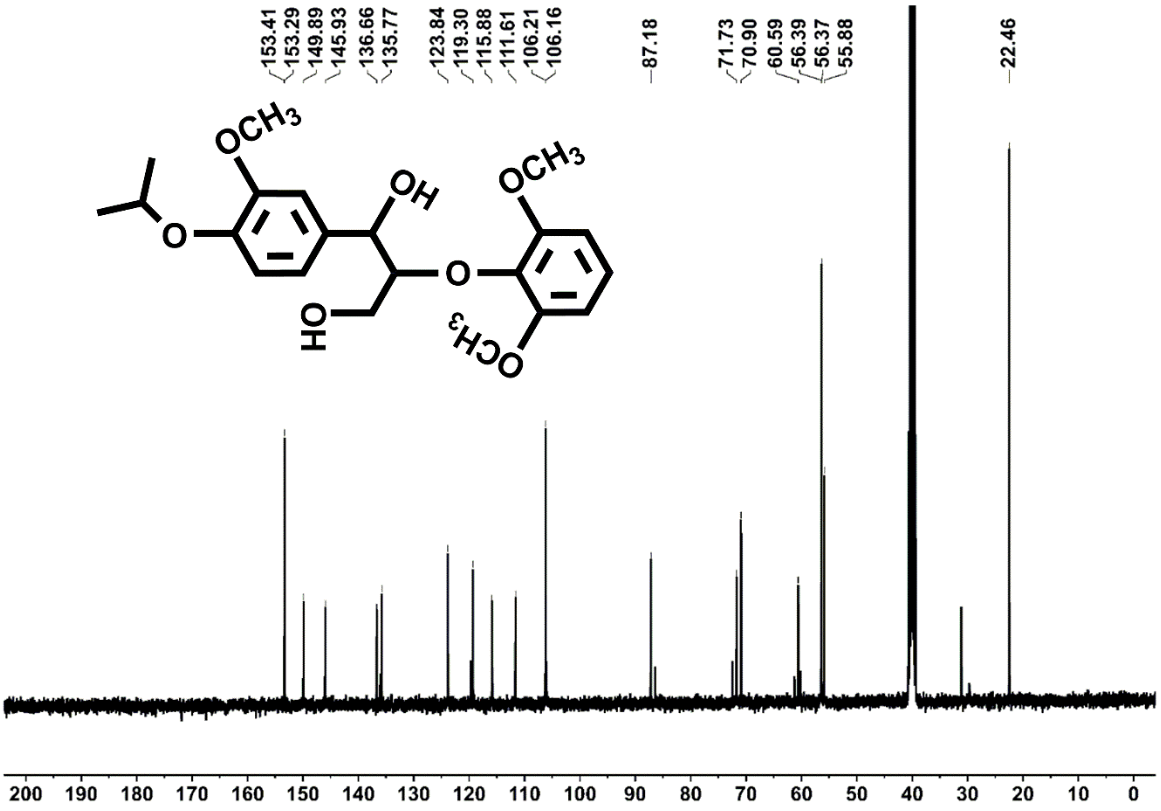
153.41 153.29 149.89 145.93 136.66 135.77 123.84 119.30 115.88 111.61 106.21 106.16
87.18
71.73 70.90 60.59 56.39 56.37 55.88
22.46
OCH3
OH
OCH3
O
O
HO
OCH3
200 190 180 170 160 150 140 130 120 110 100 90 80 70 60 50 40 30 20 10 0

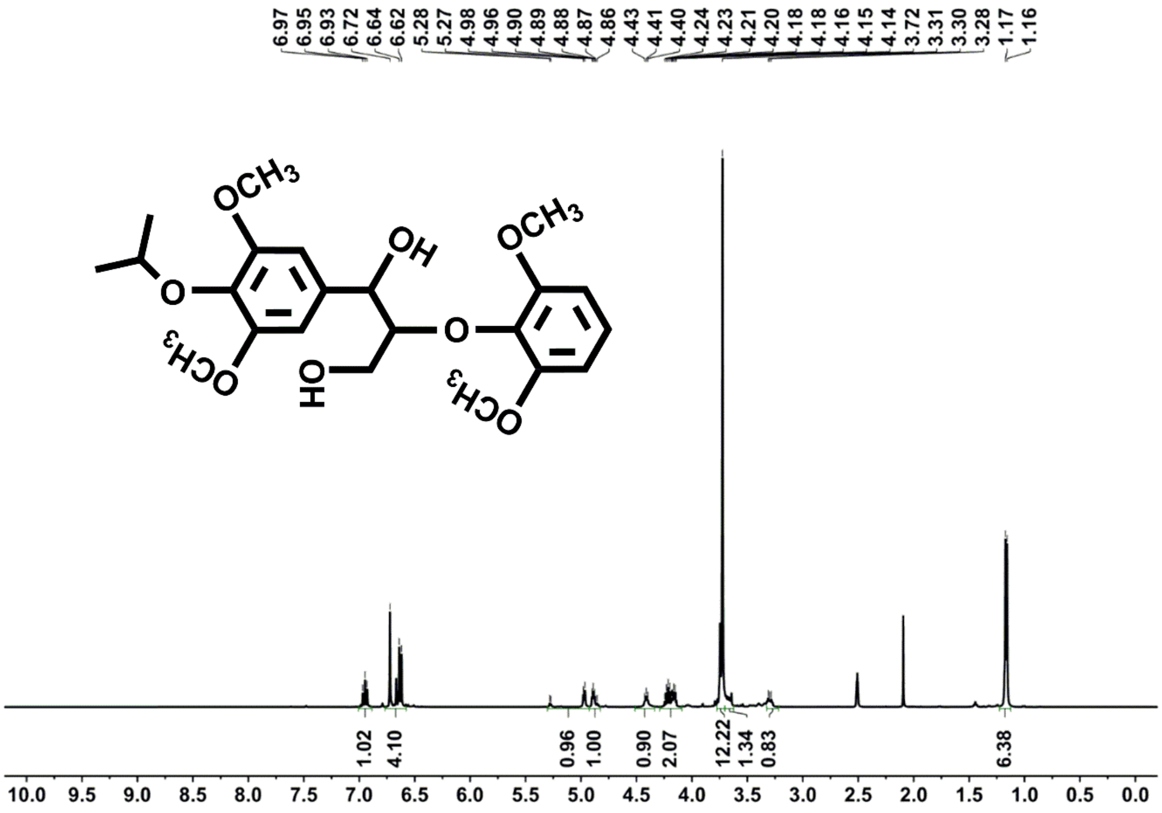
6.97
6.95
6.93
6.72
6.64
6.62
5.28
5.27
4.98
4.96
4.90
4.89
4.88
4.87
4.86
4.43
4.41
4.40
4.24
4.23
4.21
4.20
4.18
4.18
4.16
4.15
4.14
3.72
3.31
3.30
3.28
1.17
1.16
OCH3
OH
OCH3
O
O
OCH3
HO
OCH3
1.02
4.10
0.96
1.00
0.90
2.07
12.22
1.34
0.83
6.38
10.0 9.5 9.0 8.5 8.0 7.5 7.0 6.5 6.0 5.5 5.0 4.5 4.0 3.5 3.0 2.5 2.0 1.5 1.0 0.5 0.0

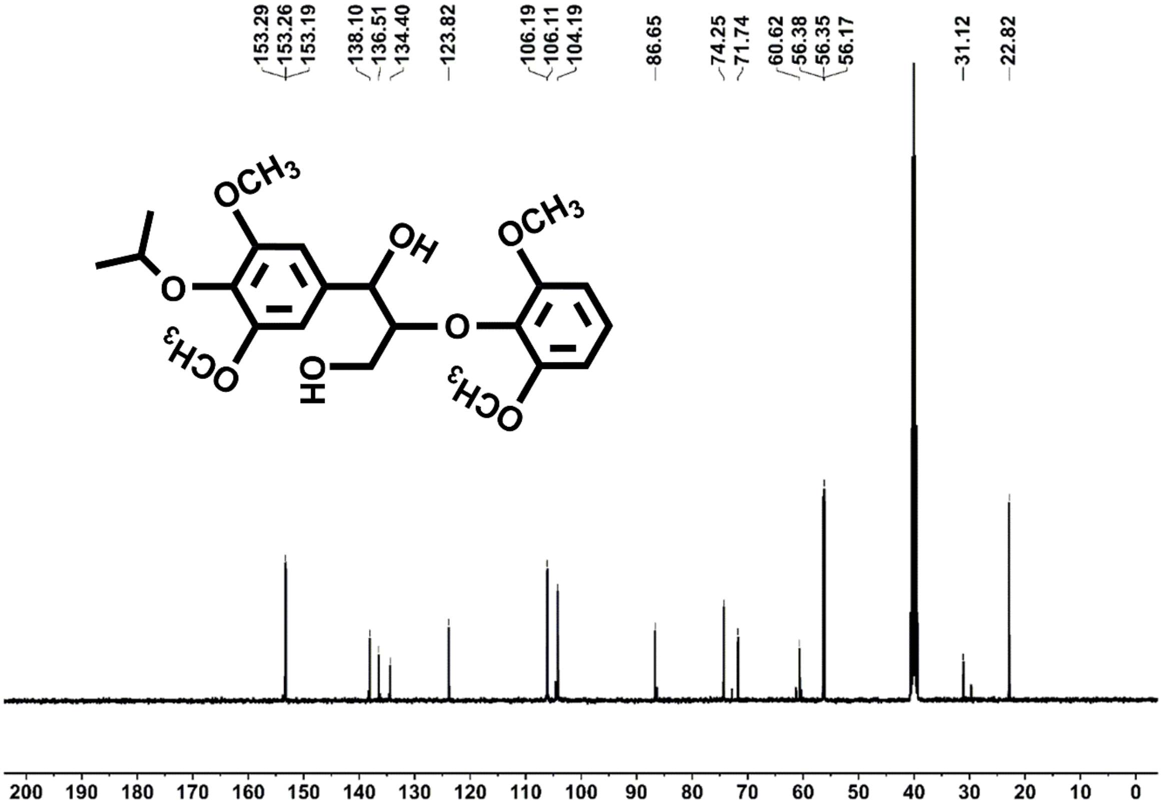
153.29
153.26
153.19
138.10
136.51
134.40
123.82
106.19
106.11
104.19
86.65
74.25
71.74
60.62
56.38
56.35
56.17
31.12
22.82
OCH3
OH
OCH3
O
O
OCH3
HO
OCH3
200 190 180 170 160 150 140 130 120 110 100 90 80 70 60 50 40 30 20 10 0

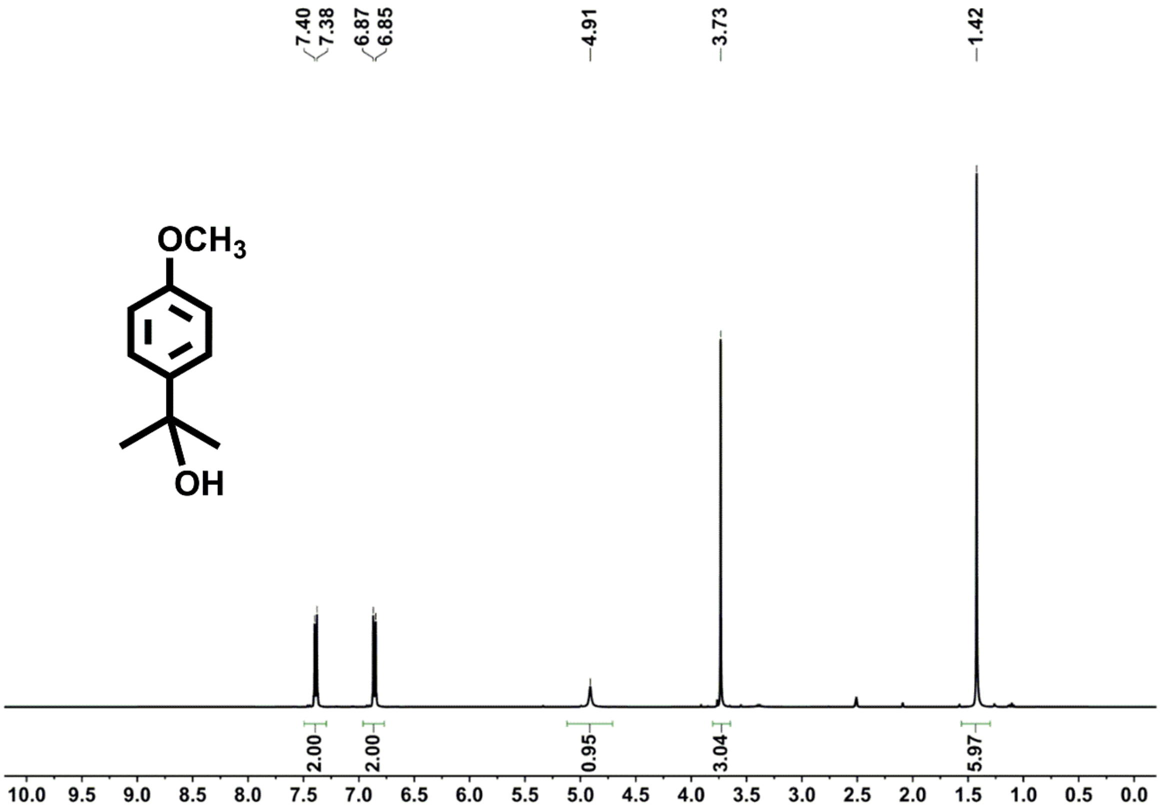
7.40
7.38
6.87
6.85
4.91
3.73
1.42
OCH3
OH
2.00
2.00
0.95
3.04
5.97
10.0 9.5 9.0 8.5 8.0 7.5 7.0 6.5 6.0 5.5 5.0 4.5 4.0 3.5 3.0 2.5 2.0 1.5 1.0 0.5 0.0

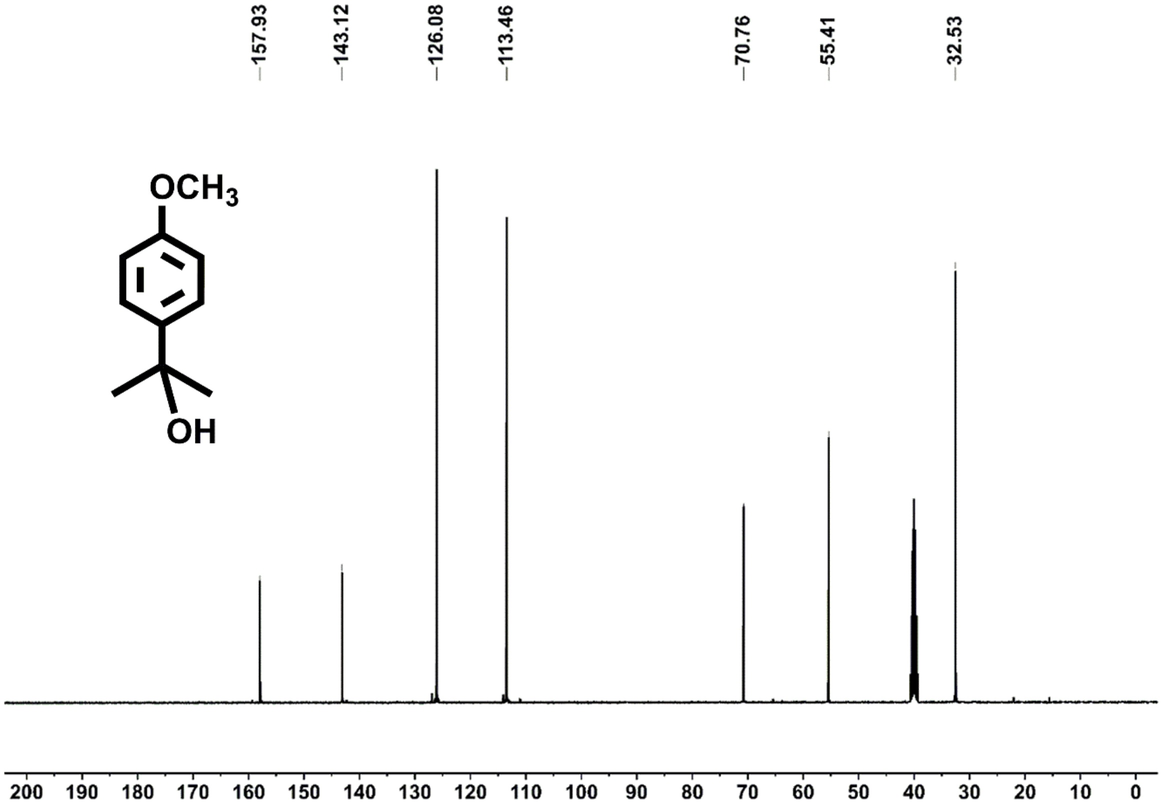
157.93
143.12
126.08
113.46
70.76
55.41
32.53
OCH3
OH
200 190 180 170 160 150 140 130 120 110 100 90 80 70 60 50 40 30 20 10 0

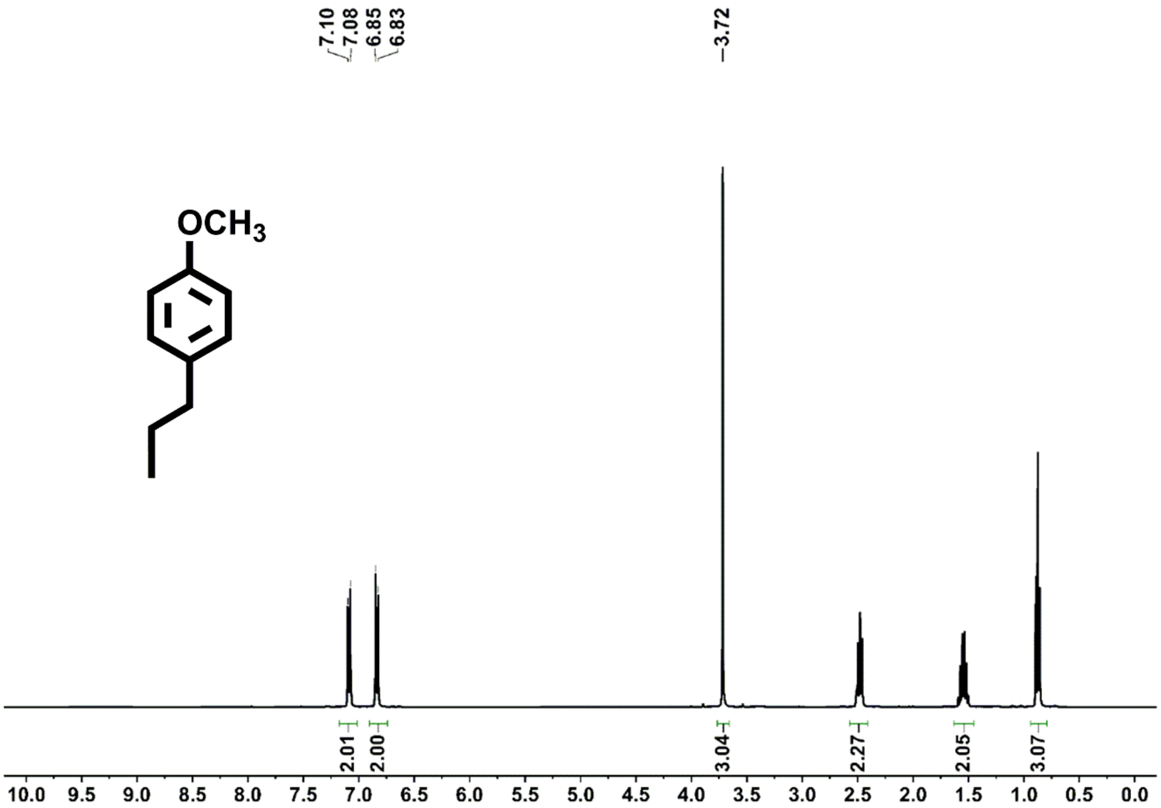
7.10
7.08
6.85
6.83
3.72
OCH3
2.01
2.00
3.04
2.27
2.05
3.07
10.0 9.5 9.0 8.5 8.0 7.5 7.0 6.5 6.0 5.5 5.0 4.5 4.0 3.5 3.0 2.5 2.0 1.5 1.0 0.5 0.0

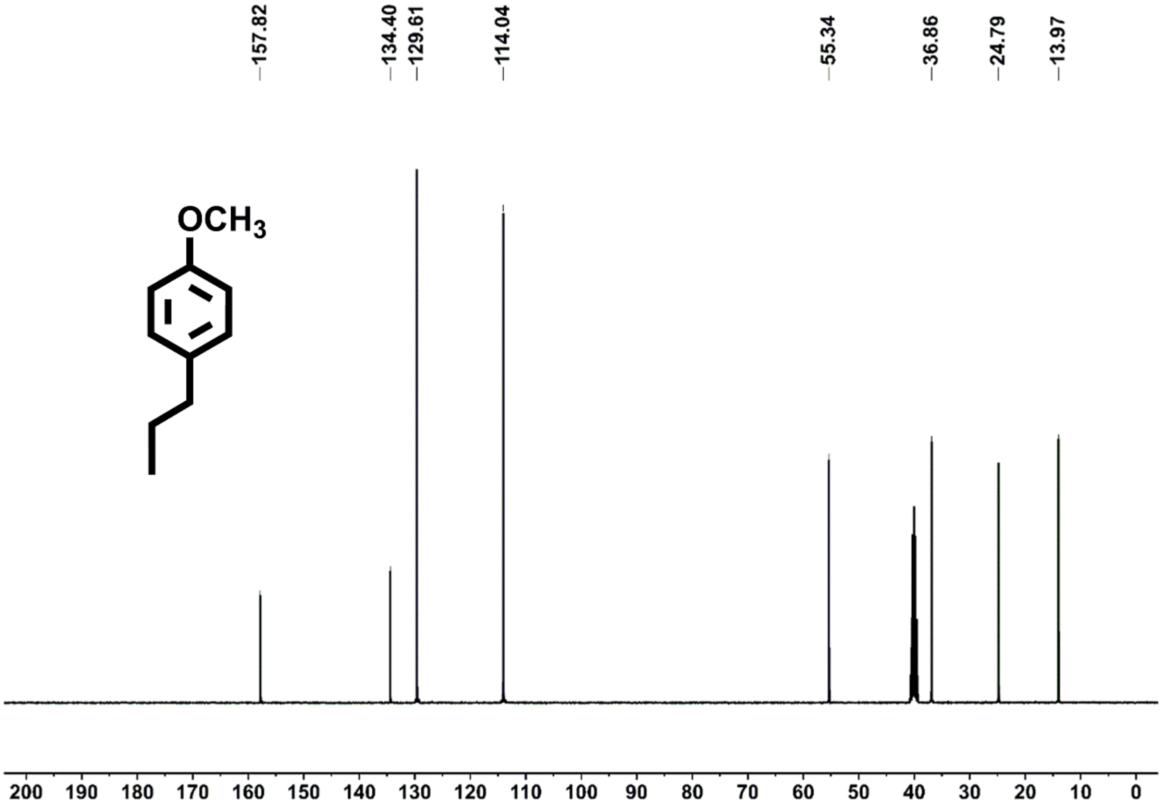
157.82
134.40
129.61
114.04
55.34
36.86
24.79
13.97
OCH3
200 190 180 170 160 150 140 130 120 110 100 90 80 70 60 50 40 30 20 10 0

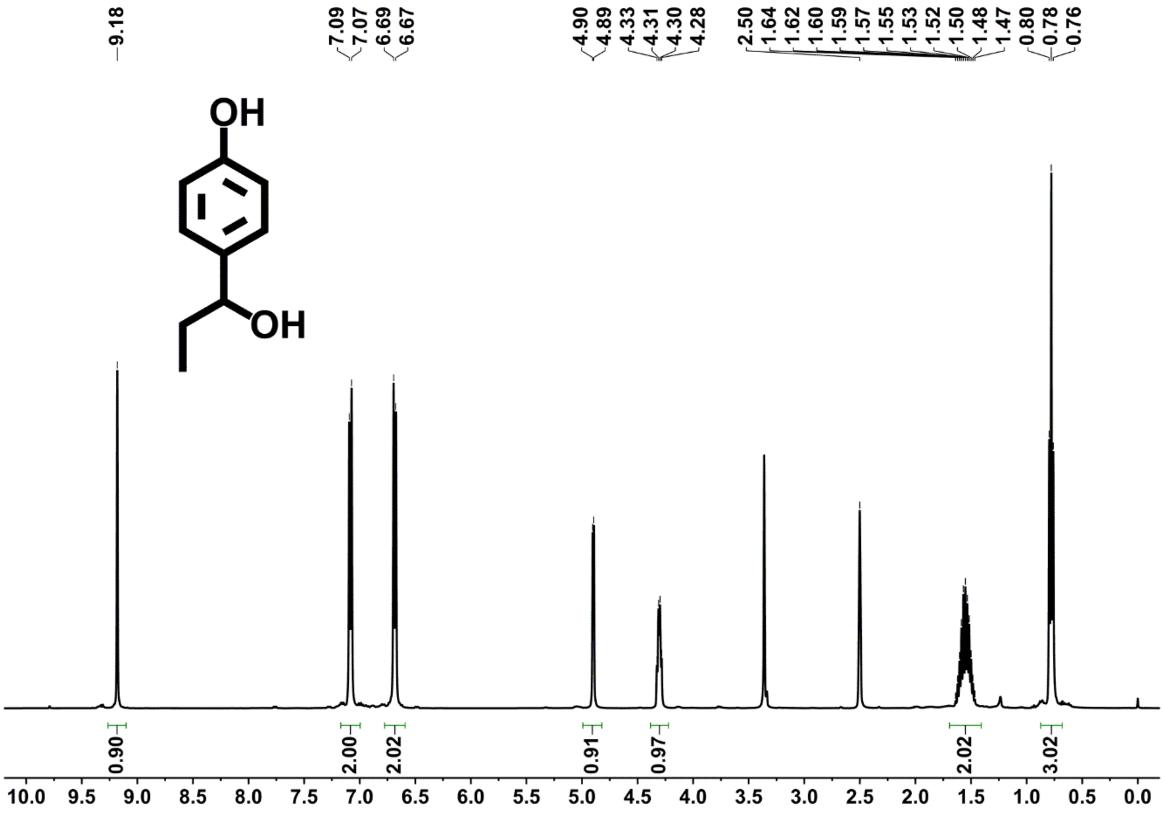
9.18
7.09
7.07
6.69
6.67
4.90
4.89
4.33
4.31
4.30
4.28
2.50
1.64
1.62
1.60
1.59
1.57
1.55
1.53
1.52
1.50
1.48
1.47
0.80
0.78
0.76
OH
OH
0.90
2.00
2.02
0.91
0.97
2.02
3.02
10.0 9.5 9.0 8.5 8.0 7.5 7.0 6.5 6.0 5.5 5.0 4.5 4.0 3.5 3.0 2.5 2.0 1.5 1.0 0.5 0.0

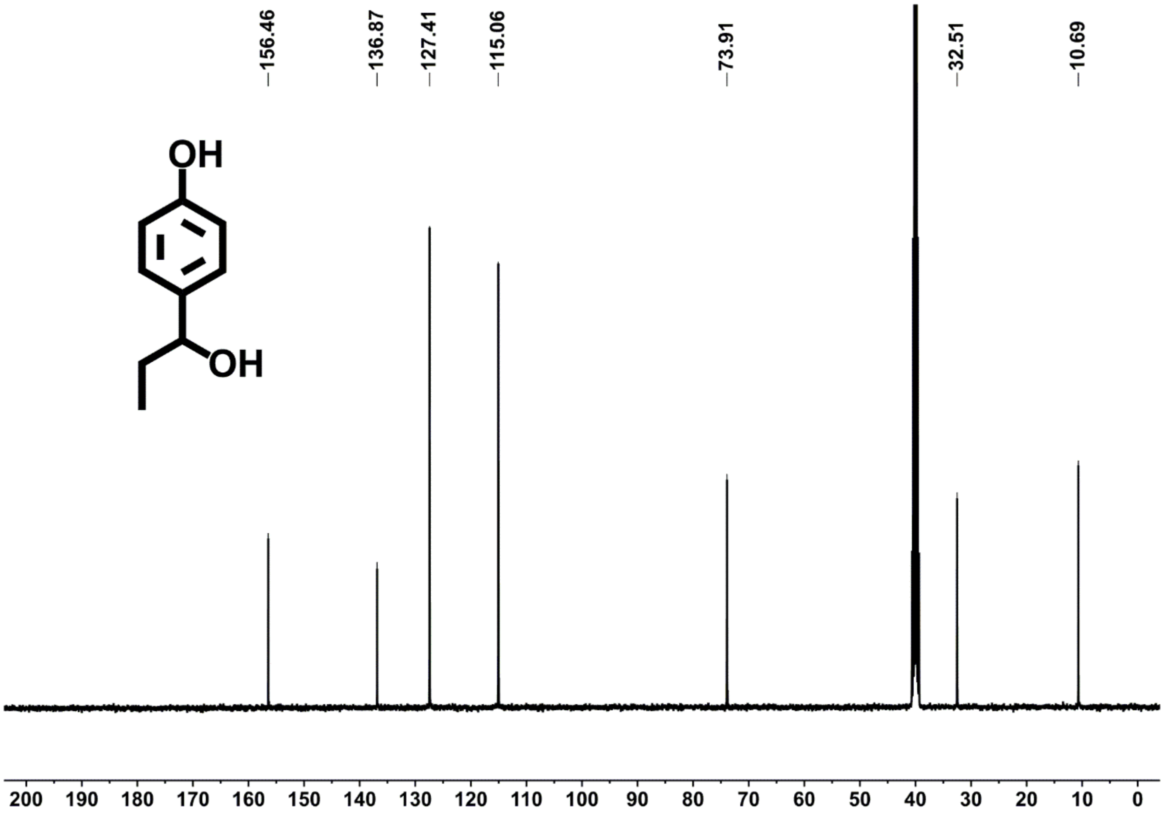
156.46
136.87
127.41
115.06
73.91
32.51
10.69
OH
OH
200 190 180 170 160 150 140 130 120 110 100 90 80 70 60 50 40 30 20 10 0

Supplementary References

1. Lancefield, C. S., Ojo, O. S., Tran, F. & Westwood, N. J. Isolation of Functionalized Phenolic Monomers through Selective Oxidation and C-O Bond Cleavage of the β-O-4 Linkages in Lignin. *Angew. Chem. Int. Ed.* **54**, 258-262 (2015).

2. Lee, S., Morimoto, H. & Williams, P. G. Synthesis of high specific radioactivity 3, 5-[3H6]dimethoxy-4-hydroxyacetophenone, an inducing compound of the vir gene in Agrobacterium tumefaciens. *J. Label Compd. Radiopharm.* **39**, 461-470 (1997).

3. Kresse, G. & Joubert, D. From ultrasoft pseudopotentials to the projector augmented-wave method. *Phys. Rev. B* **59**, 1758-1775 (1999).

4. Grimme, S., Antony, J., Ehrlich, S. & Krieg, H. A consistent and accurate ab initio parametrization of density functional dispersion correction (DFT-D) for the 94 elements H-Pu. *J. Chem. Phys.* **132**, 154104 (2010).

5. Henkelman, G., Uberuaga, B. P. & Jonsson, H. A climbing image nudged elastic band method for finding saddle points and minimum energy paths. *J. Chem. Phys.* **113**, 9901-9904 (2000).

6. Besora, M., Vidossich, P., Lledos, A., Ujaque, G. & Maseras, F. Calculation of reaction free energies in solution: A comparison of current approaches. *J. Phys. Chem. A* **122**, 1392-1399 (2018).

7. Gu, X. K., Liu, B. & Greeley, J. First-principles study of sructure sensitivity of ethylene glycol conversion on platinum. *ACS Catal.* **5**, 2623-2631 (2015).

8. Fruscella, W. Kirk-Othmer Encyclopedia of Chemical Technology (1999-2014) (Wiley, 2002).

9. McKetta Jr, J. J. Encyclopedia of Chemical Processing and Design: Volume 4-Asphalt Emulsion to Blending (CRC, 1977).
